# N‐Heterocyclic Carbene Analogues of Nucleophilic Phosphinidene Transition Metal Complexes

**DOI:** 10.1002/chem.202003099

**Published:** 2020-10-06

**Authors:** Adinarayana Doddi, Dirk Bockfeld, Thomas Bannenberg, Matthias Tamm

**Affiliations:** ^1^ Department of Chemical Sciences Indian Institute of, Science Education and Research Berhampur, Transit Campus, Govt. ITI Building Engineering School Road, Ganjam, Berhampur 760010 Odisha India; ^2^ Institut für Anorganische und Analytische Chemie Technische Universität Braunschweig Hagenring 30 38106 Braunschweig Germany

**Keywords:** iridium, N-heterocyclic carbenes, osmium, phosphinidenes, phosphorus ligands

## Abstract

Chloride abstraction from the complexes [(η^6^‐*p*‐cymene){(IDipp)P}MCl] (**2 a**, M=Ru; **2 b**, M=Os) and [(η^5^‐C_5_Me_5_){(IDipp)P}IrCl] (**3 b**, IDipp=1,3‐bis(2,6‐diisopropylphenyl)imidazolin‐2‐ylidene) with sodium tetrakis[3,5‐bis(trifluoromethyl)phenyl]borate (NaBAr^F^) in the presence of trimethylphosphine (PMe_3_), 1,3,4,5‐tetramethylimidazolin‐2‐ylidene (^Me^IMe) or carbon monoxide (CO) afforded the complexes [(η^6^‐*p*‐cymene){(IDipp)P}M(PMe_3_)]BAr^F^] (**4 a**, M=Ru; **4 b**, M=Os), [(η^6^‐*p*‐cymene){(IDipp)P}Os(^Me^IMe)]BAr^F^] (**5**) and [(η^5^‐C_5_Me_5_){(IDipp)P}IrL][BAr^F^] (**6**, L=PMe_3_; **7**, L=^Me^IMe; **8**, L=CO). These cationic N‐heterocyclic carbene‐phosphinidene complexes feature very similar structural and spectroscopic properties as prototypic nucleophilic arylphosphinidene complexes such as low‐field ^31^P NMR resonances and short metal‐phosphorus double bonds. Density functional theory (DFT) calculations reveal that the metal‐phosphorus bond can be described in terms of an interaction between a triplet [(IDipp)P]^+^ cation and a triplet metal complex fragment ligand with highly covalent σ‐ and π‐contributions. Crystals of the C−H activated complex **9** were isolated from solutions containing the PMe_3_ complex, and its formation can be rationalized by PMe_3_ dissociation and formation of a putative 16‐electron intermediate [(η^5^‐C_5_Me_5_)Ir{P(IDipp)}I][BAr^F^], which undergoes C−H activation at one of the Dipp isopropyl groups and addition along the iridium‐phosphorus bond to afford an unusual η^3^‐benzyl coordination mode.

## Graphical abstract

**N‐Heterocyclic** carbene‐phosphinidene complexes can be conceived as true nucleophilic phosphinidene complexes according to their spectroscopic, structural and electronic properties, since substitution of the aryl moiety in authentic neutral arylphosphinidene complexes of the type [ArP=ML_n_] by an N‐heterocyclic carbene affords the corresponding cationic complexes [(NHC)P=ML_n_]^+^ with equally short and covalent metal‐phosphorus double bonds.

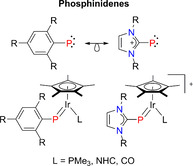

## Introduction

**Scheme 1:**
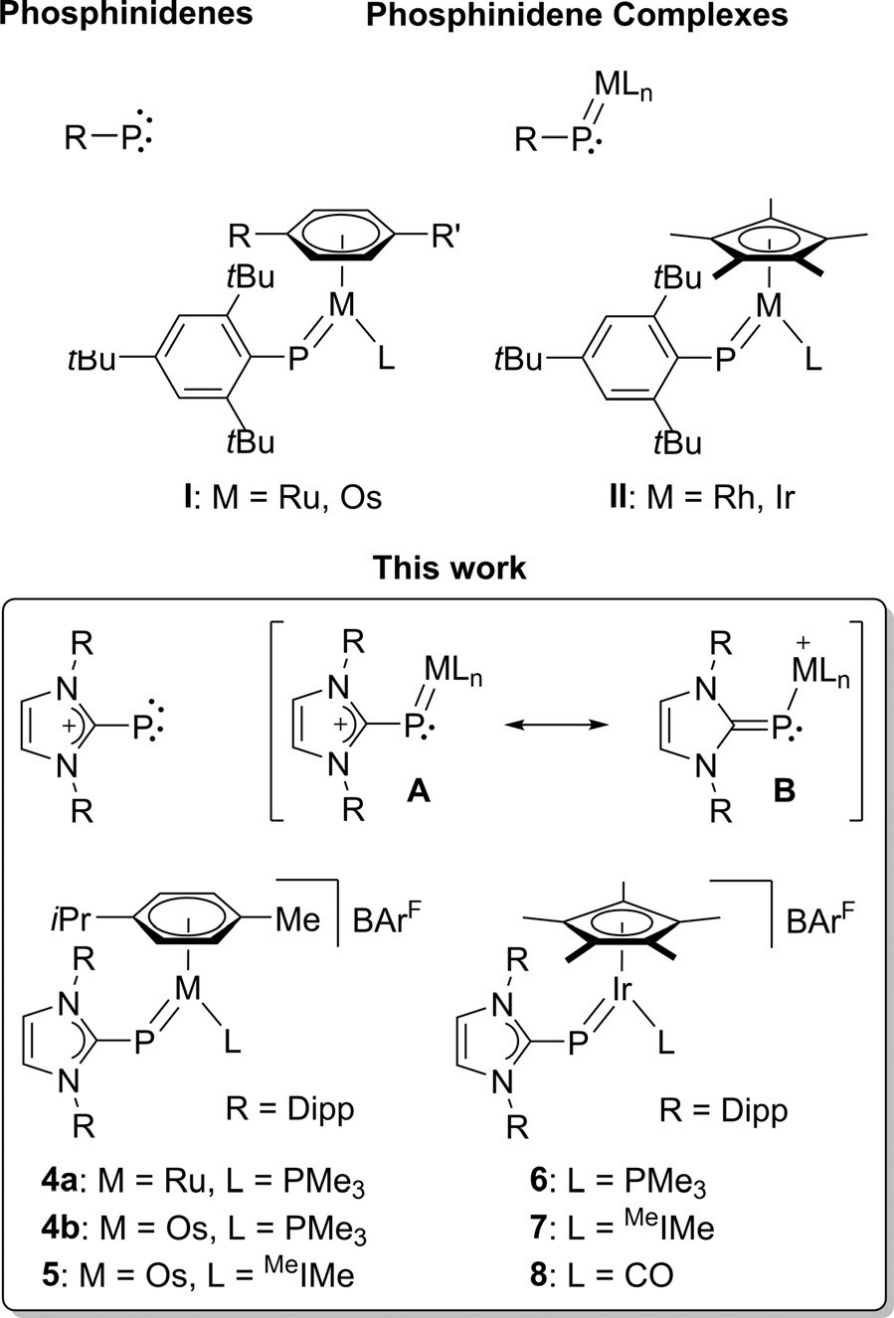
Phosphinidenes and phosphinidene metal complexes and their cationic N‐heterocyclic carbene analogues; Dipp = 2,4,6‐triisopropylphenyl, BAr^F^ = tetrakis[3,5‐bis(trifluoromethyl)phenyl]borate anion, ^Me^IMe = 1,3,4,5‐tetramethylimidazolin‐2‐ylidene.

The quest for terminal phosphinidene complexes [RP=ML_*n*_] has been one of the great challenges in organometallic chemistry,^[1]^ which has sparked the development of a carbene‐like chemistry with numerous applications in phosphinidene‐transfer and phosphorus‐element bond‐forming reactions.^[2]^ Similar to transition metal carbene complexes [R_2_C=ML_*n*_], phosphinidene complexes can be categorized into electrophilic (Fischer type) and nucleophilic (Schrock type) systems depending on the reactivity of the phosphorus atom, which is largely determined by the ancillary ligands (L).^[3]^ While transient electrophilic phosphinidene species such as [RP=M(CO)_*n*_] (*n*=5, M=Cr, Mo, W; *n*=4, M=Fe) exhibit high reactivity towards numerous substrates such as alkenes and alkynes, their usually more stable and isolable nucleophilic counterparts have found applicability only more recently by generation of reactive 16‐electron intermediates.^[4]^ Half‐sandwich ruthenium‐ and osmium‐arene [(η^6^‐Ar)(Mes*P)ML] (**I**; M=Ru, Os; Ar=benzene, *p*‐cymene) as well as rhodium‐ and iridium‐cyclopentadienyl complexes [(η^5^‐C_5_Me_5_)(Mes*P)ML] (**II**; M=Rh, Ir; Mes*=2,4,6‐tri‐*tert*‐butylphenyl; L=PR_3_, NHC, CO), developed in particular by Lammertsma and co‐workers, represent a prominent and well‐studied class of nucleophilic phosphinidene complexes, which are accessible by dehydrochlorination and ligation (L) of the corresponding phosphine precursors [(η^6^‐Ar)MCl_2_(PH_2_Mes*)] (M=Ru, Os) or [(η^5^‐C_5_Me_5_)MCl_2_(PH_2_Mes*)] (M=Rh, Ir), respectively.[^5^, ^6^, ^7^, ^8^, ^9^, ^10^] The resulting bent phosphinidene complexes of type **I** and **II** feature genuine metal‐phosphorus double bonds with *E* or *Z* conformation depending on the ligand size and are characterized by very large ^31^P NMR chemical shifts in the range 560–900 ppm (Scheme 1).[^4^, ^5^, ^6^, ^7^, ^8^, ^9^, ^10^, ^11^, ^12^]

**Scheme 2:**
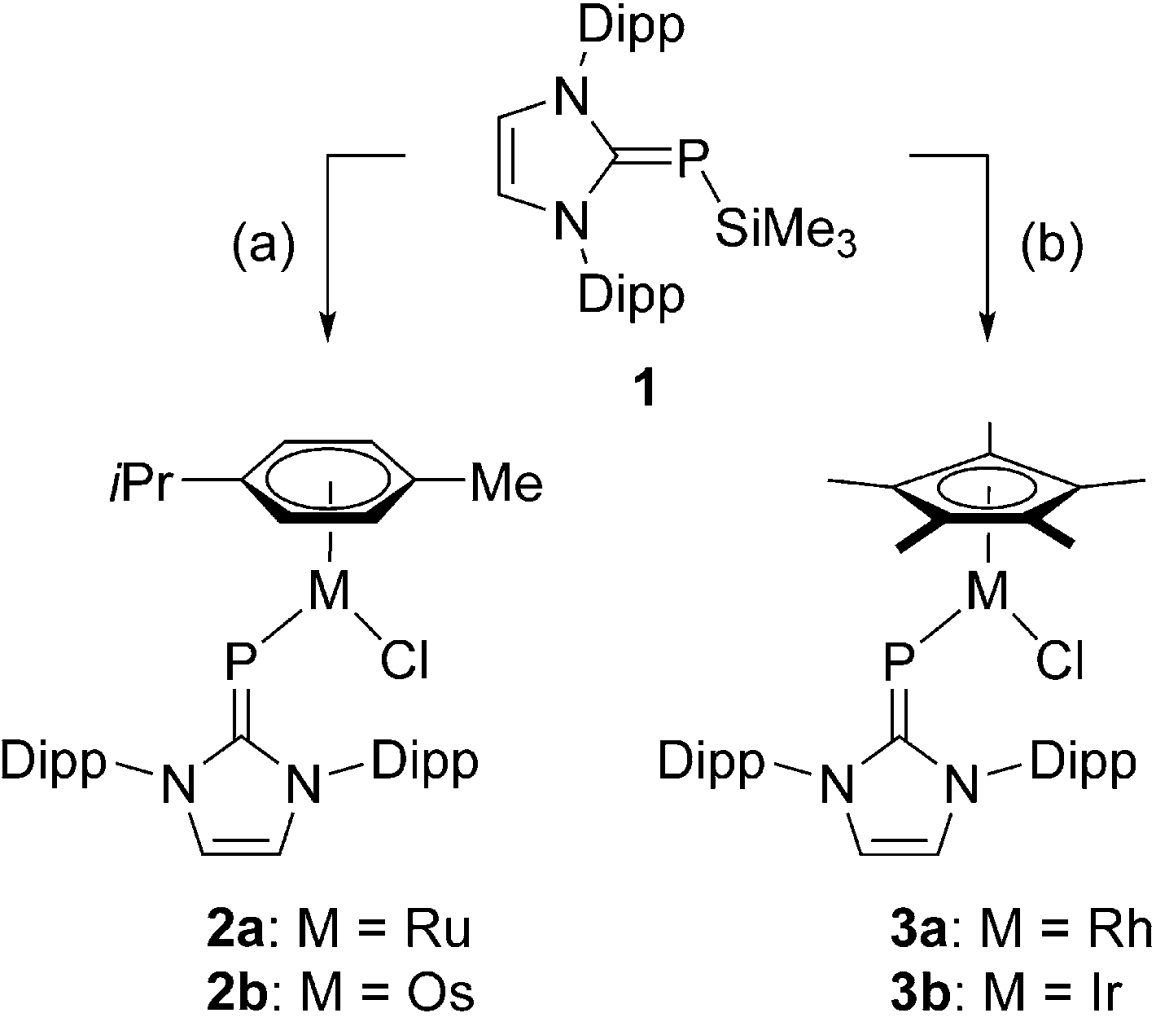
Preparation of N‐heterocyclic carbene‐phosphinidenide complexes; (a) 0.5 equiv[(η^6^‐*p*‐cymene)MCl_2_] (M=Ru, Os), (b) 0.5 equiv [(η^5^‐ C_5_Me_5_)MCl_2_] (M=Rh, Ir).

Similar structural and spectroscopic features were reported by our group for the related N‐heterocyclic carbene (NHC) species **2** and **3** (Scheme 2), which were prepared by reaction of the trimethylsilylphosphinidene adduct (IDipp)PSiMe_3_ (**1**, IDipp=1,3‐bis(2,6‐diisopropylphenyl)imidazolin‐2‐ylidene)^[13]^ with the dimeric complexes [(η^6^‐Ar)MCl_2_]_2_ (M=Ru, Os) or [(η^5^‐C_5_Me_5_)MCl_2_]_2_ (M=Rh, Ir).[^14^, ^15^] Alternatively, these complexes could also be generated, albeit in lower yield, by dehydrochlorination of the N‐heterocyclic carbene‐phosphinidene complexes [(η^6^‐Ar)MCl_2_{PH(IDipp)}] (M=Ru, Os) or [(η^5^‐C_5_Me_5_)MCl_2_{PH(IDipp)}] (M=Rh, Ir) in the presence of 1,8‐diazabicyclo[5.4.0]undec‐7‐ene (DBU).^[15]^ All complexes **2** and **3** exhibit short metal‐phosphorus bonds and low‐field ^31^P NMR resonances, revealing their relationship with the terminal phosphinidene complexes **I** and **II**. Formally, the (IDipp)P ligand in **2** and **3** can be conceived as a monoanionic NHC‐phosphinidenide ligand acting as a 2σ,2π‐electron donor. In view of the high covalency of the metal‐phosphorus bond and in analogy with phosphinidene complexes, however, the ligand can also be described as a cationic NHC‐phosphinidene species, with the degree of metal‐to‐ligand π‐backbonding depending on the π‐accepting ability of the NHC moiety as illustrated by the mesomeric structures **A** and **B** in Scheme 1.^[16]^ Accordingly, substitution of the Mes* group in **I** and **II** by IDipp affords cationic complexes such as **4**–**8**, which are the subject of this contribution. We envisaged that chloride abstraction from **2** and **3** would generate a putative cationic 16‐electron intermediate, which could be trapped and stabilized by addition of suitable ligands such as carbon monoxide (CO), phophines (PR_3_) or N‐heterocyclic carbenes. The similarity of the resulting complexes **4**–**8** with authenticated phosphinidene species of type **I** and **II** would therefore allow to further analyze the phosphinidene character of this novel class of (NHC)P ligands.

(NHC)P ligands are representatives of the growing class of NHC adducts of main group elements which are used as ligands in transition metal chemistry,^[17]^ and NHC phosphorus compounds have received particular attention.^[18]^ Numerous transition metal complexes containing NHC‐phosphinidene adducts of the type (NHC)PR (R=H, alkyl, aryl) have been described,^[19]^ whereas (NHC)P complexes beyond **2** and **3** are hitherto hardly known.[^14^, ^15^] To the best of our knowledge, Grützmacher described the homoleptic mercury complexes [{(IDipp)P}_2_Hg] and [{(SIDipp)P}_2_Hg] (SIDipp=bis(2,6‐diisopropylphenyl)imidazolidin‐2‐ylidene)^[13]^ as the only other transition metal complexes, which were prepared from HgCl_2_ and the corresponding phosphinidene adducts (IDipp)PH and (SIDipp)PH in the presence of two equivalents of DBU.^[20]^ In addition, the group of von Hänisch employed the potassium salts [(SIMes)PK] (SIMes=bis(2,4,6‐trimethylphenyl)imidazolidin‐2‐ylidene)^[13]^ and [(SIDipp)PK] for the preparation of main group metal complexes,^[21]^ with the tetrylene derivatives [{(SIDipp)P}_2_M] (M=Ge, Sn, Pb) representing the most recent examples.^[22]^ N‐Heterocyclic carbene‐stabilized germanium and tin analogues of heavier nitriles of the type [(^Mes^Ter)EP(IDipp)] (E=Ge, Sn; ^Mes^Ter=2,6‐bis(2,4,6‐trimethylphenyl)phenyl) were recently isolated by Inoue and co‐workers by reacting dimeric [(^Mes^Ter)ECl]_2_ with **1**, which resembles the reaction with the dimeric metal chlorides as shown in Scheme 2.^[23]^ Naturally, metal‐phosphorus multiple bonding does not play any significant role in these late transition or *p*‐block metal complexes, which is different from the bonding situation that will be uncovered for the phosphinidene‐type complexes **4**–**8** presented in this contribution.

## Results and Discussion

### Synthesis and characterization of cationic ruthenium and osmium (NHC)P complexes

**Scheme 3:**
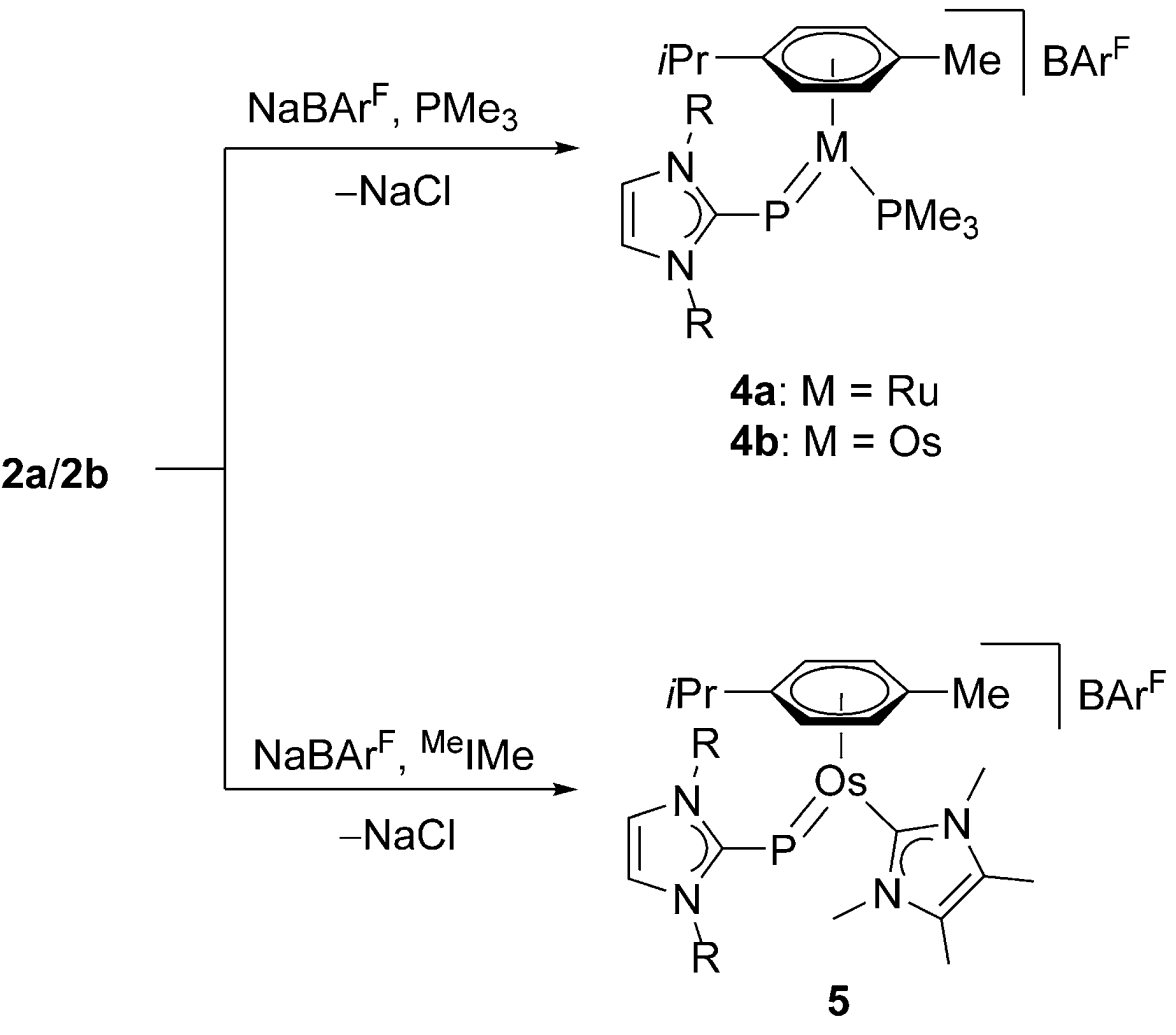
Synthesis of cationic ruthenium and osmium (NHC)P complexes; R=Dipp=2,6‐diisopropylphenyl.

The reaction of the complexes [(η^6^‐*p*‐cymene){(IDipp)P}MCl] (**2 a**, M=Ru; **2 b**, M=Os) with sodium tetrakis[3,5‐bis(trifluoromethyl)phenyl]borate (NaBAr^F^) in the presence of trimethylphosphine (PMe_3_) in fluorobenzene at room temperature afforded the complexes [(η^6^‐*p*‐cymene){(IDipp)P}M(PMe_3_)]BAr^F^] (**4 a**, M=Ru; **4 b**, M=Os) as purple solids in 86 % and 75 % yield, respectively (Scheme 3). The ^1^H NMR spectra (in [D_8_]THF) reveal the presence of the PMe_3_ ligand by showing a doublet at 1.42 ppm (**4 a**, ^2^ *J*_PH_=9.5 Hz) and 1.54 ppm (**4 b**, ^2^ *J*_PH_=10.7 Hz), while the ^31^P NMR spectra display two doublets at 592.9 and −15.1 ppm (^2^ *J*_PP_=41.3 Hz) in case of **4 a** and at 438.9 and −37.1 ppm (^2^ *J*_PP_=99.0 Hz) in case of **4 b**. These low‐ and high‐field ^31^P NMR resonances can be assigned to the (IDipp)P and PMe_3_ phosphorus nuclei, respectively. The corresponding resonances for the (IDipp)P unit in **2 a** (531.5 ppm) and **2 b** (354.3 ppm) are found at higher field,^[15]^ whereas related complexes of type **I** (Scheme 1) afford resonances for the phosphinidene moiety at even significantly lower field, e.g., 800.5/−14.0 ppm (^2^ *J*_PP_=37 Hz) for [(η^6^‐C_6_H_6_)(Mes*P)Ru(PMe_3_)], 845.9/40.7 ppm (^2^ *J*_PP_=40 Hz) for [(η^6^‐C_6_H_6_)(Mes*P)Ru(PPh_3_)], 837.3/40.1 ppm (^2^ *J*_PP_=44 Hz) for [(η^6^‐*p*‐cymene)(Mes*P)Ru(PPh_3_)], 673.6/19.4 ppm (^2^ *J*_PP_=72 Hz) for [(η^6^‐C_6_H_6_)(Mes*P)Os(PPh_3_)], and 667.5/18.8 ppm (^2^ *J*_PP_=80 Hz) for [(η^6^‐*p*‐cymene)(Mes*P)Os(PPh_3_)].^[6]^ All of these mixed phosphinidene‐phosphine complexes were shown to display an *E* configuration of the metal‐phosphorus double bond, and the large ^2^ *J*_PP_ coupling constants observed for **4 a** and **4 b** also suggest an *E* configuration in both cases, which differs from the *Z* forms established for **2 a** and **2 b** in solution and in the solid state.^[15]^

**Figure 1:**
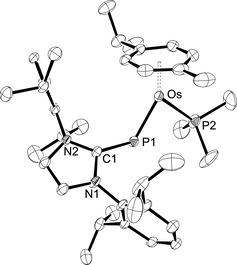
ORTEP diagram of the cationic complex in **4 b**⋅THF with thermal displacement parameters drawn at the 50 % probability level. Hydrogen atoms, the BAr^F^ counterion and the solvent molecule were omitted for clarity. Selected bond lengths and angles are assembled in Table 1.

Single crystals of **4 a**⋅C_6_H_5_F and **4 b**⋅THF were obtained from fluorobenzene or fluorobenzene/THF solution, respectively. X‐ray diffraction analysis allowed to determine the molecular structures, which are shown in Figure 1 for the cationic part of **4 b** and in Figure S64 of the Supporting Information for **4 a**. Pertinent structural data are assembled in Table 1. In agreement with the spectroscopic data, *E* configurations are confirmed for the bent (IDipp)P ligands with Ru‐P1‐C1=120.15(9)° (**4 a**) and Os‐P1‐C1=121.44(10)° (**4 b**). The metal atoms display a pseudotrigonal planar environment (two‐legged piano‐stool geometry) with acute P1‐M‐P2 angles of 81.88(3)° (**4 a**) and 81.79(3)° (**4 b**). The imidazole planes adopt intermediate positions between horizontal and vertical orientations as indicated by torsion angles of Ru‐P1‐C1‐N1=129.5(2)° (**4 a**) and Os‐P1‐C1‐N1=131.0(2)° (**4 b**). Both complexes have a short and a long metal‐phosphorus bond of 2.1929(7)/2.3387(8) Å (Ru−P1/Ru−P2) in **4 a** and 2.2005(9)/2.3281(9) Å (Os−P1/Os−P2) in **4 b**, which are illustrative of double and single bond character, respectively. In fact, these structural features are almost identical with those found for the neutral phosphinidene congeners **I** such as [(η^6^‐C_6_H_6_)(Mes*P)Ru(PPh_3_)] (2.1988(6)/2.3289(6) Å) and [(η^6^‐*p*‐cymene)(Mes*P)Os(PPh_3_)] (2.2195(7)/2.3054(6) Å).^[6]^ Very similar Ru−P bond lengths and P‐Ru‐P bond angles were also reported for the tricyclohexylphosphine (PCy_3_) derivatives [(η^6^‐Ar)(Mes*P)Ru(PCy_3_)] (Ar=C_6_H_6_, *p*‐cymene).^[12]^ It should be noted that the metal‐phosphorus bond lengths in the chlorido complexes **2 a** and **2 b** of 2.2099(6) Å (Ru−P) and 2.2230(19) Å (Os−P) are equally short, which has been ascribed to the presence of covalent double bonds.^[15]^

**Table 1:** Selected spectroscopic and structural data of complexes **2**–**8**.

Compound	M	L	*δ* ^31^P [ppm]	^2^ *J*_PP_ [Hz]	M−P [Å]	M−L [Å]	P−C1 [Å]	M‐P‐C1 [°]	P‐M‐L [°]
**2 a**	Ru	Cl	531.5	–	2.2099(6)	2.3956(6)	1.824(2)	112.80(7)	94.24(2)
**2 b**	Os	Cl	354.3	–	2.2230(19)	2.3808(19)	1.829(7)	113.0(2)	93.67(7)
**3 b**	Ir	Cl	353.7	–	2.1966(8)	2.3506(8)	1.833(3)	111.69(10)	95.00(3)
**4 a**	Ru	PMe_3_	592.9/−15.1	41.3	2.1929(7)	2.3387(8)	1.848(3)	120.15(9)	81.88(3)
**4 b**	Os	PMe_3_	438.9/−37.1	99.0	2.2005(9)	2.3281(9)	1.855(3)	121.44(10	81.79(3)
**5**	Os	^Me^IMe	394.3	–	2.2119(5)	2.0916(19)	1.8519(19)	117.84(6)	80.89(5)
**6**	Ir	PMe_3_	454.9/−33.1	106.7	–	–	–	–	–
**7**	Ir	^Me^IMe	399.0 (*E*)/483.8 (*Z*)	–	2.193(3)/2.205(2)^[a]^	2.050(12)/2.050(9)^[a]^	1.861(11)/1.845(10)^[a]^	120.3(4)/121.1(3)^[a]^	82.6(3)/81.0(3)^[a]^
**8**	Ir	CO	596.9	–	2.1905(10)	1.870(4)	1.824(3)	113.73(11)	98.54(11)

[a] For two crystallographically independent molecules of the *E* isomer.

**Figure 2:**
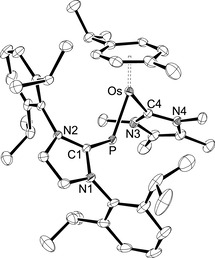
ORTEP diagram of the cationic complex in **5** with thermal displacement parameters drawn at the 50 % probability level. Hydrogen atoms and the BAr^F^ counterion were omitted for clarity. Selected bond lengths and angles are assembled in Table 1.

The reaction of the osmium complex **2 b** with NaBAr^F^ in fluorobenzene in the presence of the NHC 1,3,4,5‐tetramethylimidazolin‐2‐ylidene (^Me^IMe) furnished the complex [(η^6^‐*p*‐cymene){(IDipp)P}M(^Me^IMe)]BAr^F^ (**5**) as a red‐brown solid in 70 % yield (Scheme 1). The ^1^H NMR spectrum indicates the presence of the ^Me^IMe ligand by showing two singlets for the methyl groups at 2.08 and 3.24 ppm, and the diagnostic ^31^P NMR resonance is observed as a sharp singlet at 394.3 ppm. Similar to the trends observed for **4 a** and **4 b**, this resonance is at lower field compared to **2 b** (354.3 ppm), but at higher field compared to the phosphinidene complex [(η^6^‐*p*‐cymene)(Mes*P)Os(^Me^I*i*Pr)] (557.6 ppm) containing the NHC ligand 1,3‐diisopropyl‐4,5‐dimethylimidazolin‐2‐ylidene (^Me^I*i*Pr).^[10]^ In comparison with the PMe_3_ complex **4 b**, the resonance in **5** is significantly more shielded, in analogy to the corresponding phosphinidene complexes of type **I** (L=PR_3_, NHC), which has been attributed to the stronger σ‐donor capacity of the NHC.[^9^, ^10^] The molecular structure of **5** was determined by X‐ray diffraction analysis (Figure 2), revealing similar structural features as discussed for **4 b** and an *E* configuration of the two NHC units with Os‐P‐C1 and P‐Os‐C4 angles of 117.84(6)° and 80.89(5)°. The Os−P bond length is 2.2119(5) Å, which is slightly longer than 2.2005(9) Å in **4 b**, but virtually identical with 2.2195(7) Å reported for [(η^6^‐*p*‐cymene)(Mes*P)Os(PPh_3_)].^[6]^ The Os‐P‐C1‐N1 torsion angle of 141.65(13)° indicates an oblique orientation of the IDipp unit, whereas the carbene ligand ^Me^IMe is oriented in a more horizontal fashion with P‐Os‐C4‐N3=75.55(17)°.

### Synthesis and characterization of cationic iridium (NHC)P complexes

Chloride abstraction with sodium tetrakis[3,5‐bis(trifluoromethyl)phenyl]borate (NaBAr^F^) in fluorobenzene at room temperature was also performed with the iridium complex [(η^5^‐C_5_Me_5_){(IDipp)P}IrCl] (**3 b**) in the presence of trimethylphosphine (PMe_3_) and 1,3,4,5‐tetramethylimidazolin‐2‐ylidene (^Me^IMe), affording the complexes [(η^5^‐C_5_Me_5_){(IDipp)P}IrL][BAr^F^] (**6**, L=PMe_3_; **7**, L=^Me^IMe) as brown solids in moderate yield (ca. 55 %, Scheme 2). In a similar fashion as described for the Ru and Os complexes **4**, the PMe_3_ complex **6** gives rise to a doublet in the ^1^H NMR spectrum (in [D_8_]THF) at 1.50 ppm (^2^ *J*_PH_=11.5 Hz), while two doublets can be found in the ^31^P NMR spectrum at 454.9 and −33.1 ppm (^2^ *J*_PP_=106.7 Hz), which can be assigned to the (IDipp)P and PMe_3_ phosphorus nuclei. The low‐field (IDipp)P resonance is deshielded compared to the starting material **3 b** (353.7 ppm), but shielded with respect to related iridium complexes of type **II**, e.g., 686.6/24.8 ppm (^2^ *J*_PP_=102 Hz) in (*E*)‐[(η^5^‐C_5_Me_5_)(Mes*P)Ir(PPh_3_)].^[5]^ The corresponding trimethylphosphine complex [(η^5^‐C_5_Me_5_)(Mes*P)Ir(PMe_3_)] was isolated as a mixture of *E* and *Z* isomers, which afforded 629.0/‐37.3 ppm (^2^ *J*_PP_=84 Hz) for the *E* form and 726.6/‐41.7 ppm (^2^ *J*_PP_=17.6 Hz) for the *Z* form. Accordingly, the large ^2^ *J*_PP_ coupling constant of 106.7 Hz found for **6** allows to unequivocally assign an *E* configuration.[^5^, ^7^] In contrast to the PMe_3_ complex **6**, the ^Me^IMe complex **7** was isolated as a mixture of *E* and *Z* isomers as indicated by the observation of two singlets in the ^31^P NMR spectrum at 399.0 ppm (*E*) and 483.8 ppm (*Z*) in about 76:24 ratio. This assignment is in agreement with the trend established experimentally and theoretically for the *E* and *Z* forms of related group 9 complexes with terminal phosphinidene ligands, with the *Z* form generally showing the more deshielded ^31^P NMR chemical shift.^[7]^ In contrast, only the *E* isomer was observed for [(η^5^‐C_5_Me_5_)(Mes*P)Ir(^Me^I*i*Pr)] (*δ*=560.0 ppm), which contains the sterically more congested carbene 1,3‐diisopropyl‐4,5‐dimethylimidazolin‐2‐ylidene (^Me^I*i*Pr).[^8^, ^10^]

**Figure 3:**
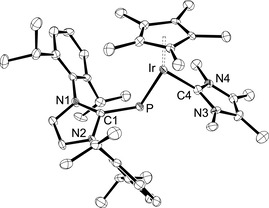
ORTEP diagram of one of the two independent cations in **7** with thermal displacement parameters drawn at the 50 % probability level. Hydrogen atoms and the BAr^F^ counterion were omitted for clarity. Selected bond lengths and angles are assembled in Table 1.

Single crystals of **6** and **7** suitable for X‐ray diffraction analysis were obtained from fluorobenzene solution. In case of the PMe_3_ complex **6**, severe disorder of the BAr^F^ counterion prevented sufficient refinement, and only the connectivity and the presence of the *E* isomer in the solid state could be confirmed (see Figure S66 in the Supporting Information). Complex **7** crystallized in the monoclinic space group *P*2_1_ with two independent molecules in the asymmetric unit; and cation 1 is presented in Figure 3. Both cations show an *E* configuration with Ir‐P1‐C1 and P‐Ir‐C4 angles of 120.3(4)/121.1(3)° and 82.6(3)/81.0(3)° (molecule 1/molecule 2). The Ir−P bond lengths are 2.193(3)/2.205(2) Å, which is almost identical with 2.1966(8) Å in the starting material **3 b** ^[15]^ and also with 2.1959(5) Å in the neutral phosphinidene‐congener [(η^5^‐C_5_Me_5_)(Mes*P)Ir(^Me^I*i*Pr)].^[8]^ However, the Ir−C_carbene_ distance of 2.0278(19) Å in the latter complex is slightly shorter compared to 2.050(12)/2.050(9) Å (Ir−C4) in **7**. Similar to the osmium NHC complex **5**, the torsion angles Ir‐P‐C1‐N2 and P‐Ir‐C4‐N3 of 118.2(8)/122.0(7)° and 90.2(9)/89.3(9) indicate oblique and horizontal conformations of the IDipp and ^Me^IMe units, respectively. Moreover, the IDipp moiety is significantly tilted, displacing the phosphorus atom by 0.517(18)/0.521(17) Å from the imidazole plane, which could be ascribed to steric congestion with the C_5_Me_5_ ligand. Similar distortions are observed in the Ru and Os complexes **4** and **5**, albeit to a smaller extend (ca. 0.3 Å). Finally, it could be noted that the *E* configuration of the two NHC ligands in complexes **5** and **7** is reminiscent of the *trans*‐bent geometry established for heteroleptic dicarbene‐di‐element species such as [(IDipp)PE(IMes)]^*n*^ (E = P, As; *n* = 0, +1, +2; IMes = 2,4,6‐trimethylimidazolin‐2‐ylidene),^[24]^ and in this vein, the complexes **5** and **7** might also be *formally* regarded as carbene‐stabilized terminal phosphido complexes of the type [(η^6^‐*p*‐cymene)OsP]^+^ and [(η^5^‐C_5_Me_5_)IrP]^+^.

**Scheme 4:**
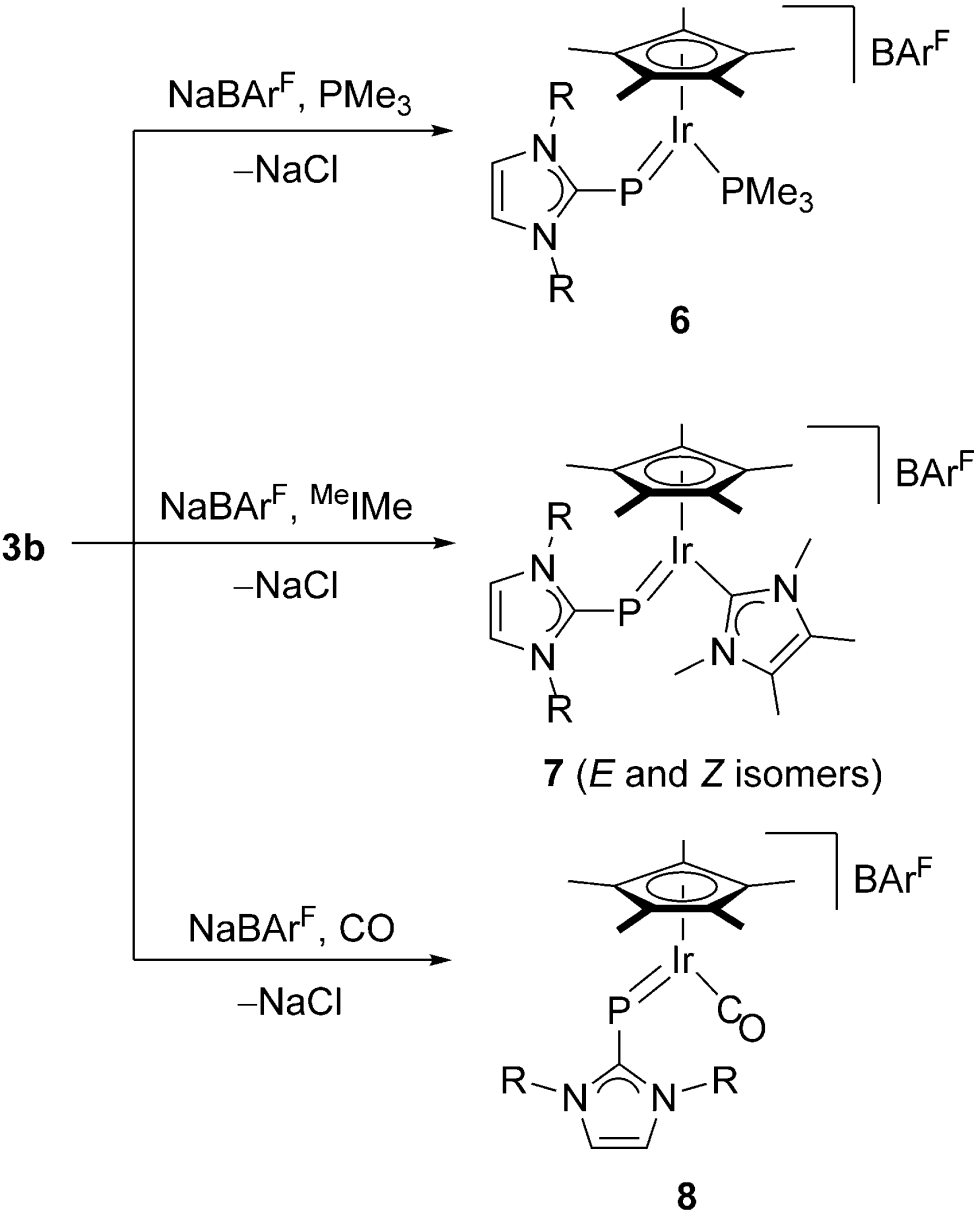
Synthesis of cationic iridium (NHC)P complexes; R=Dipp=2,6‐diisopropylphenyl.

**Figure 4:**
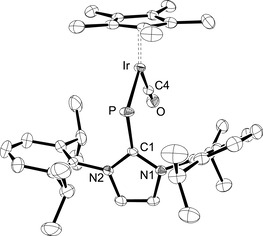
ORTEP diagram of the cationic complex in **8** with thermal displacement parameters drawn at the 50 % probability level. Hydrogen atoms and the BAr^F^ counterion were omitted for clarity. Selected bond lengths and angles are assembled in Table 1.

The carbonyl complex [(η^5^‐C_5_Me_5_){(IDipp)P}Ir(CO)][ BAr^F^] (**8**) was prepared by leading a stream of carbon monoxide through the reaction mixture containing **3 b** and NaBAr^F^ in fluorobenzene solution (Scheme 4). The dark blue color of **3 b** vanished and turned dark red within a few minutes. **8** was isolated in analytically pure form as a purple‐red solid in 51 % yield. In comparison with complexes **6** and **7**, the significantly more deshielded ^31^P NMR chemical shift at 596.9 ppm suggests a *Z* configuration as also observed for the related neutral carbonyl‐phosphinidene complex [(η^5^‐C_5_Me_5_)(Mes*P)Ir(CO)] (*δ*=804.6 ppm). The IR spectrum of **8** exhibits a strong absorption at 1998 cm^−1^, which is larger than the value reported for [(η^5^‐C_5_Me_5_)(Mes*P)Ir(CO)] (1968 cm^−1^).^[5]^ This difference could be ascribed tentatively to a stronger π‐accepting ability of the (IDipp)P in comparison with the Mes*P ligand; however, the cationic and neutral nature of the two complexes must also be considered. The *Z* configuration of **8** was also confirmed for the solid state by X‐ray diffraction analysis, and the molecular structure of the cation in **8** is shown in Figure 4. It displays a bent (IDipp)P ligand and the typical two‐legged piano‐stool geometry with Ir‐P1‐C1 and P‐Ir‐C4 angles of 113.73(11)° and 98.54(11)°, which is similar to the structural features of **3 b**.^[15]^ The Ir−P bond and Ir−C4 bond lengths of 2.1905(10) and 1.870(4) Å are both longer compared to 2.1783(8) and 1.849(3) Å in [(η^5^‐C_5_Me_5_)(Mes*P)Ir(CO)], which suggests slightly higher bond orders for the metal‐element bonds in the neutral phosphinidene system. The imidazole ring subtends an interplanar angle of 85.86(12)° with the plane containing the atoms C1‐P‐Ir‐C4‐O, revealing an orthogonal orientation.

### Chemical bonding analysis

To evaluate the bonding situation in the complexes **4**–**8**, the structures of the cationic osmium and iridium derivatives [(η^6^‐*p*‐cymene){(IDipp)P}OsL]^+^ and [(η^5^‐C_5_Me_5_){(IDipp)P}IrL]^+^ (L=CO, PMe_3_, ^Me^IMe) were optimized in their respective *E* and *Z* forms by applying the density functional theory (DFT) method B97‐D, followed by natural bonding orbital (NBO) analysis. For comparison, the neutral phosphinidene complexes [(η^6^‐*p*‐cymene)(Mes*P)Os(PMe_3_)] and [(η^5^‐C_5_Me_5_)(Mes*P)Ir(PMe_3_)] were also included in this study as representatives of complexes of type **I** and **II**. The computational details and selected contour plots of selected NBOs are given in the Supporting Information (Tables S7–S9). The calculated structural parameters are very similar to those found experimentally in the X‐ray crystal structures, if available (cf. Tables 1 and 2). In agreement with exclusive observation of *E* isomers in solution and in the solid state in case of the osmium derivatives **4 b** (L=PMe_3_) and **5** (L=^Me^IMe), the *E* configuration is clearly favored thermodynamically by 8.8 and 7.0 kcal mol^−1^, respectively (Entries 2 and 3, Table 2). In contrast, the *Z* configuration is more stable (Δ*H*_298K_=6.5 kcal mol^−1^) for the elusive carbonyl complex (L=CO, Entry 4). For the iridium trimethylphosphine (L=PMe_3_) and NHC (L=^Me^IMe) complexes, however, very similar energies were computed for the *E* and *Z* forms, with the latter being slightly favored by 1.9 and 1.6 kcal mol^−1^ (Entries 6 and 7). In principle, this trend is in line with the observation of an *E*/*Z* mixture in solution for the NHC complex **7**, whereas only the *E* form was observed for the PMe_3_ congener **6**. Strong stabilization by 13.3 kcal mol^−1^ of the *Z* form is again found for the carbonyl species (L=CO, Entry 8), in agreement with the experimental data established for **8**.

**Table 2:** Relative enthalpies (Δ*H*_298K_), Natural Bond Orbital (NBO) charges (*q*) and Wiberg Bond Indices (WBI) of phosphinidene and (NHC)P complexes.

Entry	Complex		Δ*H*_298K_ [kcal/mol]	*q*(P)	*q*(M)	WBI (P−C)	WBI (M−P)	*d*(M−P) [Å]
1	[(η^6^‐*p‐*cymene)(Mes*P)Os(PMe_3_)] (**I**)	*E*	0	0.39	−0.85	0.89	1.55	2.223
		*Z*	5.2	0.45	−0.89	0.92	1.62	2.224
2	[(η^6^‐*p‐*cymene){(IDipp)P}Os(PMe_3_)]^+^ (in **4 b**)	*E*	0	0.32	−0.79	0.97	1.53	2.209
		*Z*	8.8	0.39	−0.83	1.01	1.57	2.213
3	[(η^6^‐*p‐*cymene){(IDipp)P}Os(^Me^IMe )]^+^ (in **5**)	*E*	0	0.33	−0.58	0.98	1.50	2.219
		*Z*	7.0	0.33	−0.58	1.01	1.55	2.215
4	[(η^6^‐*p‐*cymene){(IDipp)P}Os(CO)]^+^	*E*	6.5	0.47	−0.70	0.96	1.50	2.219
		*Z*	0	0.47	−0.70	0.95	1.54	2.232
5	[(η^5^‐C_5_Me_5_)(Mes*P)Ir(PMe_3_)] (**II**)	*E*	0	0.36	−0,61	0.91	1.40	2.215
		*Z*	0.2	0.46	−0.68	0.93	1.48	2.191
6	[(η^5^‐C_5_Me_5_){(IDipp)P}Ir(PMe_3_)]^+^ (in **6**)	*E*	1.9	0.25	−0.54	0.98	1.36	2.207
		*Z*	0	0.32	−0.60	0.99	1.40	2.209
7	[(η^5^‐C_5_Me_5_){(IDipp)P}Ir(^Me^IMe )]^+^ (in **7**)	*E*	1.6	0.26	−0.37	0.99	1.37	2.207
		*Z*	0	0.27	−0.39	1.04	1.40	2.200
8	[(η^5^‐C_5_Me_5_){(IDipp)P}Ir(CO)]^+^ (in **8**)	*E*	13.3	0.38	−0.45	0.97	1.33	2.220
		*Z*	0	0.41	−0.46	0.96	1.35	2.233

Comparison of the NBO charges in the neutral and cationic PMe_3_ osmium and iridium complexes (entries 1/2 and 5/6) reveals a very similar charge distribution, albeit with a slightly higher polarization of the metal–phosphorus bonds calculated for the authentic phosphinidene complexes of type **I** and **II**. Furthermore, the similarity of the Wiberg bond indices (WBI) indicate equal bond orders, with just a marginal decrease found for the cationic (NHC)P systems. It should be noted, however, that this trend is not reflected by the metal‐phosphorus bond lengths, which are virtually identical in the respective osmium and iridium pairs. Substitution of the PMe_3_ ligand by ^Me^IMe and CO (Entries 3/4 and 7/8) does not reveal a clear trend and produces similar values (Table 2). Overall, this bonding situation is best described by the mesomeric structure **A** (Scheme 1), with the (NHC)P unit acting as a cationic phosphinidene ligand with highly covalent σ‐ and π‐contributions. Accordingly, the metal‐phosphorus double bond can be described in terms of an interaction between a triplet (NHC)P cation and a triplet metal complex fragment in analogy with nucleophilic phosphinidene complexes such as **I** and **II**.[^3^, ^25^] In fact, [(IDipp)P]^+^ has a triplet ground state, which is 8.4 kcal mol^−1^ (Δ*H*_298K_) below the singlet state, while the neutral phosphinidene Mes*P has a similar triplet‐singlet gap of 7.9 kcal mol^−1^ (see the Supporting Information). In comparison, the triplet ground state of the parent phosphinidene (HP) has been predicted to be 20–28 kcal mol^−1^ below the singlet state.^[26]^ In this context, it is noteworthy that, on the other hand, transient or even isolable (phosphino)phosphinidenes have a singlet ground state, which is about 20 kcal mol^−1^ below the triplet state.^[27]^

**Figure 5:**
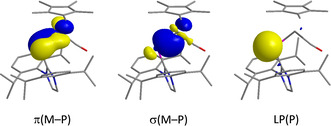
Selected natural bond orbitals (NBOs) of [(η^5^‐C_5_Me_5_){(IDipp)P}Ir(CO)]^+^ (as in **8**) associated with the iridium‐phosphorus π‐ and σ‐bonds and with the phosphorus lone pair (LP).

The high degree of covalency of the metal–phosphorus double bond is evidenced by almost equal contributions of the metal and phosphorus atoms to the natural bond orbitals (NBOs) associated with the M−P π‐ and σ‐bonds. Furthermore, the expected high 3s character of the phosphorus lone pair is also confirmed (see the Supporting Information for the composition and the contour plots of relevant NBOs). As an illustrative example, the NBOs of the iridium complex [(η^5^‐C_5_Me_5_){(IDipp)P}Ir(CO)]^+^ (as in **8**) are shown in Figure 5.

### Trapping of a 16‐electron intermediate

**Scheme 5:**
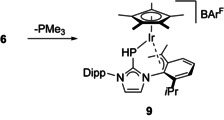
Formation of the η^3^‐benzyl complex **9**; Dipp=2,6‐diisopropylphenyl.

**Figure 6:**
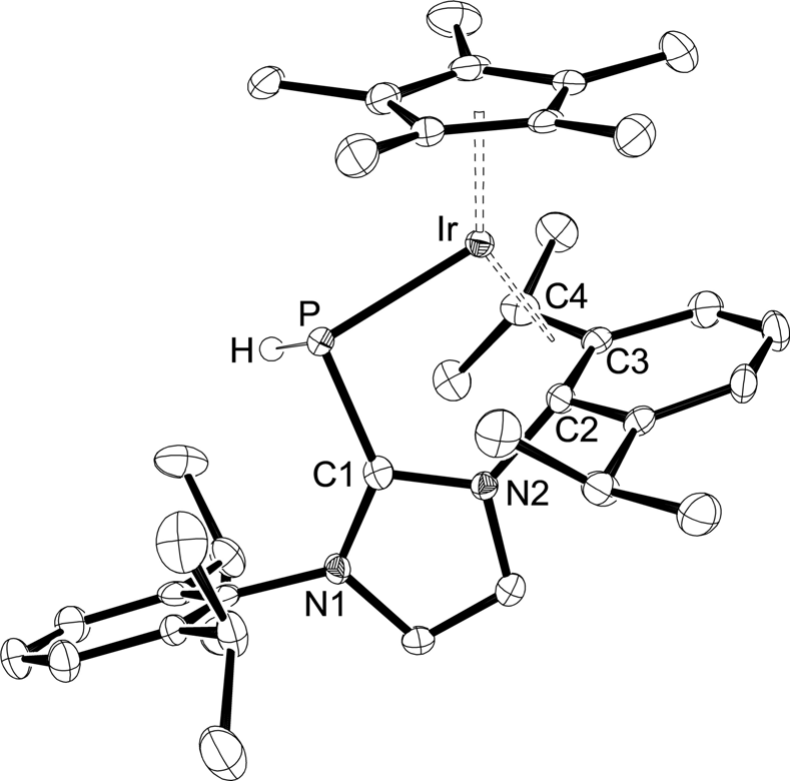
ORTEP diagram of one of the two independent cations in **9**⋅0.5 C_6_H_6_ with thermal displacement parameters drawn at the 50 % probability level. The PH hydrogen atom has been placed and refined in a position adjacent to the η^3^‐benzyl moiety. All other hydrogen atoms, the solvent molecule and the BAr^F^ counterion were omitted for clarity. Selected bond lengths and angles are assembled in Table 3.

The reactivity of phosphinidene complexes of type **I** and **II** has been explained by involvement of putative 16‐electron intermediates [(η^6^‐Ar)M(PMes*)] (M=Ru, Os) and [(η^5^‐C_5_Me_5_)M(PMes*)] (M=Rh, Ir),^[4]^ and convincing evidence has been provided by ^31^P NMR spectroscopy for the formation [(η^5^‐C_5_Me_5_)Ir(PDmp)] containing the bulky 2,6‐dimesitylphenyl (Dmp) substituent.^[28]^ In our hands, attempts to generate and characterize 16‐electron intermediates by chloride abstraction from **2** and **3** proved unsuccessful. However, the potential reactivity of these 16‐electron intermediates, which have been captured by adding ligands such as PMe_3_, ^Me^IMe and CO as described above, can be demonstrated by repeated isolation of yellow single crystals from solutions containing the PMe_3_ complex **6** (Scheme 5), unfortunately only as an inseparable mixture with the brown rods of **6** (see the Supporting Information). X‐ray diffraction analysis revealed the formation of complex **9** with a C−H activated isopropyl group. **9**⋅0.5 C_6_H_6_ crystallized in the monoclinic space group *P*2_1_/*c* with two independent molecules in the asymmetric unit; and cation 1 is presented in Figure 6. The structural parameters of both cations are almost identical, and the following discussion is restricted to cation 1. One isopropyl group has undergone C−H activation and deprotonation at the methine position, affording a symmetric η^3^‐benzyl coordination mode with Ir‐C distances of 2.194(5), 2.163(4) and 2.200(5) Å; these bond lengths are in the same range as the Ir−C distances to the η^5^‐C_5_Me_5_ ligand, *viz*. 2.192(5)–2.300(4) Å. The Ir−P bond length of 2.3360(12) Å indicates protonation of the phosphorus atom, since it is significantly longer compared to 2.1966(8) Å in **3 b**,^[15]^ 2.193(3)/2.205(2) Å in **7** and 2.1905(10) Å in **8** (Table 1), but almost identical with 2.3460(6) Å in [(η^5^‐C_5_Me_5_)IrCl_2_{PH(IDipp)}].^[15]^ However, the PH hydrogen atom could not be located unequivocally, and therefore the structure of **9** was optimized by DFT calculations (B97‐D) with the hydrogen atom located either adjacent or opposite to the η^3^‐benzyl moiety. Both structures are very similar in energy (Δ*H*_298K_=0.3 kcal mol^−1^, suggesting that the hydrogen atom might be disordered over these two positions. The experimental and calculated parameters are in good agreement, although the calculated bond lengths tend to be consistently longer (Table 3).

**Table 3:** Experimental and calculated structural parameters of **9**.

Bond lengths [Å] and angles [°]	exp.^[a]^	calc.^[b]^	calc.^[c]^
Ir−P	2.3360(12)/2.3329(12)	2.370	2.378
Ir−C2	2.194(5)/2.188(4)	2.240	2.234
Ir−C3	2.163(4)/2.157(4)	2.195	2.199
Ir−C4	2.200(5)/2.211(4)	2.225	2.240
P−C1	1.802(5)/1.794(5)	1.833	1.836
Ir‐P‐C1	97.95(14)/97.62(14)	97.07	97.02

[a] For two crystallographically independent molecules. [b] Isomer with the PH hydrogen atom adjacent to the η^3^‐benzyl moiety. [c] Isomer with the PH hydrogen atom opposite to the η^3^‐benzyl moiety.

Complex **9** should have formed by dissociation of PMe_3_ from complex **6**, and the resulting 16‐electron species has probably undergone C−H activation along the iridium‐phosphorus bond. To the best of our knowledge, the resulting η^3^‐benzyl coordination mode is rare,^[29]^ although C−H activation represents a common pathway of decomposition of NHC complexes containing carbenes such as IDipp and IMes.^[30]^ Unfortunately, attempts to synthesize compound **9** by chloride abstraction from **3 b** and to characterize it spectroscopically have so far been unsuccessful, and at this stage, the isolation of **9** only allows a glimpse into the potentially interesting reactivity of cationic 16‐electron complexes of the type [(η^6^‐Ar)M{P(IDipp)}]^+^ (M=Ru, Os) and [(η^5^‐C_5_Me_5_)M{P(IDipp)}]^+^ (M=Rh, Ir).

## Conclusions

The N‐heterocyclic carbene‐phosphinidene complexes presented in this study can be conceived as new and true members of the class of nucleophilic phosphinidene complexes according to their spectroscopic, structural and electronic properties. Substitution of the aryl moiety in authentic neutral arylphosphinidene complexes of the type [ArP=ML_*n*_] by an N‐heterocyclic carbene affords the corresponding cationic complexes [(NHC)P=ML_*n*_]^+^ with equally short and covalent metal‐phosphorus bonds. The chloride abstraction reactions described herein probably generate reactive 16‐electron species of the type [(η^6^‐*p*‐cymene)M{P(IDipp)}]^+^ (M=Ru, Os) and [(η^5^‐C_5_Me_5_)Ir{P(IDipp)}]^+^ which can be trapped by addition of suitable ligands such as phosphines, NHCs or carbon monoxide. The potential reactivity of these species will be further studied, and the reactions with unsaturated substrates such as alkenes, alkynes, carbon dioxide etc. might uncover interesting cycloaddition reactivity along the metal phosphorus double bond. Furthermore, their potential role as homogeneous catalysts for hydroelementation reactions will be investigated.

## Experimental Section

### General considerations

All manipulations were carried out under dry argon atmosphere using standard Schlenk techniques or argon‐filled glove boxes. Solvents were dried using an MBraun solvent purification system. The starting materials (IDipp)PSiMe_3_ (**1**),^[11]^ [(η^6^‐*p*‐cymene){(IDipp)P}MCl] (**2 a**, M=Ru; **2 b**, M=Os),^[15]^ [(η^5^‐C_5_Me_5_){(IDipp)P}IrCl],^[15]^ 1,3,4,5‐tetramethylimidazolin‐2‐ylidene (^Me^IMe)^[31]^ and sodium tetrakis[(3,5‐trifluoromethyl)phenyl]borate NaBAr^F[32]^ were prepared according to the previously published procedures. ^1^H, ^13^C, ^11^B, ^19^F and ^31^P NMR spectra were recorded on Bruker AV 300 (300 MHz) and Bruker DRX 400 (400 MHz) devices. The chemical shifts are given in parts per million (δ; ppm) relative to residual solvent peaks. Coupling constants (*J*) are reported in Hertz (Hz), and splitting patterns are indicated as s (singlet), d (doublet), t (triplet), m (multiplet), sept (septet) and br (broad). All spectra were measured at room temperature unless otherwise stated. IR spectra were recorded on a Bruker VERTEX 70 FTIR spectrometer equipped with a Pike Technologies MIRacle attenuated total reflectance (ATR) unit, and mass spectra on Finnigan MAT 95 (EI) and Finnigan MAT 95 XL (ESI) systems, respectively. Elemental analyses were carried out on a Vario Micro Cube System. X‐ray crystallographic and computational details are described in the Supporting Information.

Deposition Numbers 1972563, 1972564, 1972565, 1972566, 1972567 and 2012410 contain the supplementary crystallographic data for this paper. These data are provided free of charge by the joint Cambridge Crystallographic Data Centre and Fachinformationszentrum Karlsruhe Access Structures service www.ccdc.cam.ac.uk/structures.

### Synthesis and characterization

**Synthesis of [(η^6^‐*p*‐cymene){(IDipp)P}Ru(PMe_3_)][BAr^F^] (4 a)**: A Schlenk tube was charged with [(η^6^‐*p*‐cymene){(IDipp)P}RuCl] (**2 a**, 0.060 g, 0.087 mmol) and PMe_3_ (0.007 g, 0.091 mmol) in fluorobenzene (10 mL). NaBAr^F^ (0.077 g, 0.087 mmol) was added as a solid at room temperature while stirring the reaction mixture. The resulting solution turned dark purple, and stirring was continued at room temperature for 1 h, followed by filtration and evaporation of the solvent. The residue was thoroughly washed with *n*‐hexane and dried under vacuum to obtain **4 a** as a purple solid. Yield: 0.118 g (85.5 %).^1^H NMR ([D_8_]THF, 300.1 MHz): *δ*=8.00 (s, 2 H, *H*C_2_N_2_), 7.80–7.77 (br, 8 H, *H*‐BAr^F^), 7.58.7.56(br, 4 H, *H*‐BAr^F^), 7.55–7.51 (m, 2 H, Ar‐H), 7.43–7.40 (m, 4 H, *H*‐Ph), 5.15 (br, 2 H, cymene‐C*H*), 5.07 (br, 2 H, cymene‐C*H*), 2.71 (sept, 4 H, ^3^ *J*_HH_=6.7 Hz, C*H*(CH_3_)_2_), 2.47 (sept, 1 H, ^3^ *J*_HH_=6.8 Hz, cymene‐*CH*(CH_3_)_2_), 1.78 (s, 3 H, cymene‐C*H_3_*), 1.42 (d, 9 H, ^2^ *J*_PH_=9.5 Hz, P(C*H_3_*)_3_), 1.31 (d, 12 H, ^3^ *J*_HH_=6.8 Hz, CH(C*H_3_*)_2_), 1.22 (d, broad, 12 H, ^3^ *J*_HH_=6.8 Hz, CH(C*H_3_*)_2_), 0.92 (d, 6 H, ^3^ *J*_HH_=6.7 Hz, cymene‐CH(C*H_3_*)_2)_  ppm. ^13^C NMR ([D_8_]THF, 75.47 MHz): P‐*C_carbene_* not visible), 163.4 (q, ^1^ *J*_BC_=48.8 Hz, B‐C(Ar^F^)), 146.9 (N*C*(Dipp)), 135.3 (*o‐C*(BAr^F^), 133.8 (*o‐C*(Dipp), 132.6 (*p‐C*(Dipp)),130.6 (qq, ^2^ *J*_CF_=32 Hz, ^3^ *J*_BC_=2.9 Hz, *m‐C*‐BAr^F^), 127.9 (H_2_ *C_2_*N_2_), 126.3 (*m‐C*(Dipp)), 124.4 (q, ^1^ *J*_CF_=272 Hz, *C*F_3_‐BAr^F^), 118.4 (sept, ^3^ *J*_FC_=4.3 Hz, *p*‐C(BAr^F^)), 105.1 (cymene‐*C*H), 93.0 (cymene‐*C*H), 87.3 (cymene‐*C*H), 84.5 (cymene‐*C*H), 33.2 (cymene‐*C*H(CH_3_)_2_), 30.5 (*C*H(*C*H_3_)_2_), 26.1 (CH(*C*H_3_)_2_), 25.0 (CH(*C*H_3_)_2_), 23.6 (cymene‐CH(*C*H_3_)_2_), 22.1 (dd, *J*_PC_=31.3 Hz, *J*_PC_=8.5 Hz, P‐*C*H_3_) and 21.1 (cymene‐*C*H_3)_  ppm. ^31^P NMR ([D_8_]THF, 121.4 MHz); *δ*=−15.11 (d, ^2^ *J*_PP_=41.32 Hz, *P*Me_3_) and 592.89 (d, ^2^ *J*_PP_=41.32 Hz, IDipp=*P*‐Ru) ppm. ^11^B NMR ([D_8_]THF, 96.3 MHz): *δ*=−5.98 ppm. HRMS (ESI‐positive ion mode, CH_3_CN/Toluene): *m*/*z*: calcd for the cationic moiety C_40_H_59_RuN_2_P_2_: 731.32025; found: 731.31937. Anal. calcd (%) for C_72_H_71_BF_24_N_2_P_2_Ru (1594.38 g mol^−1^): C 54.19, H 4.48 and N 1.75; Found: C 54.28, H 4.22 and N 1.82.

**Synthesis of [(η^6^‐*p*‐cymene){(IDipp)P}Os(PMe_3_)][BAr^F^] (4 b)**: NaBAr^F^ (0.057 g, 0.064 mmol) was added as a solid to a Schlenk tube containing [(η^6^‐*p*‐cymene){(IDipp)P}OsCl] (**2 b**, 0.050 g, 0.064 mmol) and PMe_3_ (0.012 g, 2.5 equiv, 0.160 mmol) in fluorobenzene (10 mL) under vigorous stirring. The resulting reaction mixture was stirred at room temperature for 4 h, then filtered and evaporated. The residue was thoroughly washed with *n*‐hexane and dried under vacuum to obtain **4 b** as a purple solid. Yield: 0.081 g (75 %). ^1^H NMR ([D_8_]THF, 600.1 MHz): *δ*=7.91 (s, 2 H, *H*C_2_N_2_), 7.80–7.78 (br, 8 H, *H*‐BAr^F^), 7.60 −7.56 (br, 4 H, *H*‐BAr^F^), 7.55 −7.51 (m, 2 H, Ar‐H), 7.44–7.41 (m, 4 H, *H*‐Ph), 5.31 (br, 4 H, cymene‐C*H*), 2.70 (sept, 4 H, ^3^ *J*_HH_=6.4 Hz, C*H*(CH_3_)_2_), 2.56 (sept, 1 H, ^3^ *J*_HH_=6.8 Hz, cymene‐*CH*(CH_3_)_2_), 2.00 (s, 3 H, cymene‐C*H_3_*), 1.53 (d, 9 H, ^2^ *J*_PH_=10.7 Hz, P(C*H_3_*)_3_), 1.35 (d, 12 H, ^3^ *J*_HH_=7.3 Hz, CH(C*H_3_*)_2_), 1.21 (d, broad, 12 H, ^3^ *J*_HH_=6.9 Hz, CH(C*H_3_*)_2_), 1.01 (d, 6 H, ^3^ *J*_HH_=7.0 Hz, cymene‐CH(C*H_3_*)_2)_  ppm. ^13^C NMR ([D_8_]THF, 150.9 MHz): *δ*=184.6 (dd, *J*_PC_=175 Hz, *J*_PC_=28 Hz, P‐*C_carbene_*), 163.0 (q, ^1^ *J*_BC_=49.5 Hz, B‐*C*(Ar^F^)), 146.4 (N*C*(Dipp)), 135.7 (*o‐C*(BAr^F^), 135.0 (*o‐C*(Dipp), 132.1 (*p‐C*(Dipp)), 130.2 (qq, ^2^ *J*_CF_=32 Hz, ^3^ *J*_BC_=3.1 Hz, *m‐C*‐BAr^F^), 127.4 (H_2_ *C_2_*N_2_), 125.9 (*m‐C*(Dipp)), 125.7 (q, ^1^ *J*_CF_=272 Hz, *C*F_3_‐BAr^F^), 118.4 (sept, ^3^ *J*_FC_=4.0 Hz, *p*‐C(BAr^F^)), 96.0 (cymene‐*C*H), 83.9 (cymene‐*C*H), 81.4 (cymene‐*C*H), 78.6 (cymene‐*C*H), 33.4 (cymene‐*C*H(*C*H_3_)_2_), 30.0 (*C*H(*C*H_3_)_2_), 25.7 (CH(*C*H_3_)_2_), 24.8 (cymene‐CH(*C*H_3_)_2_), 23.3 (CH(*C*H_3_)_2_), 22.8 (dd, *J*_PC_=38 Hz, *J*_PC_=5.4 Hz, P‐*C*H_3_), 21.6 (cymene‐*C*H_3)_  ppm. ^31^P NMR ([D_8_]THF, 121.4 MHz); *δ*=−37.05 (d, ^2^ *J*_PP_=99.0 Hz, *P*Me_3_) and 438.90 (d, ^2^ *J*_PP_=99.0 Hz, (IDipp)*P*‐Os) ppm. ^11^B NMR ([D_8_]THF, 96.2 MHz): *δ*=−6.15 ppm. HRMS (ESI‐positive ion mode, CH_3_CN/Toluene): *m*/*z*: calcd for the cationic moiety C_40_H_59_OsN_2_P_2_: 821.3709; found: 821.3764. Anal. calcd (%) for C_72_H_71_BF_24_OsN_2_P_2_ (1684.4408 g mol^−1^): C 51.28, H 4.24 and N 1.66; Found: C 50.28, H 4.11 and N 1.91.

**Synthesis of [(η^6^** ***‐p*** **‐cymene){(IDipp)P}Os(^Me^IMe)][BAr^F^] (5)**: NaBAr^F^ (0.068 g, 0.077 mmol) was added to a Schlenk tube containing [(η^6^‐*p*‐cymene){(IDipp)P}OsCl] (**2 b**, 0.060 g, 0.077 mmol) and 1,3,4,5‐tetramethylimidazolin‐2‐ylidene (0.010 g, 0.080 mmol) in fluorobenzene (10 mL). The resulting mixture was stirred at room temperature for 4 h, then filtered and evaporated. The residue was thoroughly washed with *n*‐hexane and dried under vacuum to obtain **5** as brown‐red solid. Yield: 0.094 g (70 %). ^1^H NMR ([D_8_]THF, 600.1 MHz): *δ*=7.80–7.78 (br, 8 H, *H*‐BAr^F^), 7.77 (s, 2 H, *H*C_2_N_2_), 7.57 (br, 4 H, *H*‐BAr^F^), 7.53–7.51 (m, 2 H, Ar‐*H*), 7.40–7.38 (m, 4 H, Ar‐*H*), 5.11 (d, 2 H, ^3^ *J*_HH_=5.9 Hz, cymene‐*C*H), 5.01 (d, 2 H, ^3^ *J*_HH_=5.9 Hz, cymene‐*C*H), 3.24 (s, 6 H, ^Me^IMe, *N*‐C*H_3_*), 2.74 (sept, 4 H, ^3^ *J*_HH_=6.7 Hz, C*H*(CH_3_)_2_), 2.47 (sept, 1 H, ^3^ *J*_HH_=6.9 Hz, cymene‐C*H*(CH_3_)_2_), 2.08 (s, 6 H, ^Me^IMe, C*H_3_*‐C_2_N_2_), 2.04 (s, 3 H, cymene‐C*H_3_*), 1.33 (d, 12 H, ^3^ *J*_HH_=6.8 Hz, CH(C*H_3_*)_2_), 1.17 (d, 12 H, ^3^ *J*_HH_=6.8 Hz, CH(C*H_3_*)_2_), 1.02 (d, 6 H; ^3^ *J*_HH_=6.9 Hz, cymene‐CH(C*H_3_*)_2)_  ppm. ^13^C NMR ([D_8_]THF_,_ 150.9 MHz). *δ*=166. 0 (^Me^IMe), 163.0 (q, ^1^ *J*_BC_=50.6 Hz, *C*‐B, B(Ar^F^)), 146.5 (N*C*(Dipp)), 135.7 (*o‐C*(BAr^F^), 133.5 (*o‐C*(Dipp)), 131.7 (*p‐C*(Dipp)), 130.2 (qq, ^2^ *J*_CF_=31.7 Hz, ^3^ *J*_BC_=2.8 Hz, *m‐C*‐BAr^F^), 125.9 (^Me^IMe), 125.3 (*m‐C*(Dipp)), 125.5 (q, ^1^ *J*_CF_=272.2 Hz, *C*F_3_‐BAr^F^), 118.1 (sept, ^3^ *J*_FC_=4.0 Hz, *p*‐C(BAr^F^)), 116.1 (d, ^3^ *J*_PC_=22.5 Hz, H‐*C*_2_N_2_), 96.1 (cymene‐*C*H), 83.9 (cymene‐*C*H), 76.8 (cymene‐*C*H), 74.3 (cymene‐*C*H), 39.0 (^Me^IMe), 33.3 (*C*H(CH_3_)_2_), 30.3 (cymene‐*C*H(CH_3_)_2_) 24.8 (CH(*C*H_3_)_2_), 24.4 (cymene‐CH(*C*H_3_)_2_) 23.7 (CH(*C*H_3_)_2_, 23.2 (cymene‐*C*H_3_), 9.3 (^Me^IMe) ppm. ^31^P NMR ([D_8_]THF, 162.1 MHz): *δ*=394.25 (s) ppm. ^11^B NMR ([D_8_]THF, 96.3 MHz); *δ*=−5.99 (s) ppm. ^19^F NMR ([D_8_]THF, 282.4 MHz): *δ*=−62.45 (s) ppm. HRMS (positive ion mode, CH_3_CN): *m*/*z*: calcd for cationic moiety C_44_H_62_OsN_4_P: 869.4324; found: 869.4322. Anal. calcd (%) for C_76_H_74_BF_24_N_4_OsP (1732.4975 g mol^−1^): C 52.72, H 4.31 and N 3.24; Found: C 52.53, H 4.19 and N 3.33.

**Synthesis of [(η^5^‐C_5_Me_5_){(IDipp)P}Ir(PMe_3_)][BAr^F^] (6)**: To a stirred solution of [(η^5^‐C_5_Me_5_){(IDipp)P}IrCl] (**3 b**, 0.050 g, 0.064 mmol) and PMe_3_ (0.007 g, 0.089 mmol) in fluorobenzene (20 mL) was added NaBAr^F^ (0.056 g, 0.064 mmol) as a solid at room temperature. The resulting mixture was stirred for 2 h and filtered. The solvent was evaporated, and the residue was washed with toluene (3×2 mL) and dried under vacuum to obtain **6** as brown solid. Yield: 0.049 g (46 %). ^1^H NMR ([D_8_]THF, 300.1 MHz); *δ*=7.88 (s, 2 H, *H_2_*C_2_N_2_), 7.30–7.26 (br, 8 H, *H*‐BAr^F^), 7.53 (br, 4 H, *H*‐BAr^F^), 7.50–7.44 (m, 2 H, Ar‐*H*), 7.35‐7‐33 (m, 4 H, *H*‐Ph), 2.78 (sept, 4 H, ^3^ *J*_HH_=7.8 Hz, C*H*(CH_3_)_2_), 1.71 (s, 15 H, C_5_(C*H_3_*)_5_), 1.50 (d, 9 H, ^2^ *J*_PH_=11.5 Hz, P(C*H_3_*)_3_), 1.33 (d, 12 H, ^3^ *J*_HH_=6.7 HZ, CH(C*H_3_*)_2_), 1.14 (d, 12 H, ^3^ *J*_HH_=6.8 Hz, CH(C*H_3_*)_2)_  ppm. ^13^C NMR ([D_8_]THF, 75.4 MHz); 162.4 (m, *C*‐B, B(Ar^F^), 146.6 (N*C*(Dipp)), 135.7 (br, (*m‐C*(BAr^F^), 134.1 (*o‐C*(Dipp)), 132.1 (*p‐C*(Dipp)), 130.2 (qq, ^2^ *J*_CF_=30 Hz, ^3^ *J*_BC_=3.1 Hz, *m‐C*‐BAr^F^), 128.3 (d, ^3^ *J*_PC_=4.5 Hz, H‐*C*_2_N_2_), 125.8 (*m‐C*(Dipp)), 125.2 (q, ^1^ *J*_CF_=271 Hz, *C*F_3_‐BAr^F^), 118.3 (br, *p*‐C(BAr^F^)), 94.7 (d, ^3^ *J*_PC_=3.0 Hz, (*C*_5_(CH_3_)_5_), 30.0 (*C*H(CH_3_)_2_), 26.2 (CH(*C*H_3_)_2_)_,_ 23.1 (CH(*CH_3_*)_2_, 10.9 (C_5_(*CH_3_)*_5_), 7.44 (d, *J*_PC_=3.4 Hz, P(*C*H_3_)_3)_  ppm. ^31^P NMR ([D_8_]THF, 121.4 MHz); *δ*=−33.13 (d, ^2^ *J*_PP_=106.7 Hz, *P*(CH_3_)_3_) and 454.9 (d, ^2^ *J*_PP_=106.7 Hz, (IDipp)*P*) ppm. ^11^B NMR ([D_8_]THF, 96.2 MHz): *δ*=−5.99 ppm. ^19^F NMR ([D_8_]THF, 188.2 MHz): *δ*=−62.43 (s) ppm. HRMS (ESI‐positive ion mode, CH_3_CN/Toluene): *m*/*z*: calcd for the cationic part of the molecule; C_40_H_60_IrN_2_P_2_: 823.3857; found: 823.3858. Anal. calcd (%) for C_72_H_72_BF_24_IrN_2_P_2_ (1686.4509 g mol^−1^); C 51.23, H 4.30 and N 1.66; Found; C 51.86, H 4.07 and N 1.65.

**Synthesis of [(η^5^‐C_5_Me_5_){(IDipp)P}Ir(^Me^IMe)][BAr^F^] (7)**: A Schlenk tube was charged with [(η^5^‐C_5_Me_5_){(IDipp)P}IrCl] (**3 b**, 0.050 g, 0.064 mmol) and 1,3,4,5‐tetramethylimidazolin‐2‐ylidene (0.008 g, 0.064 mmol) dissolved in fluorobenzene (10 mL). NaBAr^F^ (0.057 g, 0.064 mmol) was added as a solid at room temperature. The resulting reaction mixture was stirred for 4 h and then filtered. The solvent was evaporated, and the residue was washed with toluene until the washings were almost colorless. This resulting residue was dried under vacuum to obtain **7** as a brown solid. Yield: 0.069 g (62 %) of a mixture that contained both isomers in approx. 76:24 as confirmed by ^31^P NMR spectroscopy. ***E*** **isomer**: ^1^H NMR ([D_8_]THF, 600.1 MHz): *δ*=7.79–7.78 (m, 8 H, *H*‐BAr^F^), 7.77 (s, 2 H, *H_2_*C_2_N_2_), 7.58–7.54 (br, 4 H, H‐BAr^F^), 7.48 (tr, 2 H, *J*_HH_=7.8 Hz, *p‐H*Dipp), 7.34 (d, 4 H, *J*_HH_=7.7 Hz, *m‐H*Dipp), 3.76 (s, 6 H, *N*‐C*H_3_*, ^Me^IMe), 2.77 (sept, 4 H, *J*_HH_=6.8 Hz, C*H*(CH_3_)_2_), 2.26 (s, 6 H, C‐C*H_3_*, ^Me^IMe), 1.70 (brs, 15 H, C_5_(C*H_3_*)_5_), 1.32 (d, *J*_HH_=6.8 Hz, CH(C*H_3_*)_2_), 1.14 (d, *J*_HH_=6.8 Hz, CH(C*H_3_*)_2)_  ppm. ^13^C NMR ([D_8_]THF, 150.9 MHz); *δ*=162.9 (q, *C*‐B, BAr^F^), 146.6 (N*C*(Dipp)), 135.7 (br, (*m‐C*(BAr^F^), 134.9 (*m‐C*(Dipp)), 131.6 (*p‐C*(Dipp)), 130.2 (qq, ^2^ *J*_CF_=30 Hz, ^3^ *J*_BC_=3.1 Hz, *m‐C*‐BAr^F^), 128.8 (C_2_N_2_, ^Me^IMe), 127.17 (H_2_ *C_2_*N_2_, overlapped with other isomer), 125.5 (*m‐C*(Dipp)), 125.6 (q, ^1^ *J*_CF_=272 Hz, *C*F_3_‐BAr^F^), 118.3 (sept, *J*_CF_=4.1 Hz, *p*‐C(BAr^F^)), 92.5 (*C*_5_CH_3_)_5_), 33.6 (*N*‐*C*H_3_, ^Me^IMe), 29.9 (*C*H(CH)_3_)_2_, 25.6 (CH(*C*H_3_)_2_, 22.31 (CH(*C*H)_3_)_2_, 10.9 (C_5_(*C*H_3_)_5_), 7.8 (C‐*C*H_*3*_, ^Me^IMe) ppm. ^31^P NMR ([D_8_]THF, 121.5 MHz): *δ*=399.0 (s) ppm. ^11^B NMR([D_8_]THF, 96.3 MHz): *δ*=−6.0 (s) ppm. ^19^F NMR ([D_8_]THF, 188.2 MHz): *δ*=−62.4 ppm. UV/Vis (THF, λ(nm) *ϵ*(M^−1^ cm^−1^)): 341.7 (7579.7), 473.9 (3124.6) and 600.4 (823.6). ***Z isomer***: 7.78–7.75 (m, 8 H, *H*‐BAr^F^), 7.73 (s, 2 H, *H_2_*C_2_N_2_), 7.57–7.56 (br, 4 H, H‐BAr^F^), 7.49 (tr, 2 H, *J*_HH_=7.7 Hz, *p‐H*Dipp), 7.28 (d, 4 H, *J*_HH_=7.9 Hz, *m‐H*Dipp), 3.28 (s, 6 H, *N*‐C*H_3_*, ^Me^IMe), 2.65 (sept, 4 H, *J*_HH_=6.6 Hz, C*H*(CH_3_)_2_), 2.26 (s, 6 H, C‐C*H_3_*, ^Me^IMe), 1.71 (brs, 15 H, C_5_(C*H_3_*)_5_), 1.13 (d, *J*_HH_=6.6 Hz, CH(C*H_3_*)_2_), 1.04 (d, *J*_HH_=6.6 Hz, CH(C*H_3_*)_2)_  ppm. ^13^C NMR ([D_8_]THF, 150.9 MHz): *δ*=163.0 (m, *C*‐B, BAr^F^), 146.3 (N*C*(Dipp)), 135.7 (br, (*m‐C*(BAr^F^), 135.3 (*m‐C*(Dipp)), 131.4 (*p‐C*(Dipp)), 130.2 (qq, ^2^ *J*_CF_=30 Hz, ^3^ *J*_BC_=3.1 Hz, *m‐C*‐BAr^F^), 128.8 (*C_2_*N_2_, ^Me^IMe), 127.18 (H_2_ *C_2_*N_2_, overlapped with another isomer)), 126.2 (*C_2_*N_2_, ^Me^IMe), 125.7 (*m‐C*(Dipp)), 125.6 (q, ^1^ *J*_CF_=272 Hz, *C*F_3_‐BAr^F^), 118.3 (sept, *J*_CF_=4.1 Hz, *p*‐C(BAr^F^)), 90.2 (*C*_5_CH_3_)_5_), 37.7 (*N*‐*C*H_3_, ^Me^IMe), 30.3 (*C*H(CH)_3_)_2_, 24.23 (CH(*C*H)_3_)_2_, 22.95 (CH(*C*H)_3_)_2_, 10.19 (C_5_(*C*H_3_)_5_), 9.3 (C‐*C*H_*3*_, ^Me^IMe) ppm. ^31^P NMR ([D_8_]THF, 121.5 MHz): *δ*=483.8 ppm. ^11^B NMR ([D_8_]THF, 96.3 MHz): *δ*=−6.0 (s) ppm. ^19^F NMR ([D_8_]THF, 188.2 MHz): *δ*=−62.4 ppm. HRMS (positive ion mode, CH_3_CN/toluene): *m*/*z*: calcd for the cationic fragment C_44_H_63_IrPN_4_: 871.4416; found: 871.4406 (73 %).

**Synthesis of [(η^5^‐C_5_Me_5_){(IDipp)P}Ir(CO)][BAr^F^] (8)**: Fluorobenzene (20 mL) was added to a Schlenk tube containing [(η^5^‐C_5_Me_5_){(IDipp)P}IrCl] (**3 b**, 0.082 g, 0.105 mmol) and NaBAr^F^ (0.93 mg, 0.105 mol) at room temperature. Carbon monoxide gas was bubbled continuously through the dark blue reaction mixture for 1 h. The solvent was evaporated to obtain brown‐red oil, which was washed with cold toluene followed by *n*‐hexane to afford **8** as a purple red solid. Yield: 0.087 g (51 %). ^1^H NMR ([D_8_]THF, 300.1 MHz); *δ*=8.06 (s, 2 H, *H*C_2_N_2_), 7.76–7.73 (br, 8 H, *H*‐BAr^F^), 7.54 (br, 4 H, *H*‐BAr^F^), 7.49–7.44 (m, 2 H, Ar‐H), 7.35‐7‐31 (m, 4 H, *H*‐Ph), 2.71 (sept, 4 H, ^3^ *J*_HH_=6.7 Hz, C*H*(CH_3_)_2_), 1.94 (s, 15 H, C_5_(C*H_3_*)_5_),1.23 (d, 12 H, ^3^ *J*_HH_=6.7 Hz, CH(C*H_3_*)_2_), 1.17 (d, 12 H, ^3^ *J*_HH_=6.8 Hz, CH(C*H_3_*)_2)_  ppm. ^13^C NMR ([D_8_]THF, 75.4 MHz); *δ*=not visible (*C*O‐Ir), not visible (P‐*C_carbene_*), 163.0 (q, ^1^ *J*_BC_=50.2 Hz, *C*‐B, B(Ar^F^)), 146.6 (N*C*(Dipp)), 135.7 (*o‐C*(BAr^F^), 133.0 (*o‐C*(Dipp)), 132.5 (*p‐C*(Dipp)), 130.2 (qq, ^2^ *J*_CF_=31 Hz, ^3^ *J*_BC_=3.5 Hz, *m‐C*‐BAr^F^), 128.3 (H‐*C*_2_N_2_), 125.9 (*m‐C*(Dipp)), 125.7 (q,^1^ *J*_CF_=271 Hz, *C*F_3_‐BAr^F^), 118.4 (sept, ^3^ *J*_FC_=4.4 Hz, *p*‐C(BAr^F^)), 103.6 (*C*_5_(CH_3_)_5_), 30.1 (*C*H(CH_3_)_2_), 25.9 (CH(*C*H_3_)_2_)_,_ 23.2 (CH(*CH_3_*)_2_ and 9.9 (C_5_(*C*H_3_ *)*_5)_  ppm. ^31^P NMR ([D_8_]THF, 121.4 MHz); *δ*=597.0 (s, IDipp)*P*‐Ir) ppm. ^11^B NMR ([D_8_]THF, 96.2 MHz): *δ*=−6.03 ppm. ^19^F NMR ([D_8_]THF, 188.2 MHz): *δ*=−62.43 (s) ppm. IR (cm^−1^) (as neat solid in ATR mode); 1998.3 (*ṽ*_CO_). HRMS (ESI‐MS, positive ion mode, CH_3_CN): *m*/*z*: calcd for the cationic fragment C_38_H_51_N_2_OPIr (calcd 775.3363); found: 775.3376. Anal. Calcd (%) for C_70_H_63_BF_24_IrN_2_OP (1638.4017 g mol^−1^): C 51.27, H 3.87, N 1.71; Found: C 51.02, H 3.82 and N 1.69.

## Conflict of interest

The authors declare no conflict of interest.

## Supporting information

As a service to our authors and readers, this journal provides supporting information supplied by the authors. Such materials are peer reviewed and may be re‐organized for online delivery, but are not copy‐edited or typeset. Technical support issues arising from supporting information (other than missing files) should be addressed to the authors.

Supplementary
