# Supplementary material for: N‐Heterocyclic Carbene Analogues of Nucleophilic Phosphinidene Transition Metal Complexes
Source: Chemistry. 2020 Oct 6;26(65):14878–87. doi: 10.1002/chem.202003099 (PMC7756676; doi:10.1002/chem.202003099)
Supplement: Supplementary file 1 — Supplementary [file CHEM-26-14878-s001.pdf]

Chemistry–A European Journal

Supporting Information

N-Heterocyclic Carbene Analogues of Nucleophilic Phosphinidene Transition Metal Complexes

Adinarayana Doddi,*[a] Dirk Bockfeld,[b] Thomas Bannenberg,[b] and Matthias Tamm*[b]

Supporting Information

N-Heterocyclic Carbene Analogues of Nucleophilic Phosphinidene Transition Metal Complexes

Adinarayana Doddi,* Dirk Bockfeld, Thomas Bannenberg and Matthias Tamm*

A) Spectra of the Complexes 4–8

B) Crystallographic Data

C) Theoretical Calculations

D) References

A) Spectra of the Complexes 4–8

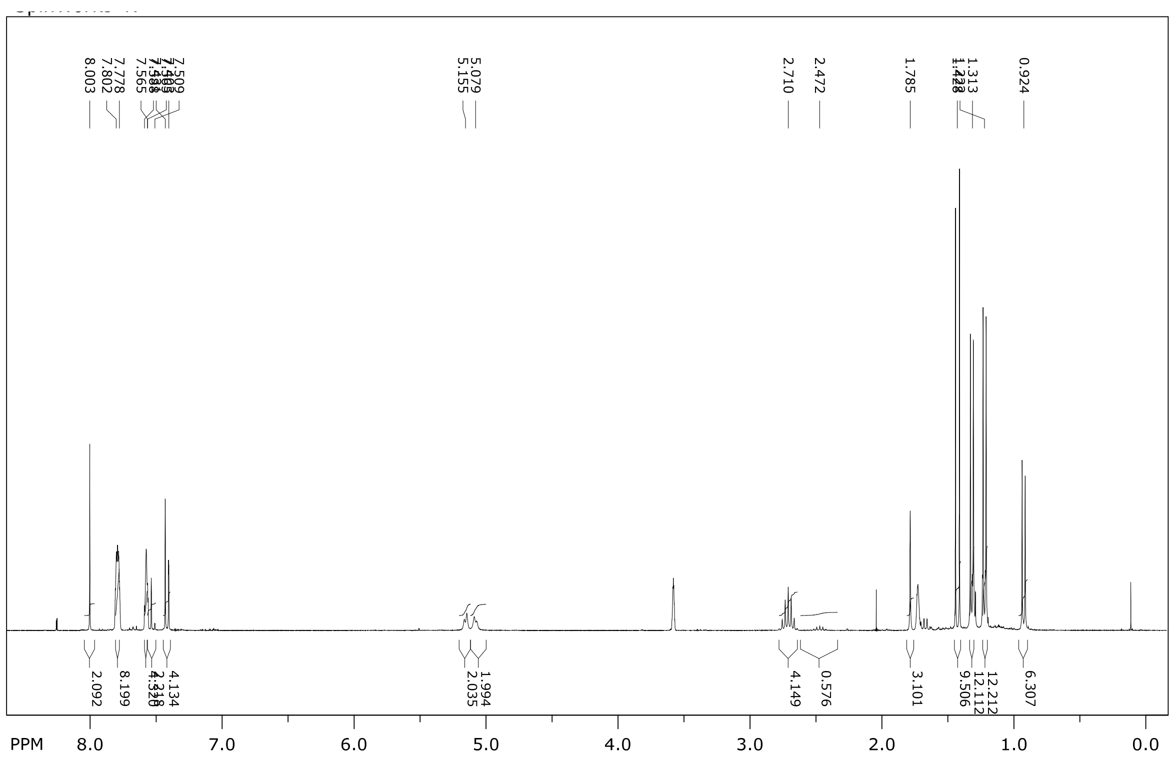

Figure S1. ^{1}H NMR spectrum of [(η^{6}-*p*-cymene){(IDipp)P}Ru(PMe$_3$)][BAr^F] (**4a**) in THF-*d*$_8$ at room temperature.

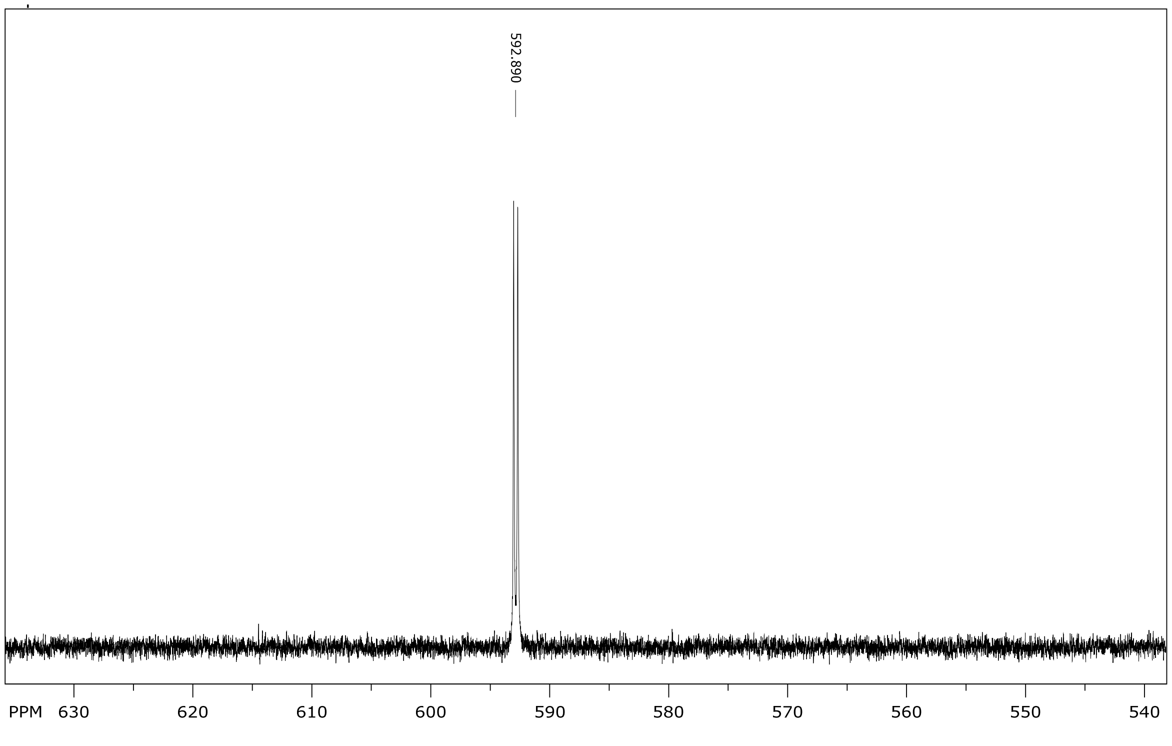

Figure S2. ^{31}P {^{1}H} NMR spectrum of [(η^{6}-*p*-cymene){(IDipp)P}Ru(PMe$_3$)][BAr^F] (**4a**) in THF-*d*$_8$ at room temperature (δ = 592.89 (d) ppm, J_{PP} = 41.32 Hz).

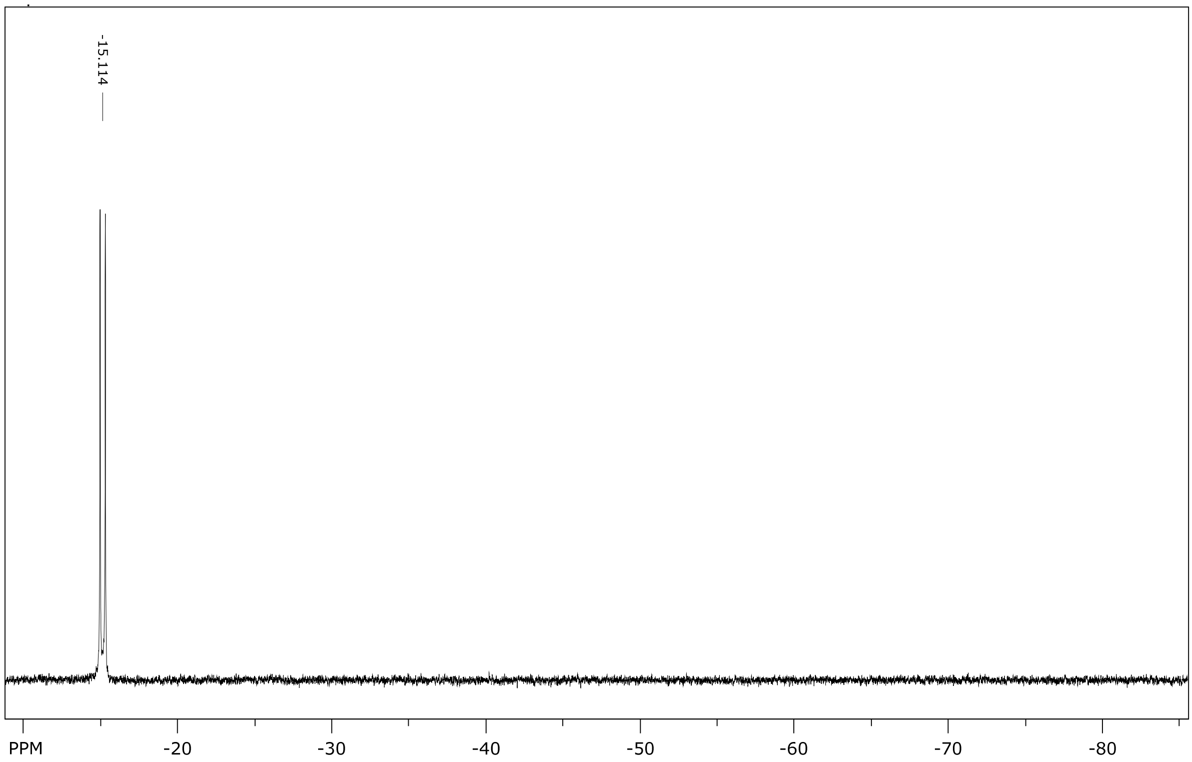

Figure S3. ^{31}P {^{1}H} NMR spectrum of [(η^6-*p*-cymene){(IDipp)P}Ru(PMe_3)][BAr^F] (**4a**) in THF-d_8 at room temperature (δ = –15.11 (d) ppm, J_{PP} = 41.32 Hz).

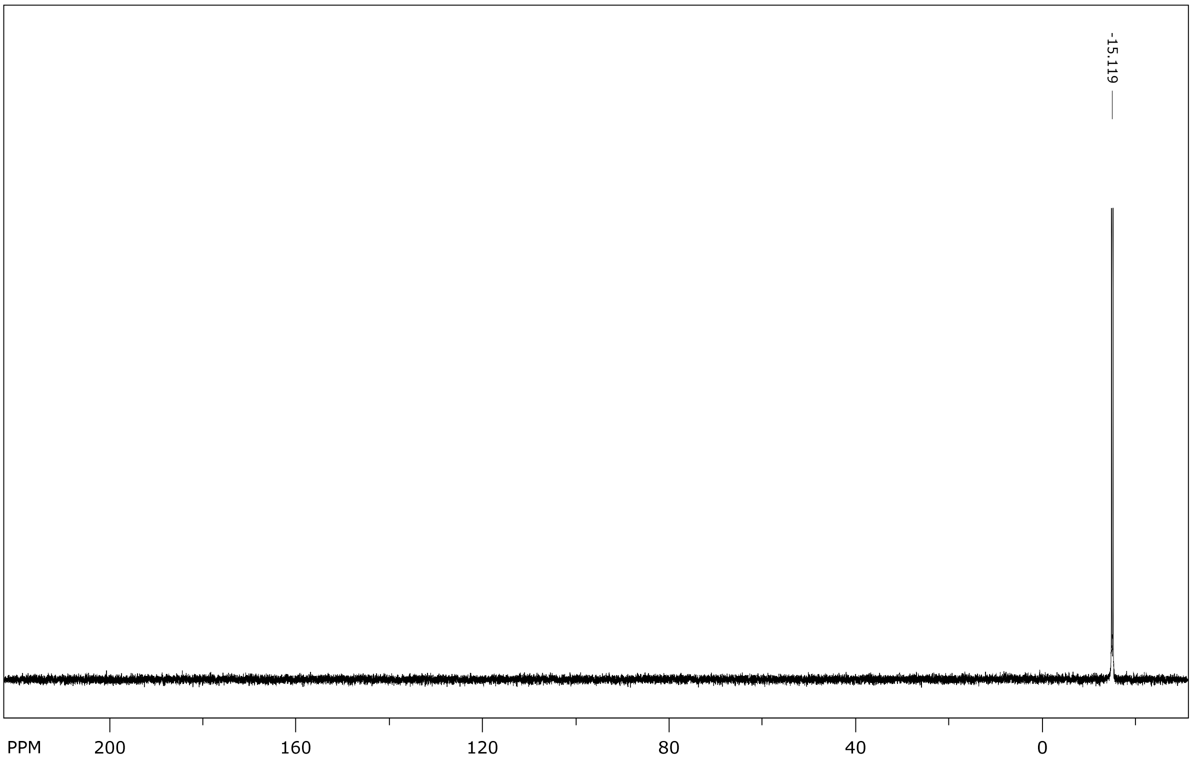

Figure S4. ^{31}P {^{1}H} NMR spectrum of [(η^6-*p*-cymene){(IDipp)P}Ru(PMe_3)][BAr^F] (**4a**) in THF-d_8 at room temperature (δ = –15.11 (d) ppm, J_{PP} = 41.32 Hz).

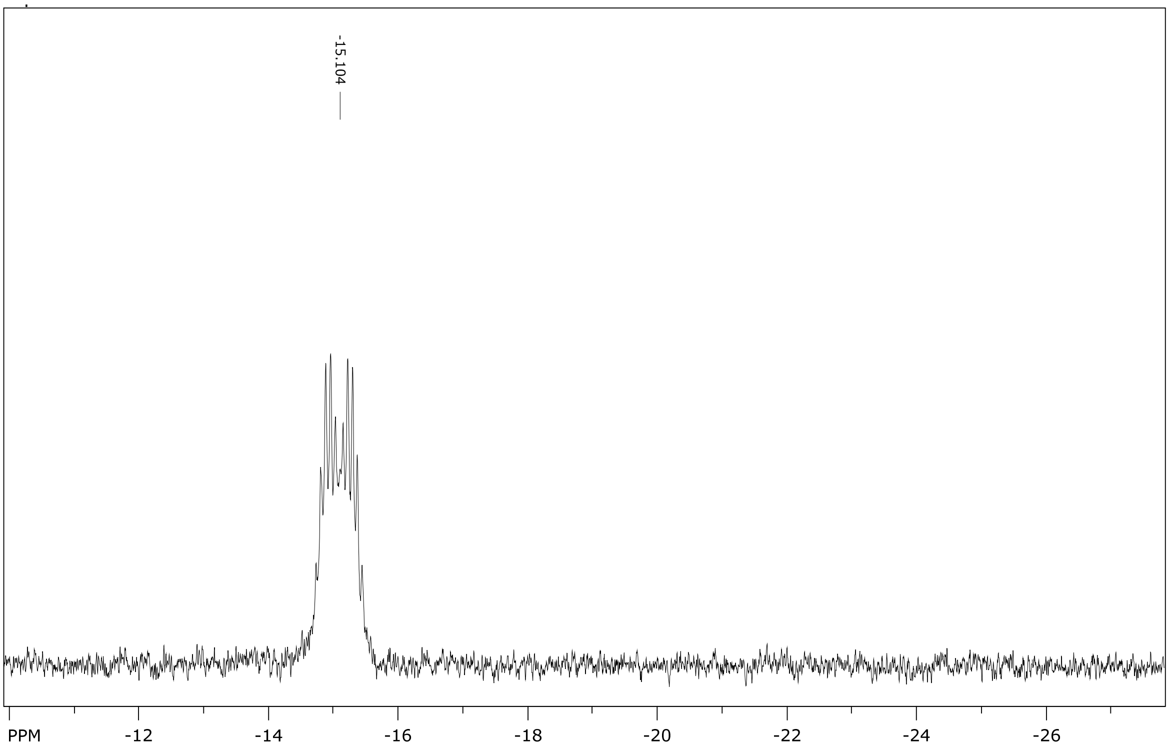

Figure S5. ^{31}P NMR spectrum of [(η^6-*p*-cymene){(IDipp)P}Ru(PMe_3)][BAr^F] (**4a**) in THF-d_8 at room temperature.

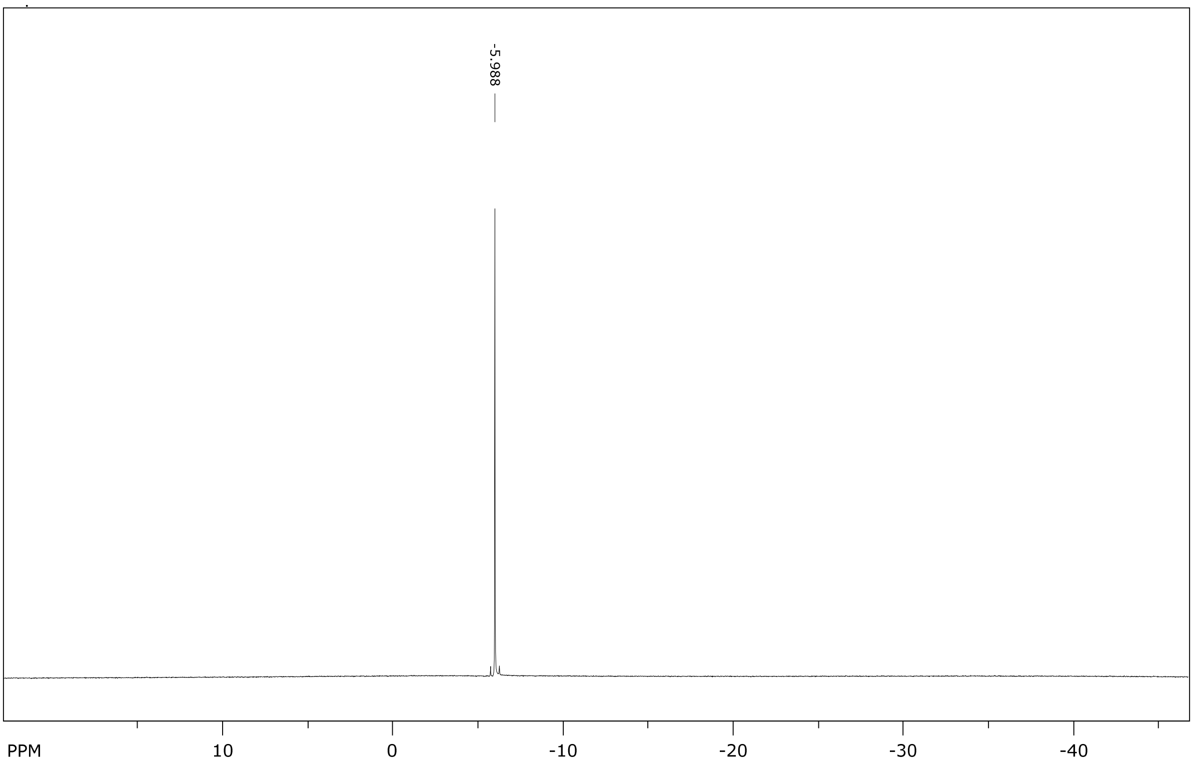

Figure S6. ^{11}B NMR spectrum of [(η^6-*p*-cymene){(IDipp)P}Ru(PMe_3)][BAr^F] (**4a**) in THF-d_8 at room temperature.

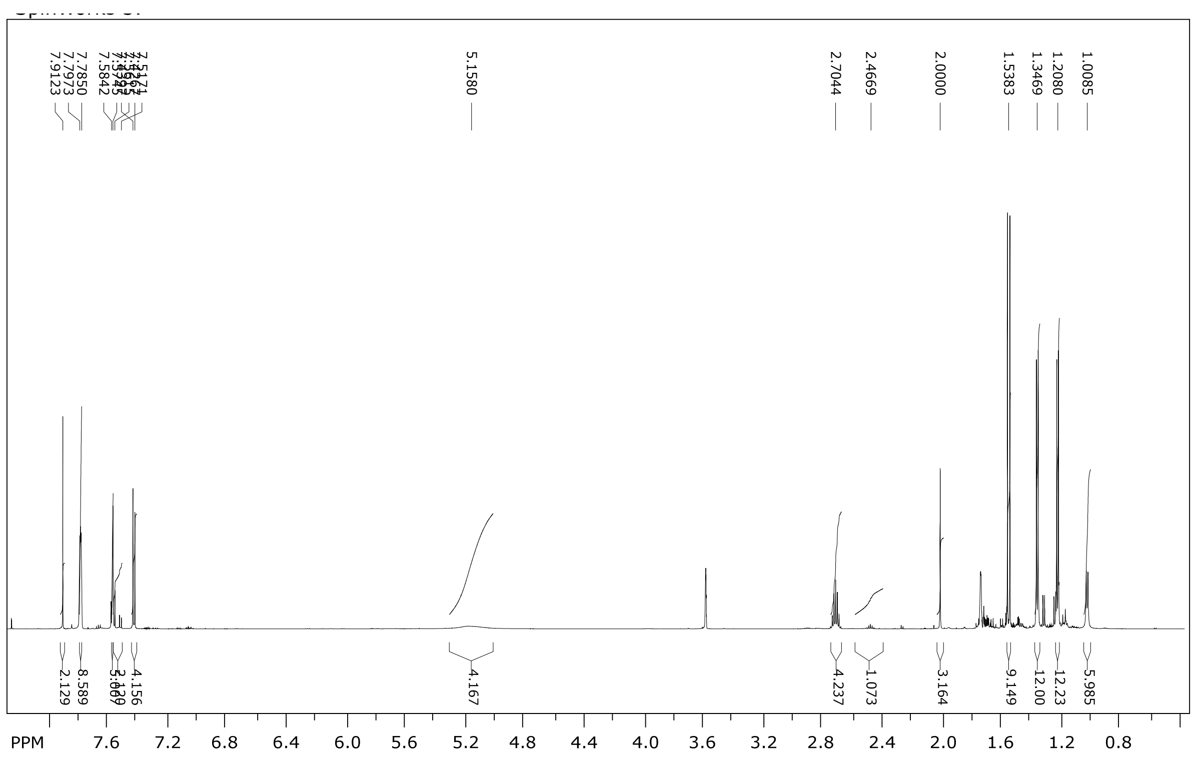

Figure S7. ^{1}H NMR spectrum of [(η^6-*p*-cymene){(IDipp)P}Os(PMe_3)][BAr^F] (**4b**) in THF-d_8 at room temperature.

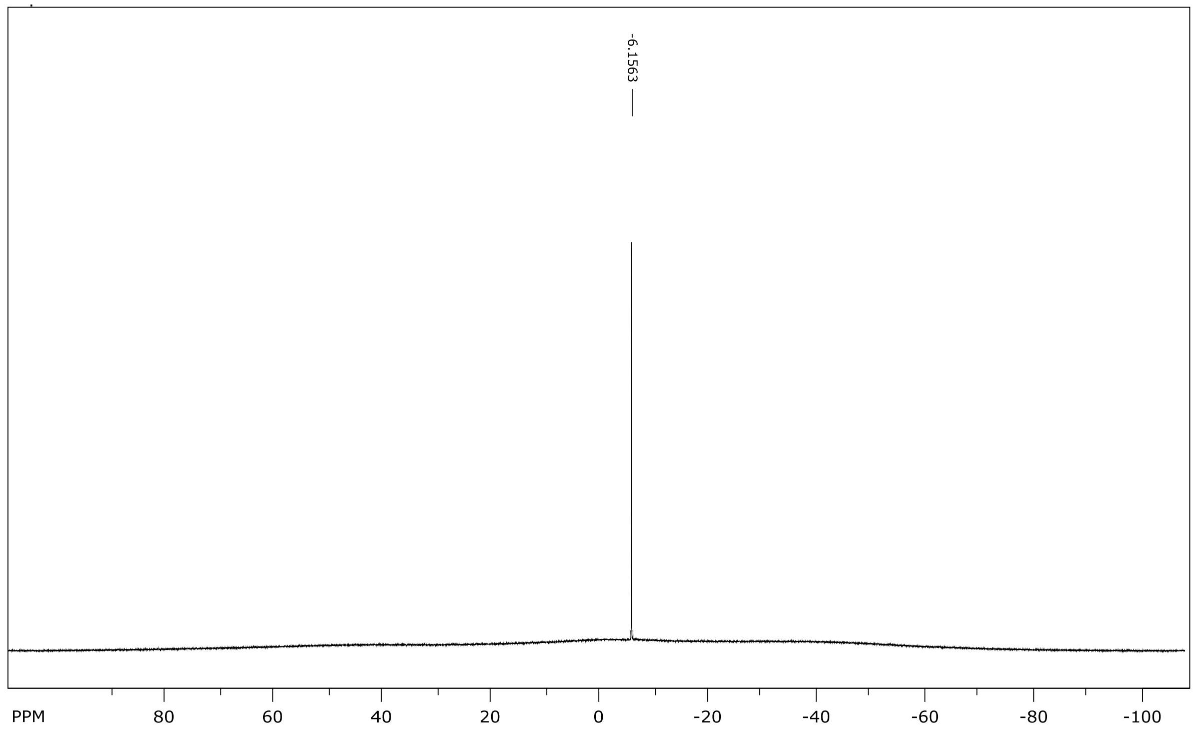

Figure S8. ^{11}B NMR spectrum of [(η^6-*p*-cymene){(IDipp)P}Os(PMe_3)][BAr^F] (**4b**) in THF-d_8 at room temperature.

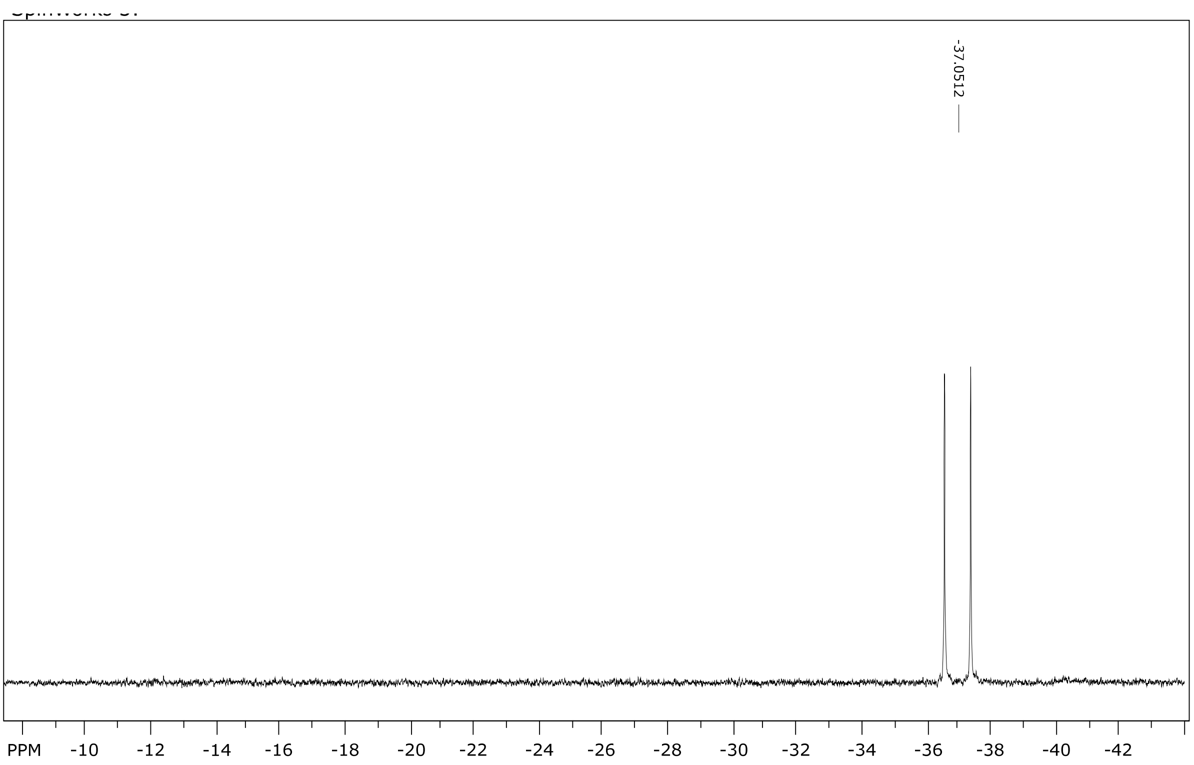

Figure S9. ^{31}P NMR spectrum of [(η^6-*p*-cymene){(IDipp)P}Os(PMe_3)][BAr^F] (**4b**) in THF-d_8 at room temperature.

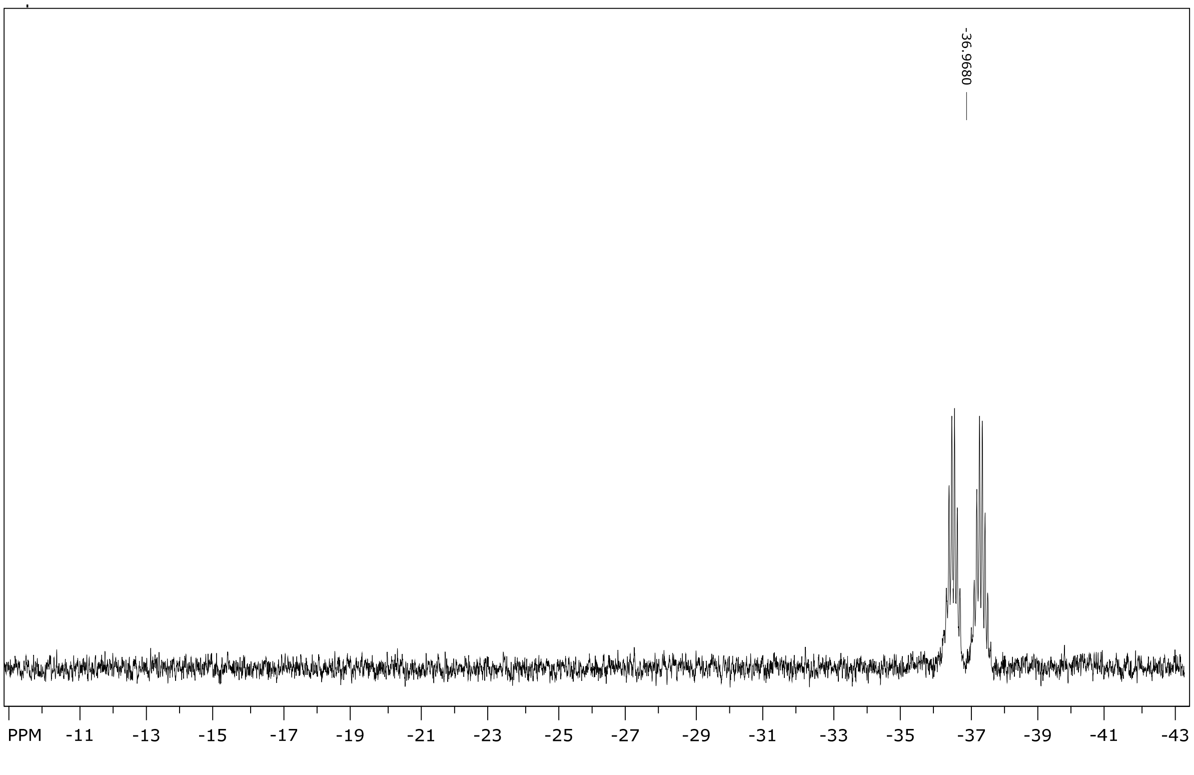

Figure S10. Proton coupled ^{31}P NMR spectrum of [(η^6-*p*-cymene){(IDipp)P}Os(PMe_3)][BAr^F] (**4b**) in THF-d_8 at room temperature.

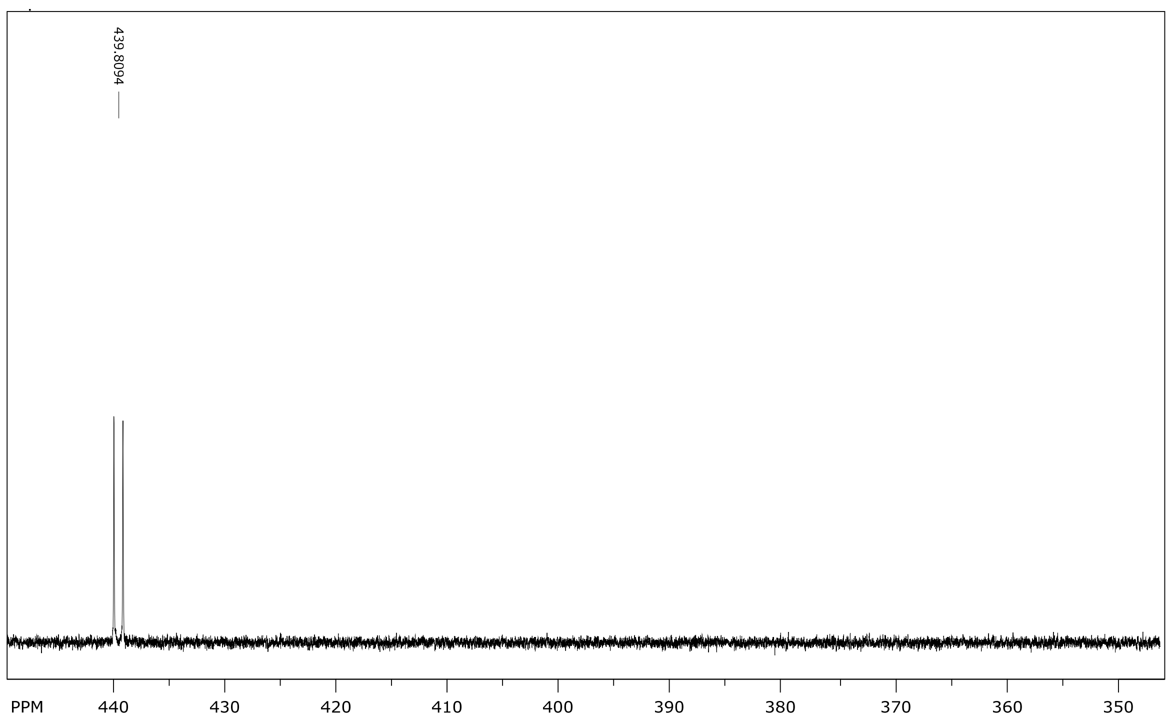

Figure S11. ^{31}P NMR spectrum of [(η^6-*p*-cymene){(IDipp)P}Os(PMe_3)][BAr^F] (**4b**) in THF-d_8 at room temperature.

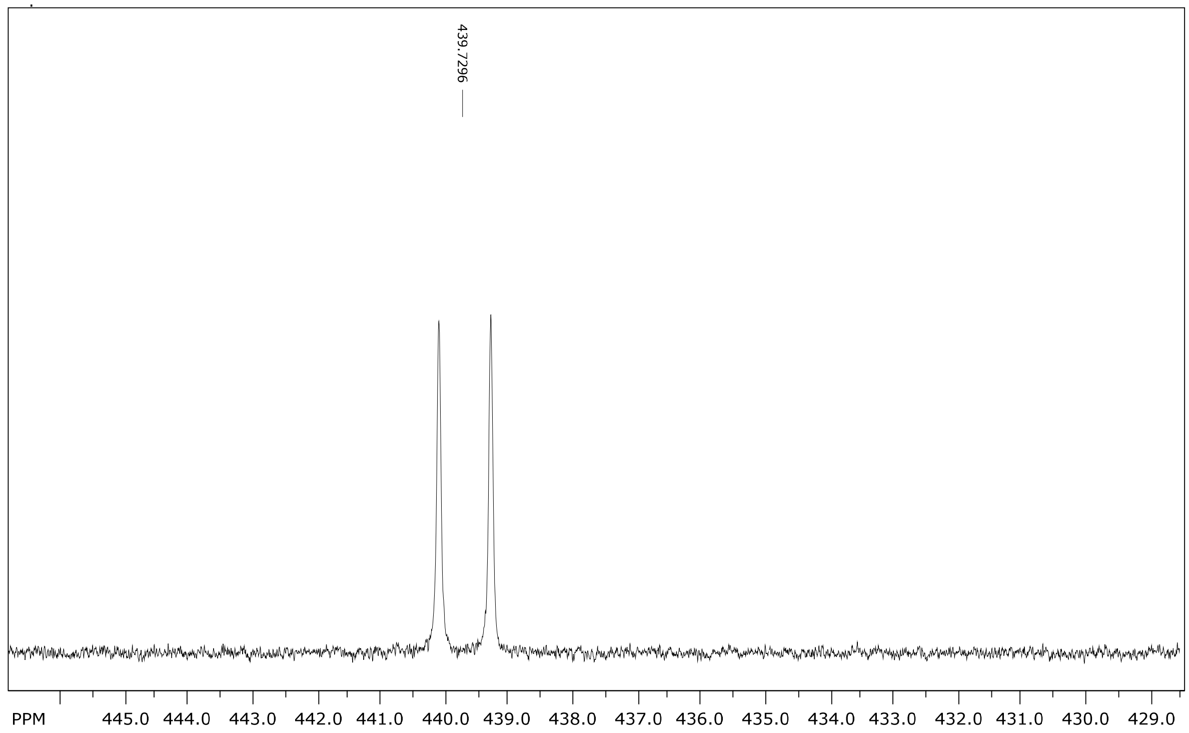

Figure S12. Proton coupled ^{31}P NMR spectrum of [(η^6-*p*-cymene){(IDipp)P}Os(PMe_3)][BAr^F] (**4b**) in THF-d_8 at room temperature.

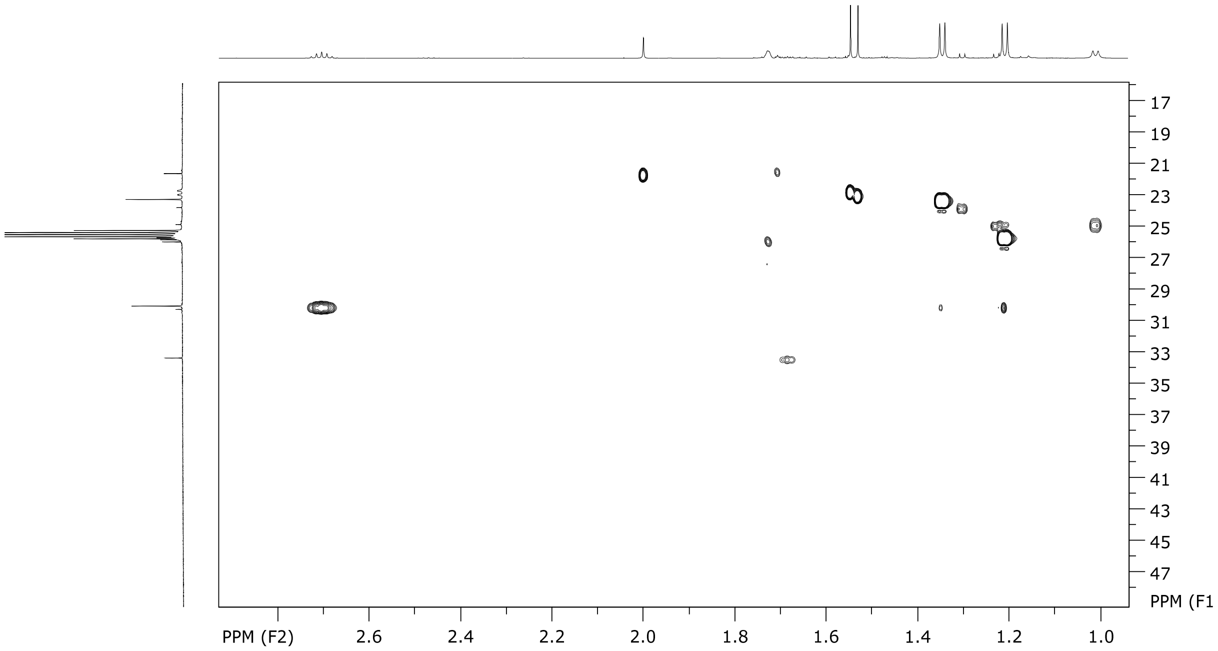

Figure S13. HSQC spectrum of the complex [(η^6-*p*-cymene){(IDipp)P}Os(PMe_3)][BAr^F] (**4b**) (expanded).

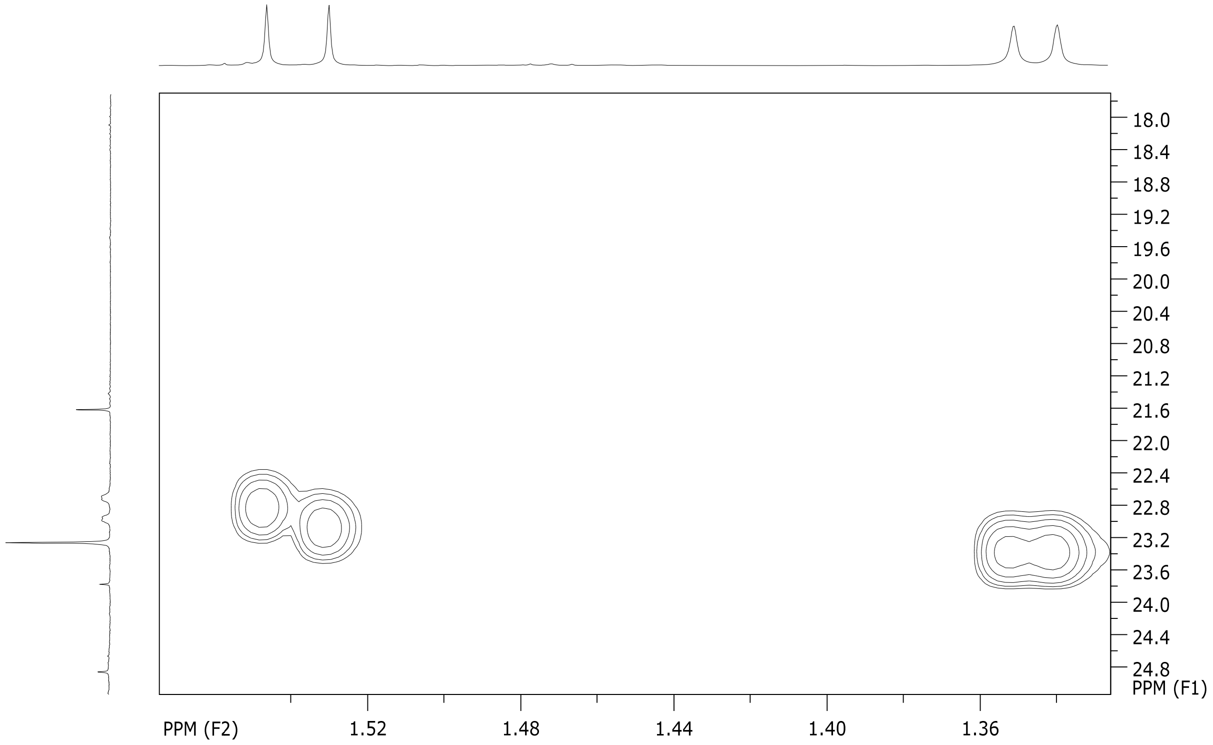

Figure S14. HSQC spectrum of the complex [(η^6-*p*-cymene){(IDipp)P}Os(PMe_3)][BAr^F] (**4b**) (expanded).

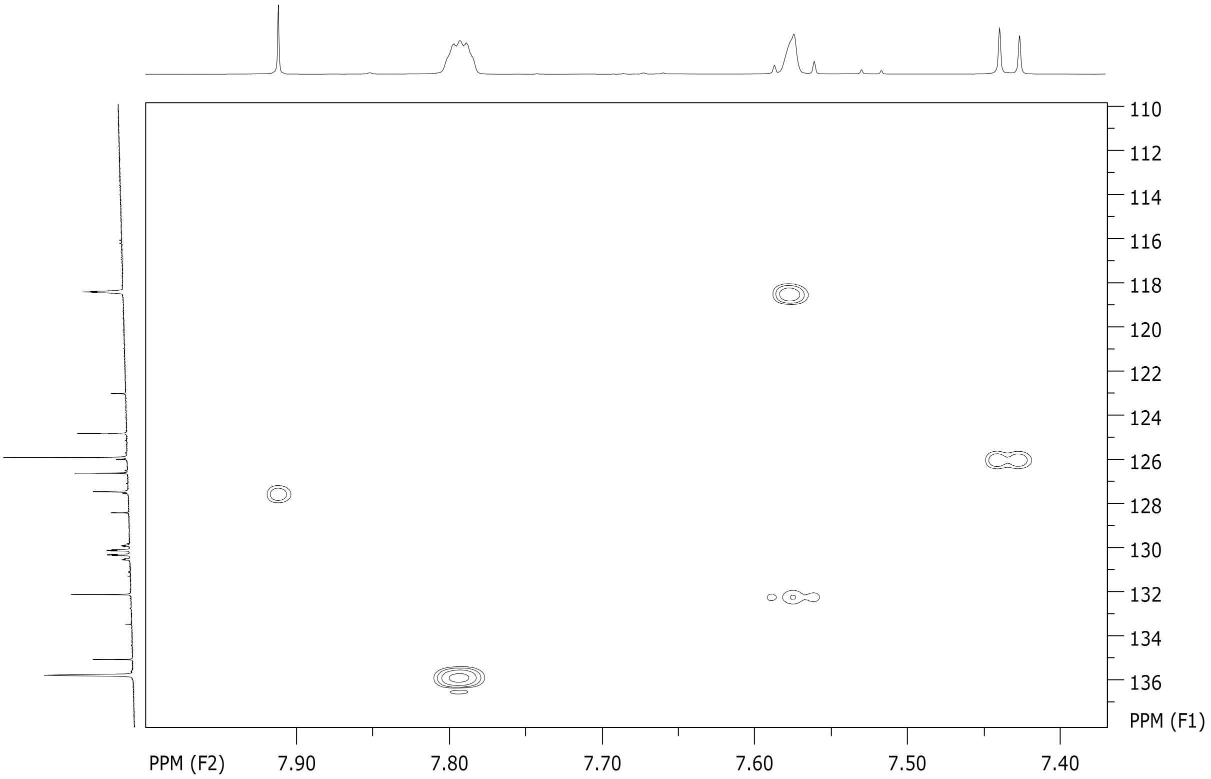

Figure S15. HSQC spectrum of the complex [(η[6]-*p*-cymene){(IDipp)P}Os(PMe₃)][BAr[F]] (**4b**) (expanded).

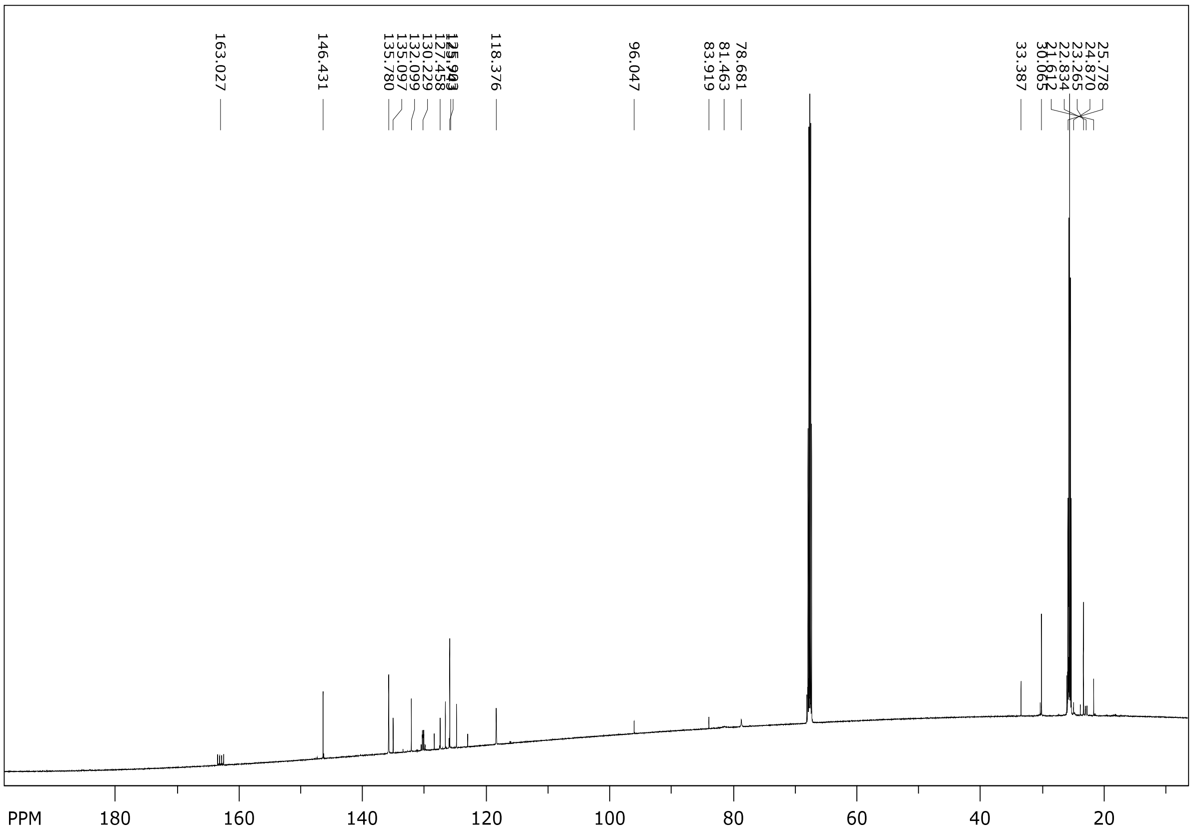

Figure S16. ^{13}C NMR spectrum of [(η[6]-*p*-cymene){(IDipp)P}Os(PMe₃)][BAr[F]] (**4b**) in THF-*d*₈ at room temperature.

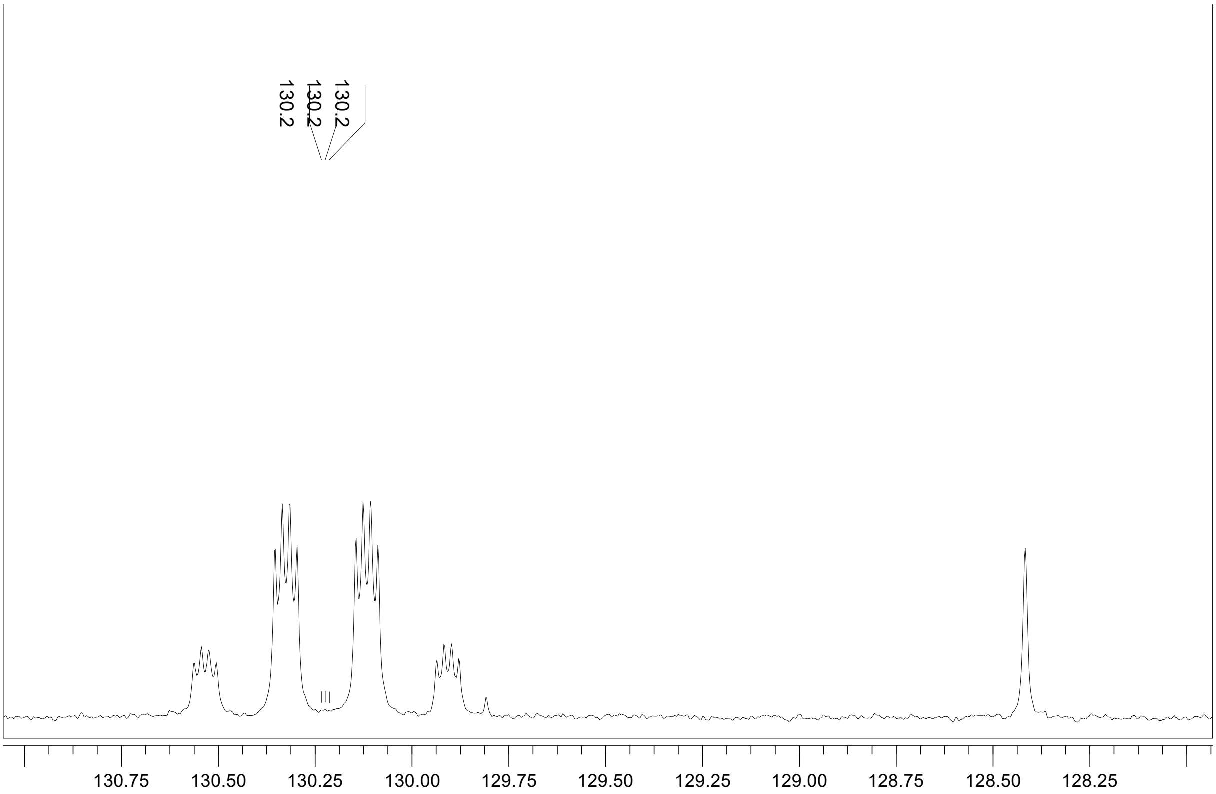

Figure S17. Expanded region of ^{13}C NMR spectrum of [(η^6-*p*-cymene){(IDipp)P}Os(PMe_3)][BAr^F] (**4b**).

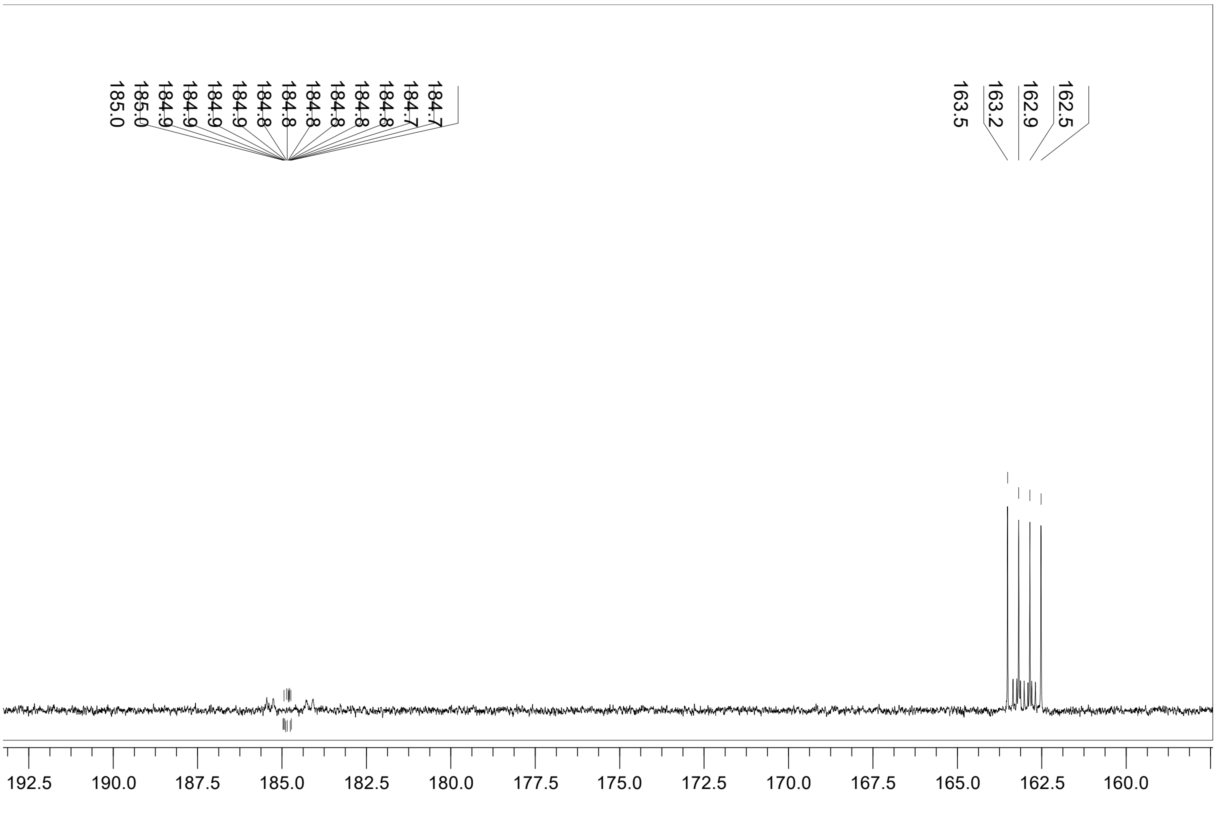

Figure S18. ^{13}C NMR spectrum of [(η^6-*p*-cymene){(IDipp)P}Os(PMe_3)][BAr^F] (**4b**) (expanded region).

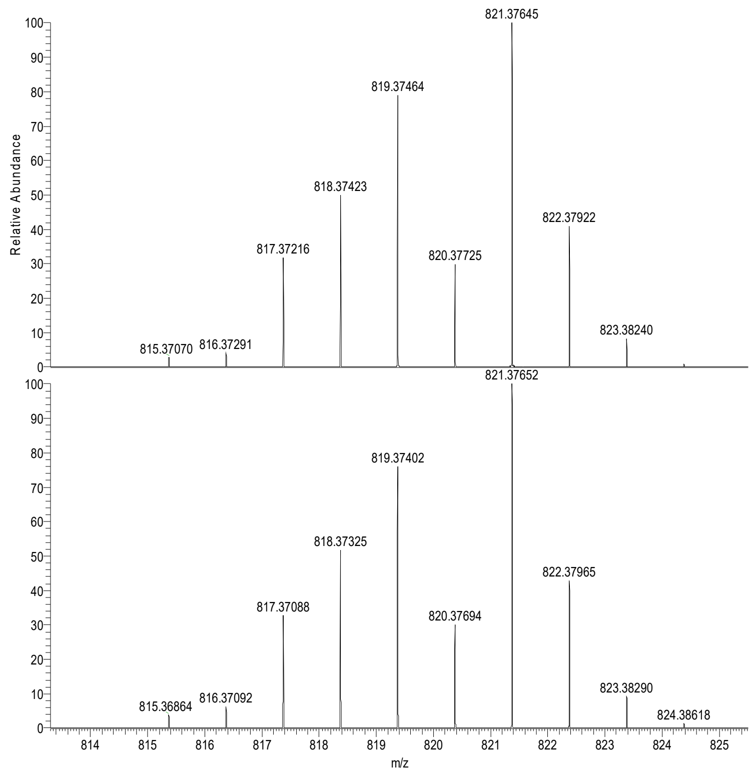

Figure S19. HRMS- of the complex [(η[6]-*p*-cymene){(IDipp)P}Os(PMe_3)][BAr[F]] (**4b**) in positive mode, shows the cationic part of the complex (*top*: experimental isotopic pattern, bottom: computed isotopic pattern).

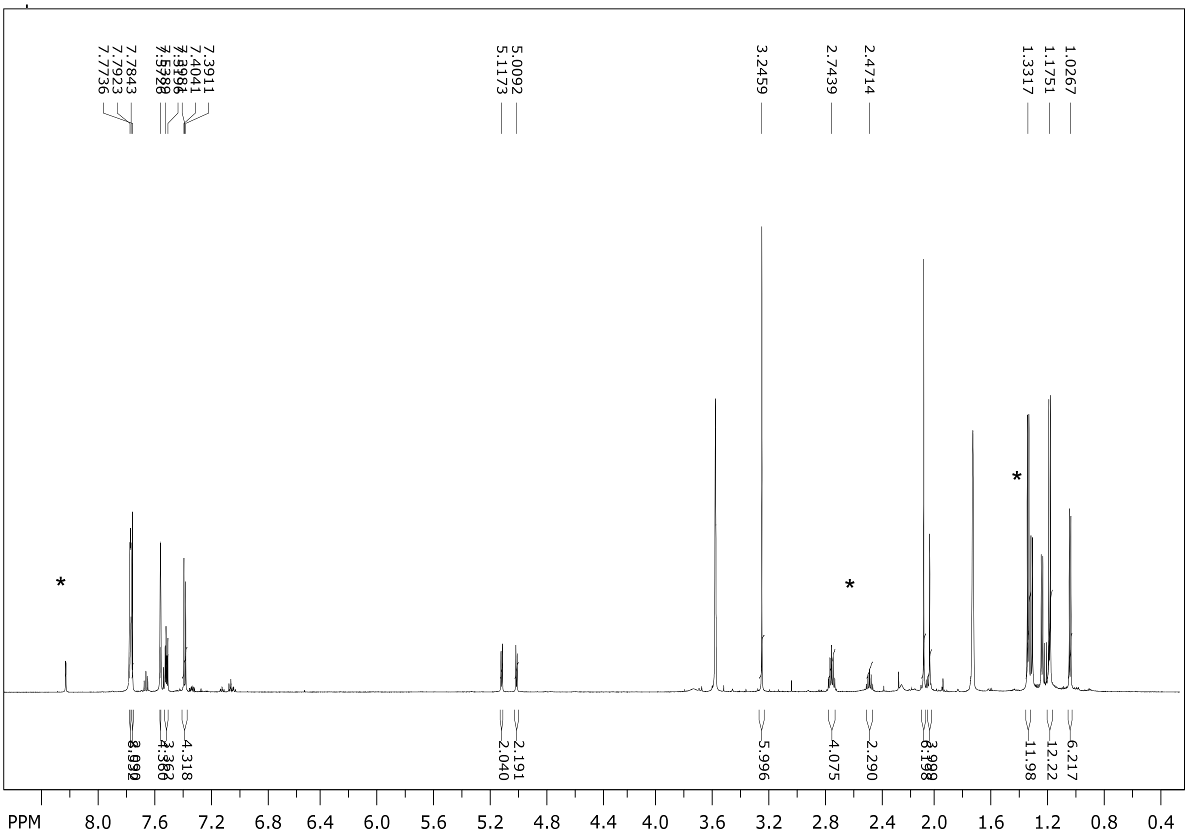

Figure S20. ^{1}H NMR spectrum of complex [(η[6]-*p*-cymene){(IDipp)P}Os([Me]IMe)][BAr[F]] (**5**) in THF-d_8 (*impurity; imidazolium salt).

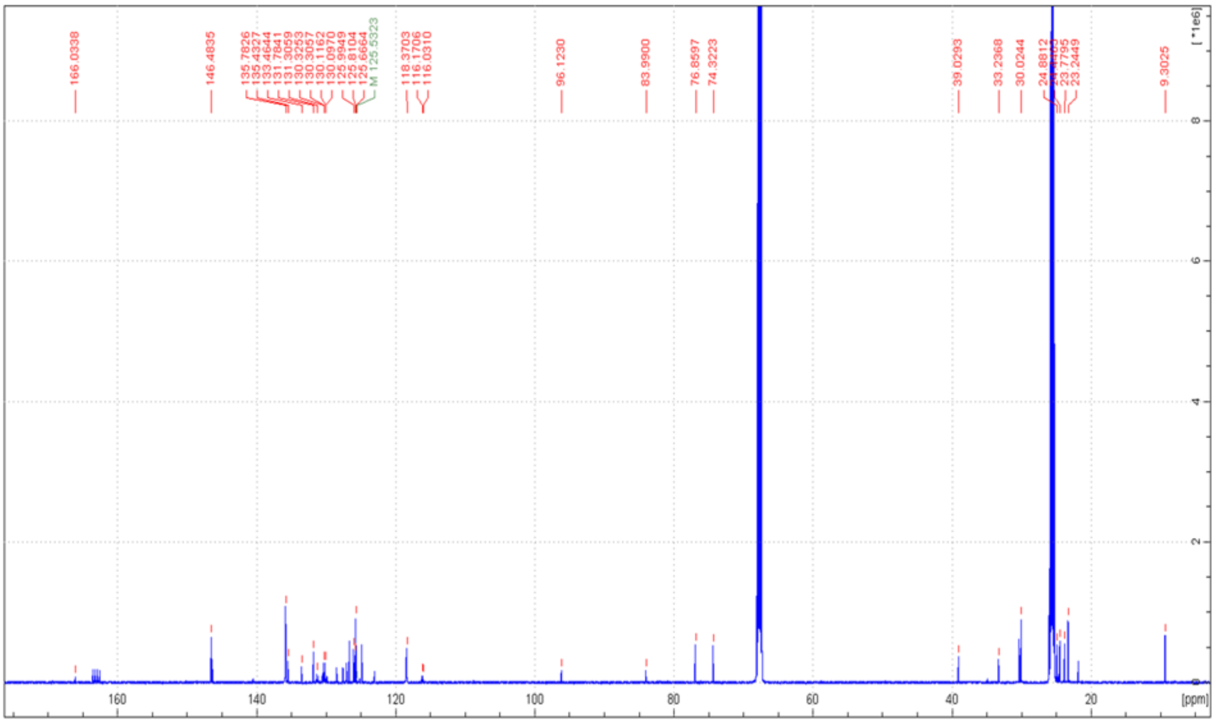

Figure S21. ^{13}C NMR spectrum of complex [(η^6-*p*-cymene){(IDipp)P}Os(^{Me}IMe)][BAr^F] (**5**) in THF-*d*$_8$ at room temperature.

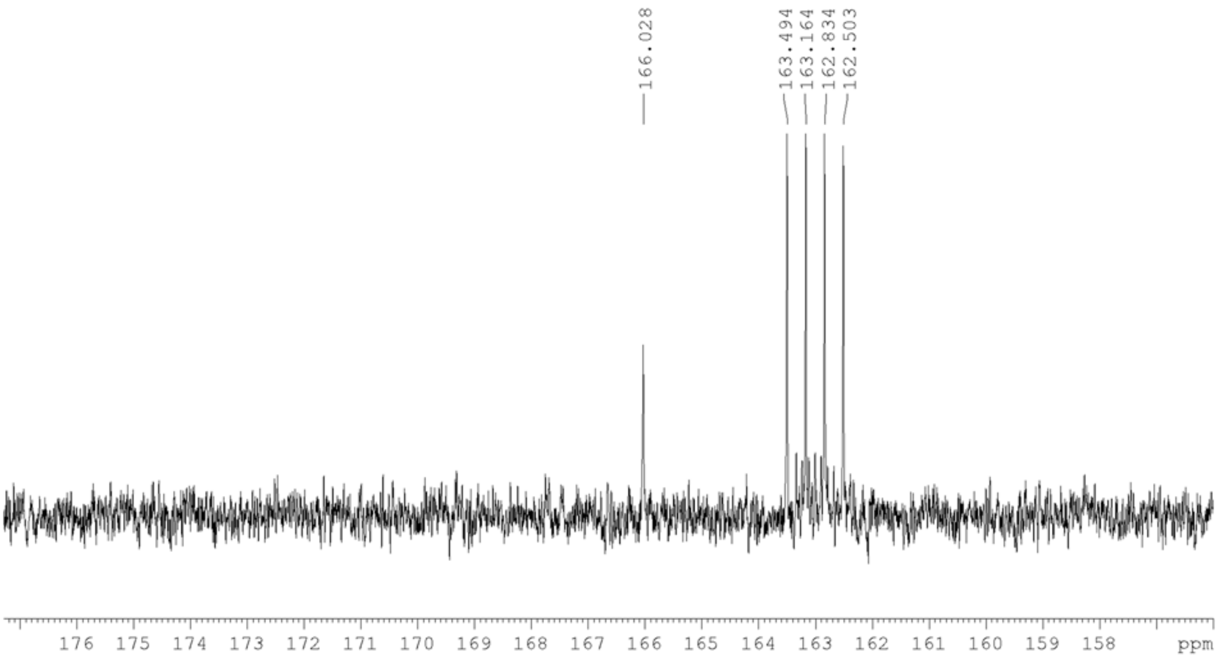

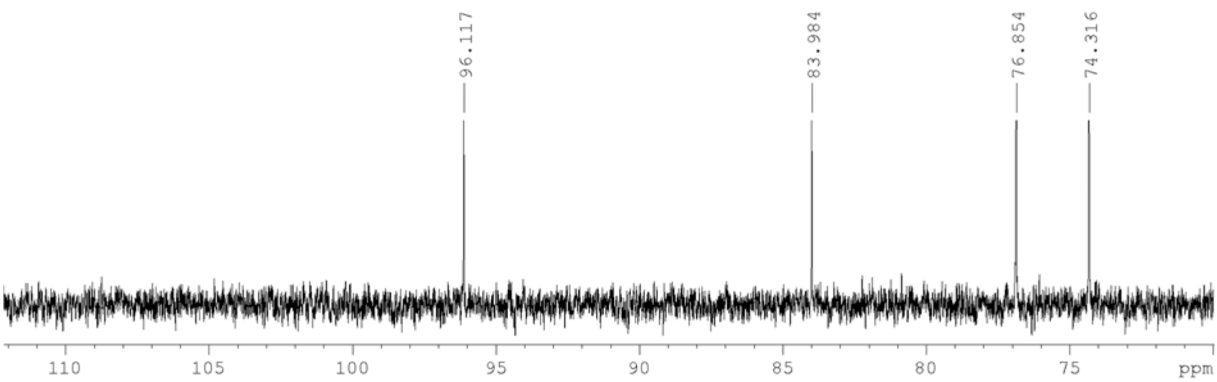

Figure S22. ^{13}C NMR spectrum of [(η^6-*p*-cymene){(IDipp)P}Os(^{Me}IMe)][BAr^F] (**5**) (expanded).

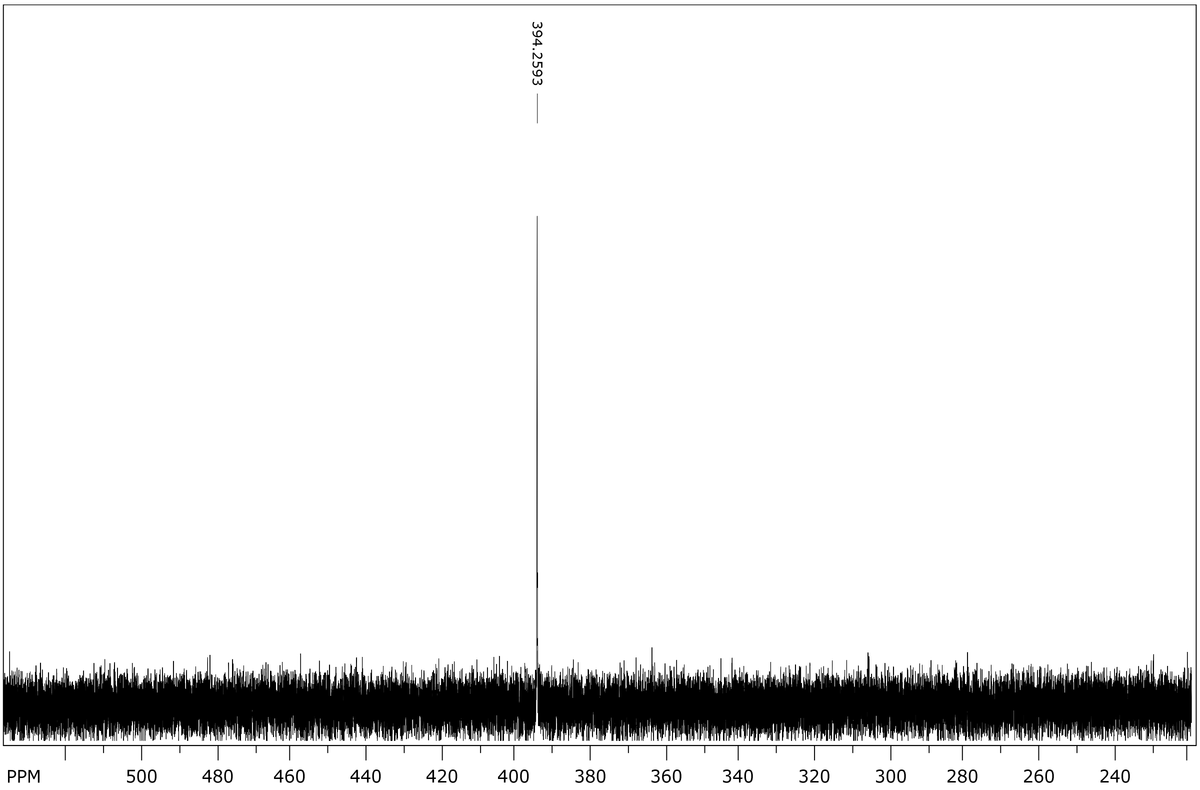

Figure S23. ^{31}P NMR spectrum of [(η^6-*p*-cymene){(IDipp)P}Os(^{Me}IMe)][BAr^F] (**5**) in THF-d_8 at room temperature.

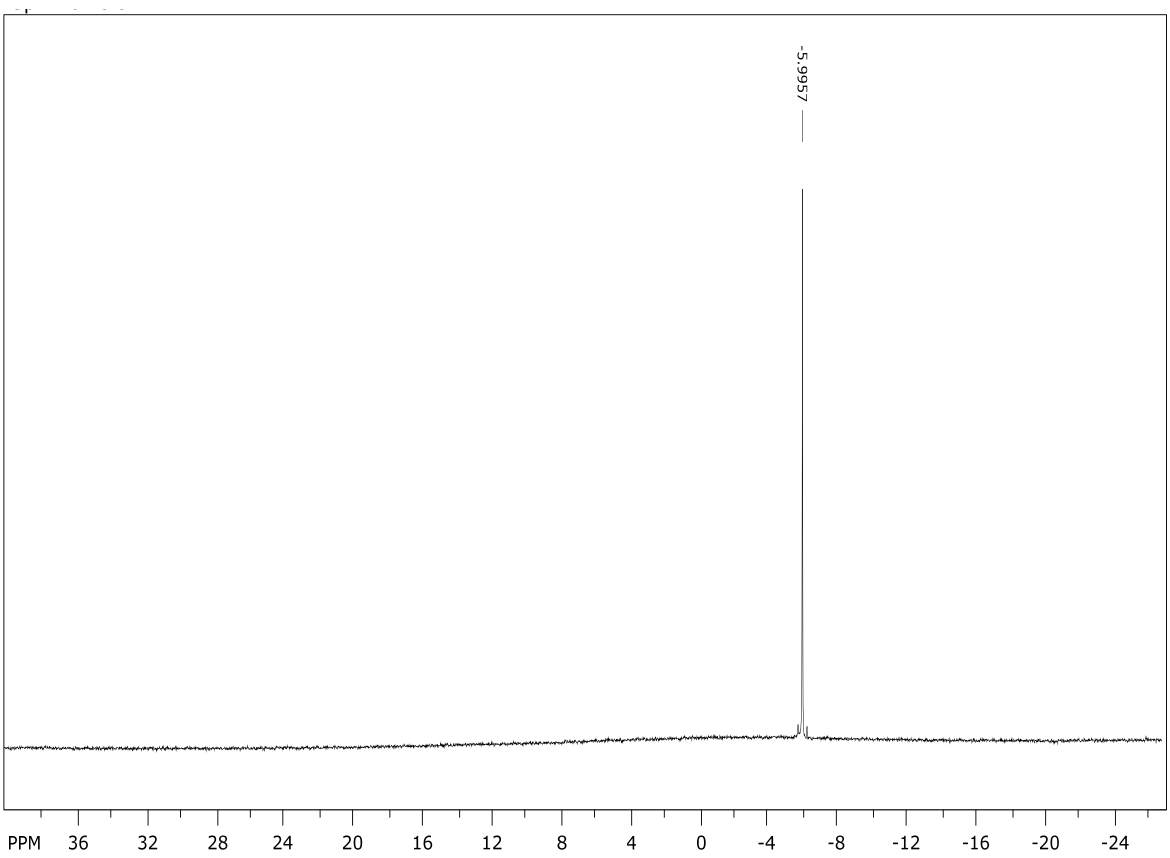

Figure S24. ^{11}B NMR spectrum of [(η^6-*p*-cymene){(IDipp)P}Os(^{Me}IMe)][BAr^F] (**5**) in THF-d_8 at room temperature.

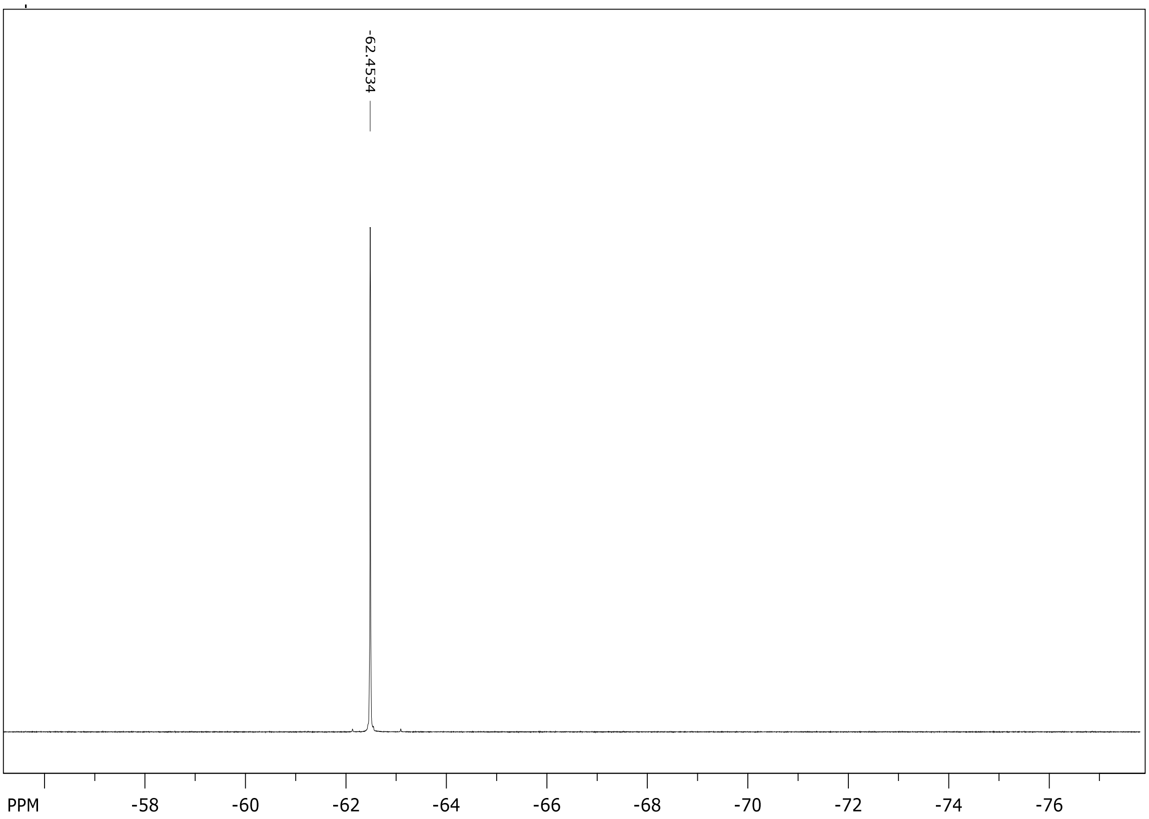

Figure S25. ^{19}F NMR spectrum of [(η^6-*p*-cymene){(IDipp)P}Os(^{Me}IMe)][BAr^F] (**5**) in THF-d_8 at room temperature.

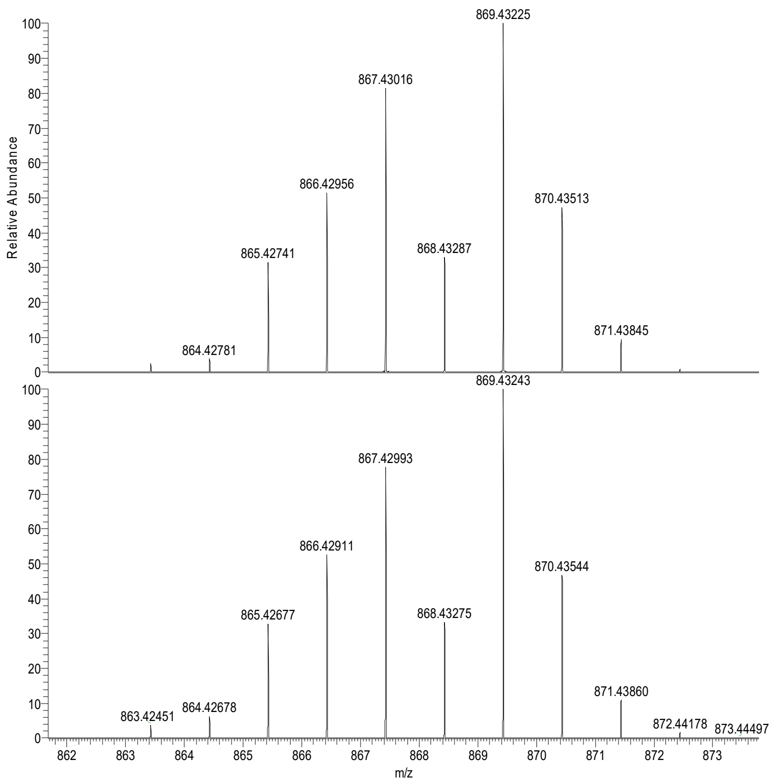

Figure S26. HRMS of the complex [(η^6-*p*-cymene){(IDipp)P}Os(^{Me}IMe)][BAr^F] (**5**) in positive mode, shows the cationic part of the complex (*top*: experimental isotopic pattern, bottom: computed isotopic pattern).

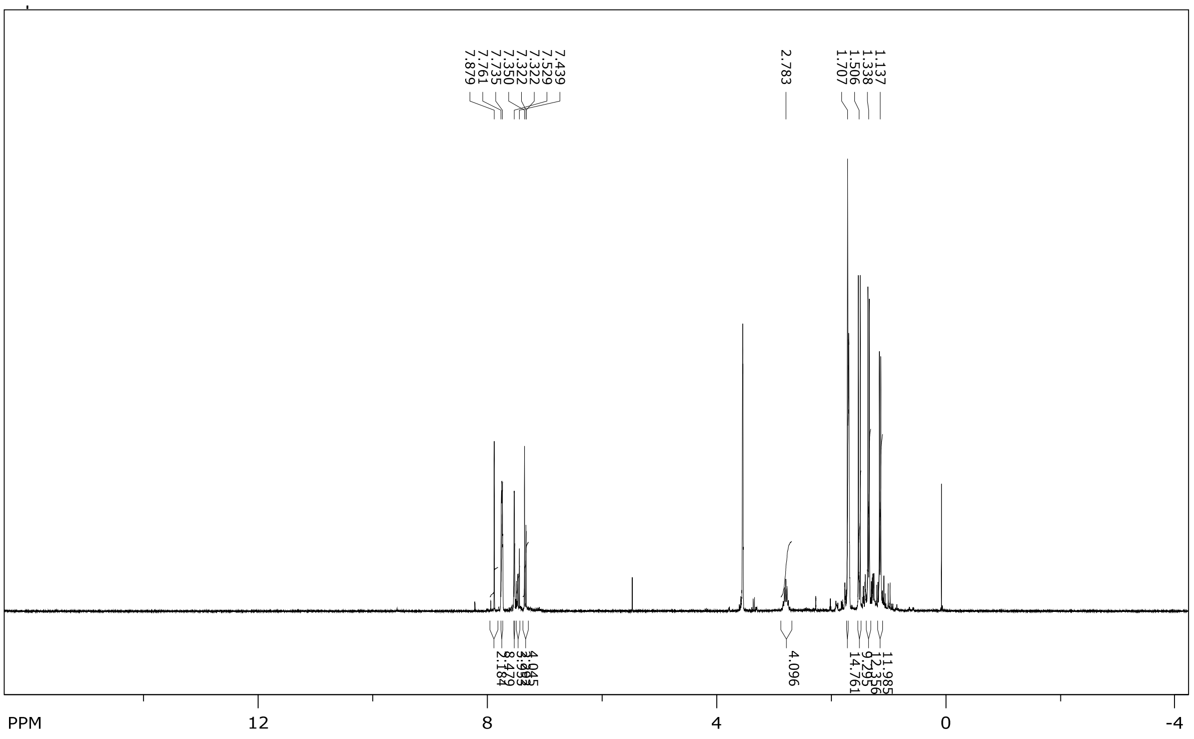

Figure S27. ^{1}H NMR spectrum of complex [(η^5-C_5Me_5){(IDipp)P}Ir(PMe_3)][BAr^F] (**6**) in THF-*d*$_8$ at room temperature.

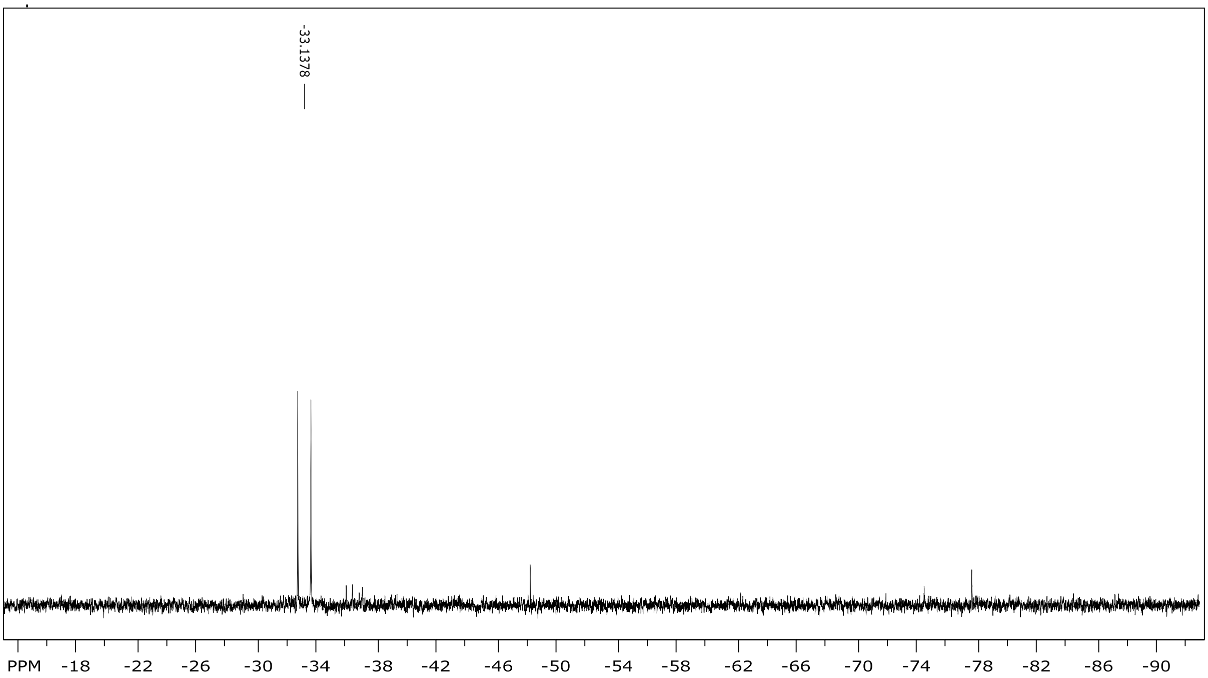

Figure S28. ^{31}P NMR spectrum of [(η^5-C_5Me_5){(IDipp)P}Ir(PMe_3)][BAr^F] (**6**) in THF-d_8 at room temperature.

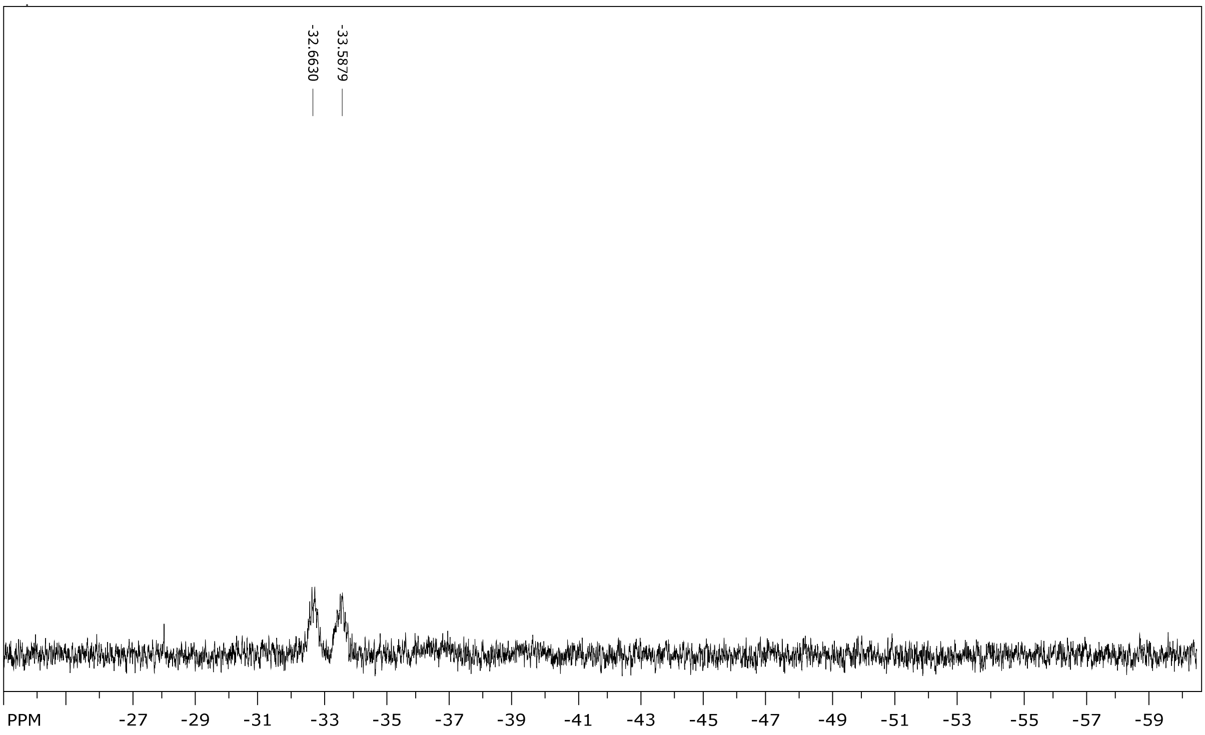

Figure S29. Proton coupled ^{31}P NMR of [(η^5-C_5Me_5){(IDipp)P}Ir(PMe_3)][BAr^F] (**6**) in THF-d_8 at room temperature.

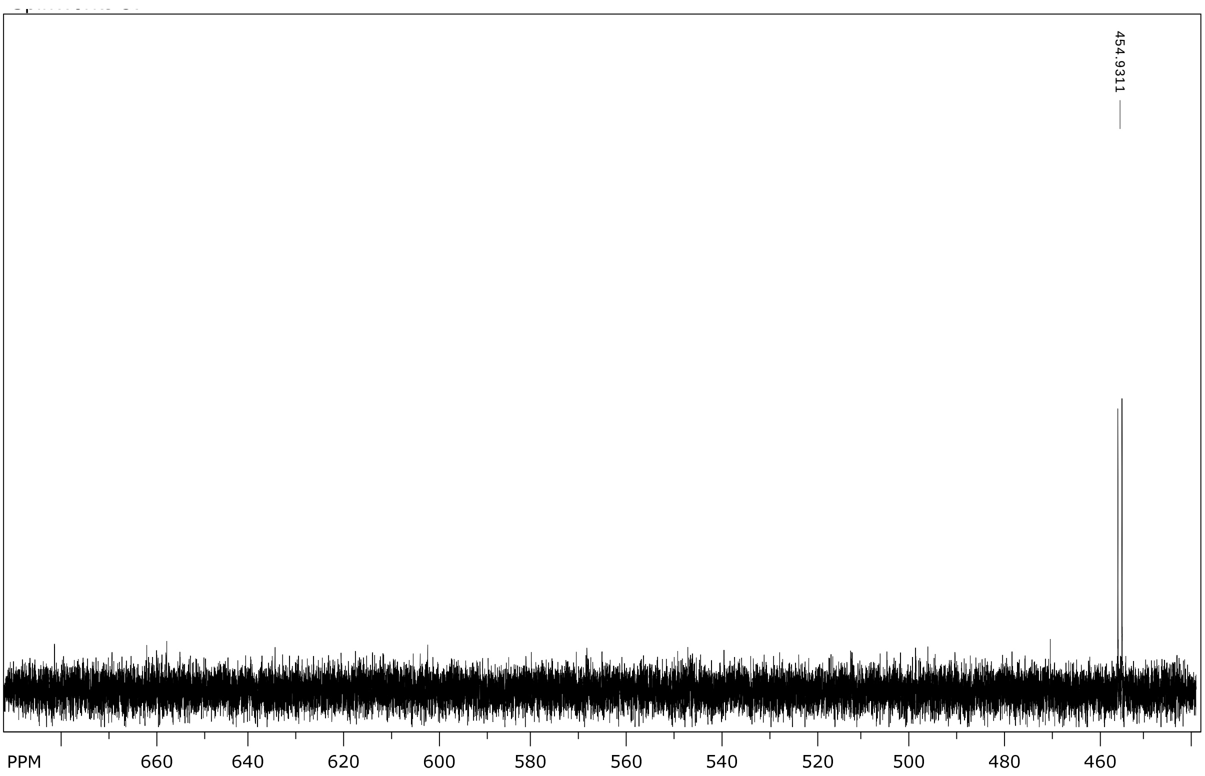

Figure S30. ^{31}P NMR spectrum of $[(\eta^5\text{-}C_5Me_5)\{(IDipp)P\}Ir(PMe_3)][BAr^F]$ (**6**) in THF-d_8 at room temperature.

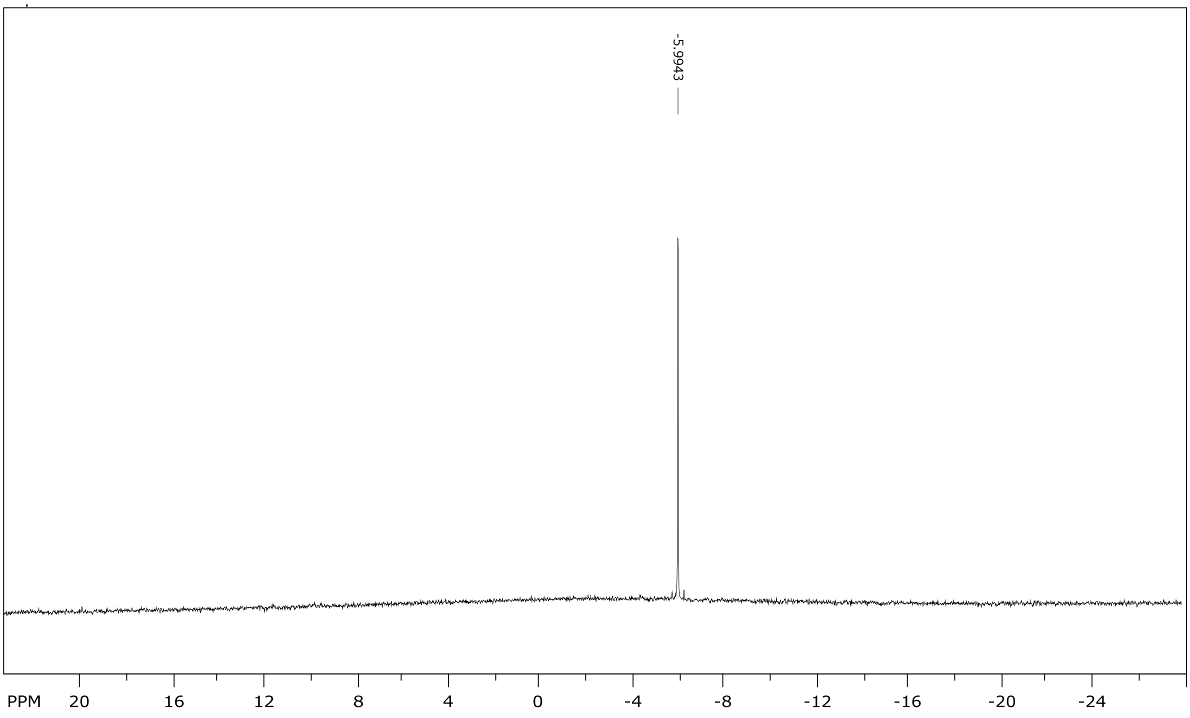

Figure S31. ^{11}B NMR spectrum of $[(\eta^5\text{-}C_5Me_5)\{(IDipp)P\}Ir(PMe_3)][BAr^F]$ (**6**) in THF-d_8 at room temperature.

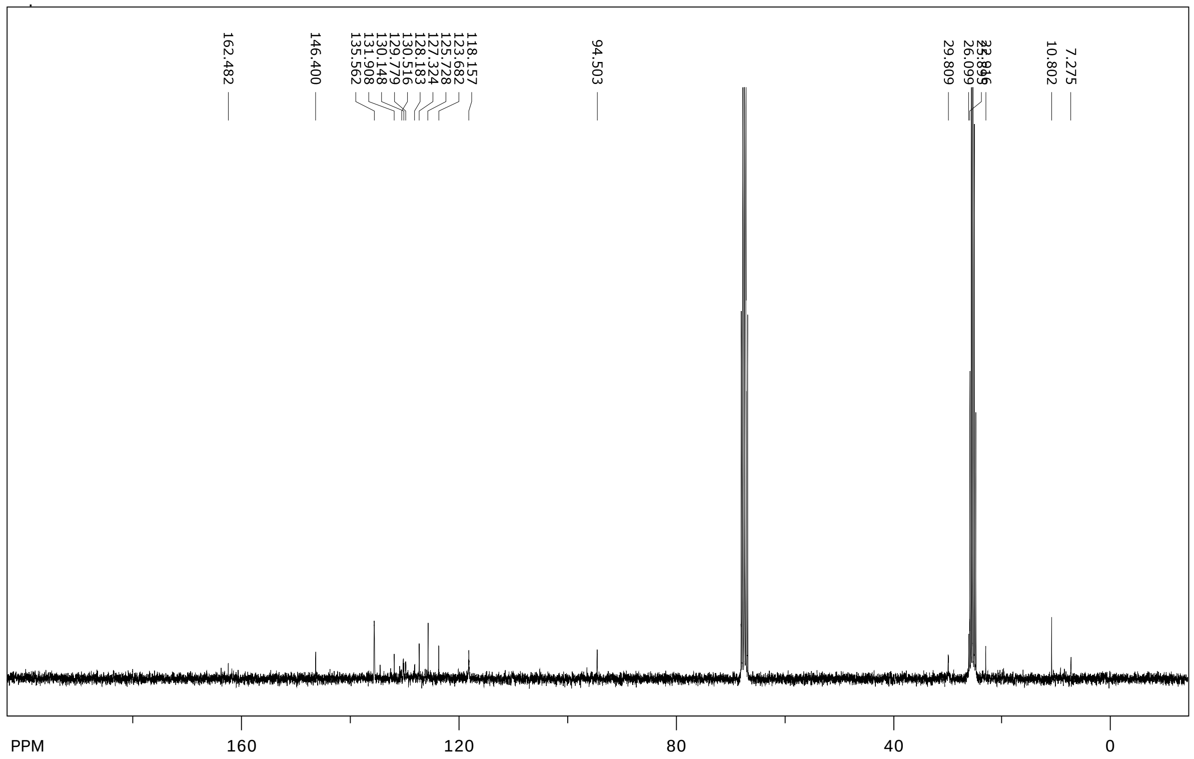

Figure S32. ^{13}C NMR spectrum of [(η^5-C_5Me_5){(IDipp)P}Ir(PMe_3)][BAr^F] (**6**) in THF-d_8 at room temperature.

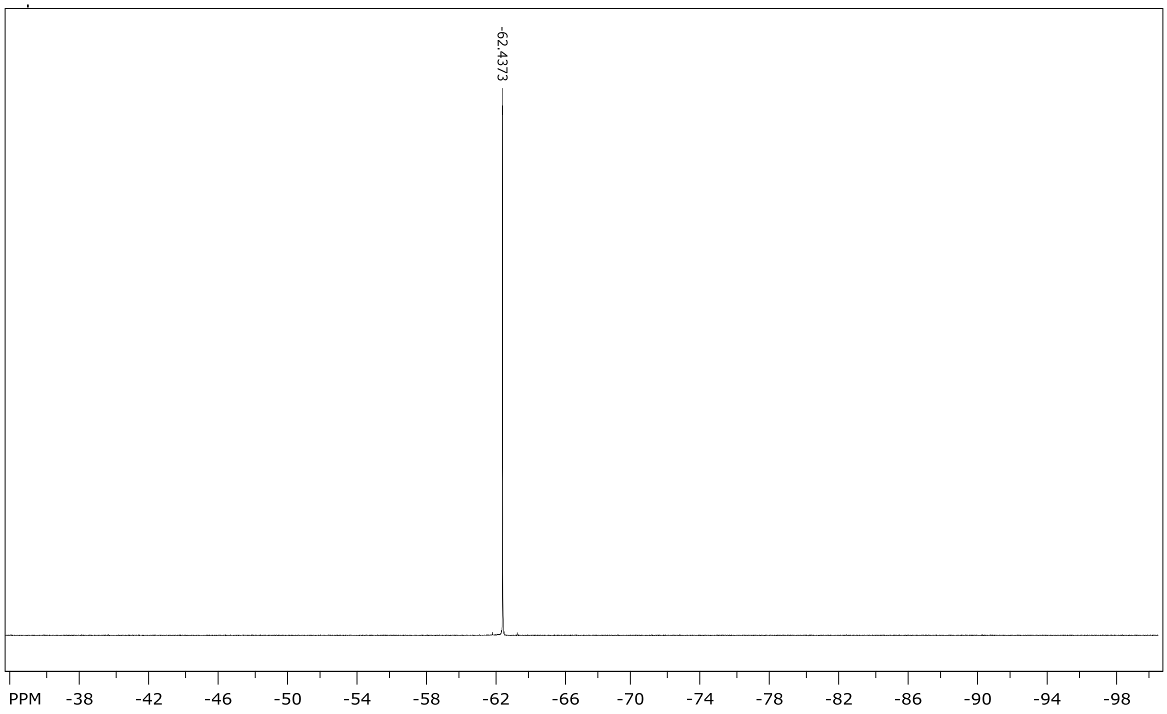

Figure S33. ^{19}F NMR spectrum of [(η^5-C_5Me_5){(IDipp)P}Ir(PMe_3)][BAr^F] (**6**) in THF-d_8 at room temperature.

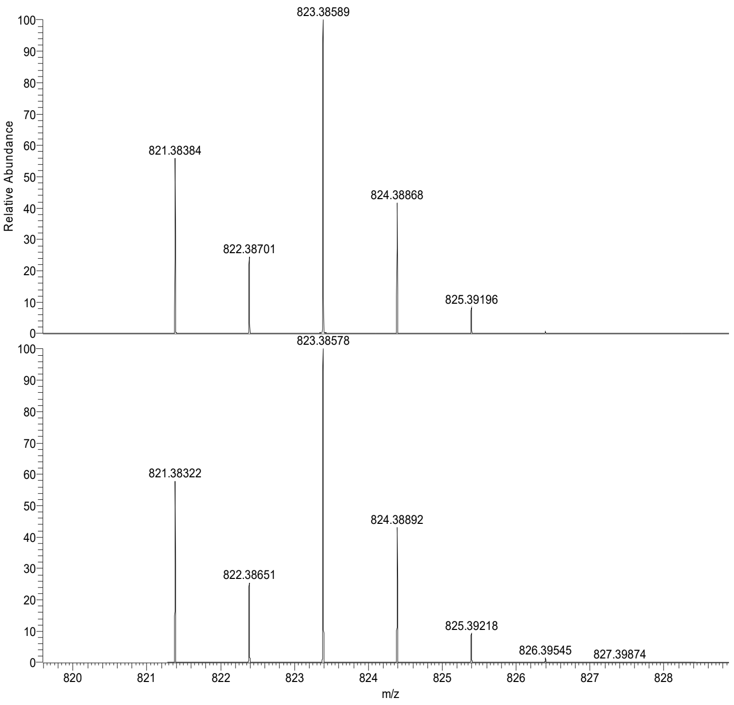

Figure S34. HRMS of the complex [(η^5-C_5Me_5){(IDipp)P}Ir(PMe_3)][BAr^F] (**6**) in positive mode, shows the cationic part of the complex (top: experimental isotopic pattern, bottom: computed isotopic pattern).

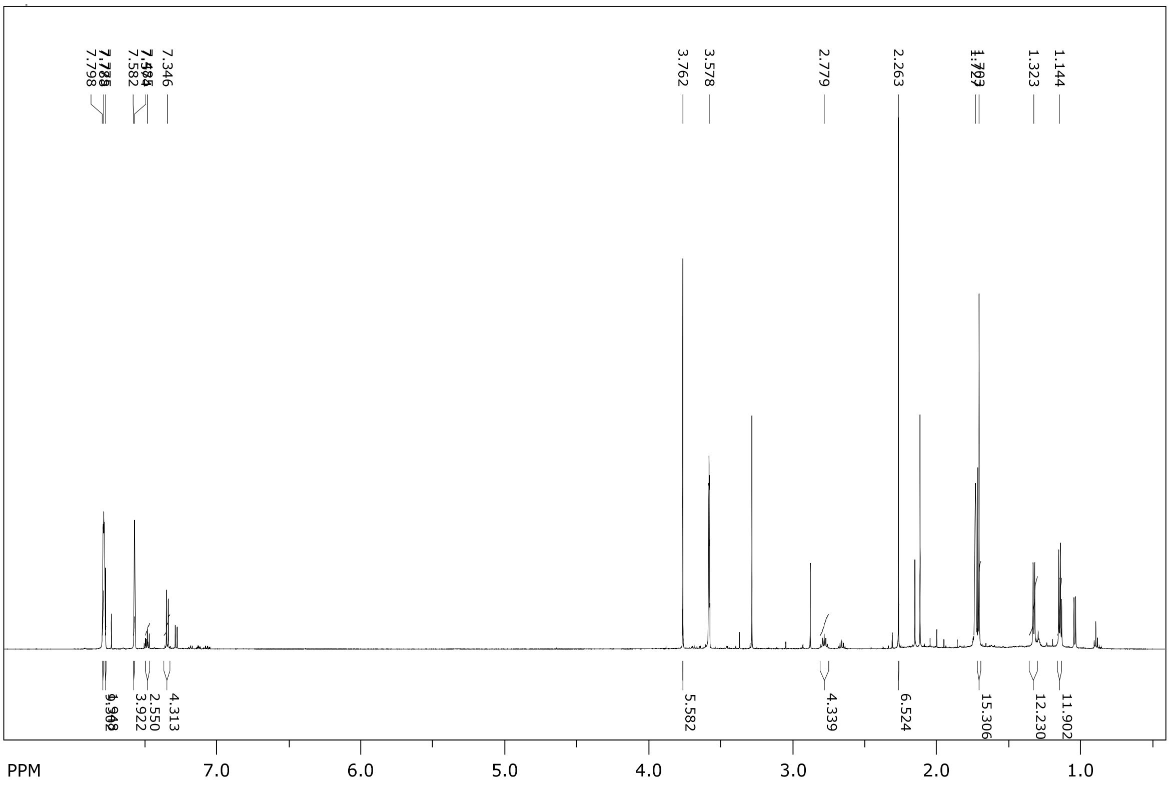

Figure S35. ^{1}H NMR spectrum of the *E*/*Z* mixture of [(η^5-C_5Me_5){(IDipp)P}Ir(^{Me}IMe)][BAr^F] (**7**, *E*-isomer) containing both isomers. Chemical shifts assigned for the *trans* isomer.

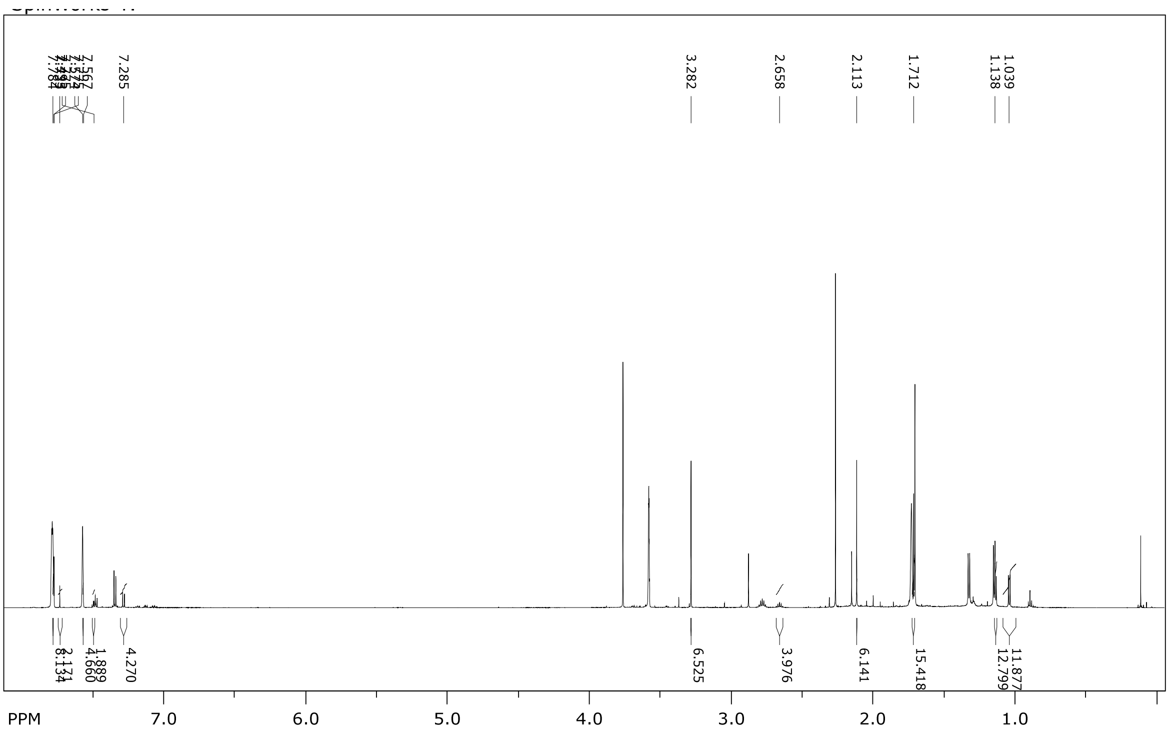

Figure S36. ^{1}H NMR spectrum of the *E*/*Z* mixture of [(η^5-C_5Me_5){(IDipp)P}Ir(^{Me}IMe)][BAr^F] (**7**, *Z*-isomer) containing both isomers. Chemical shifts assigned for the *cis* isomer.

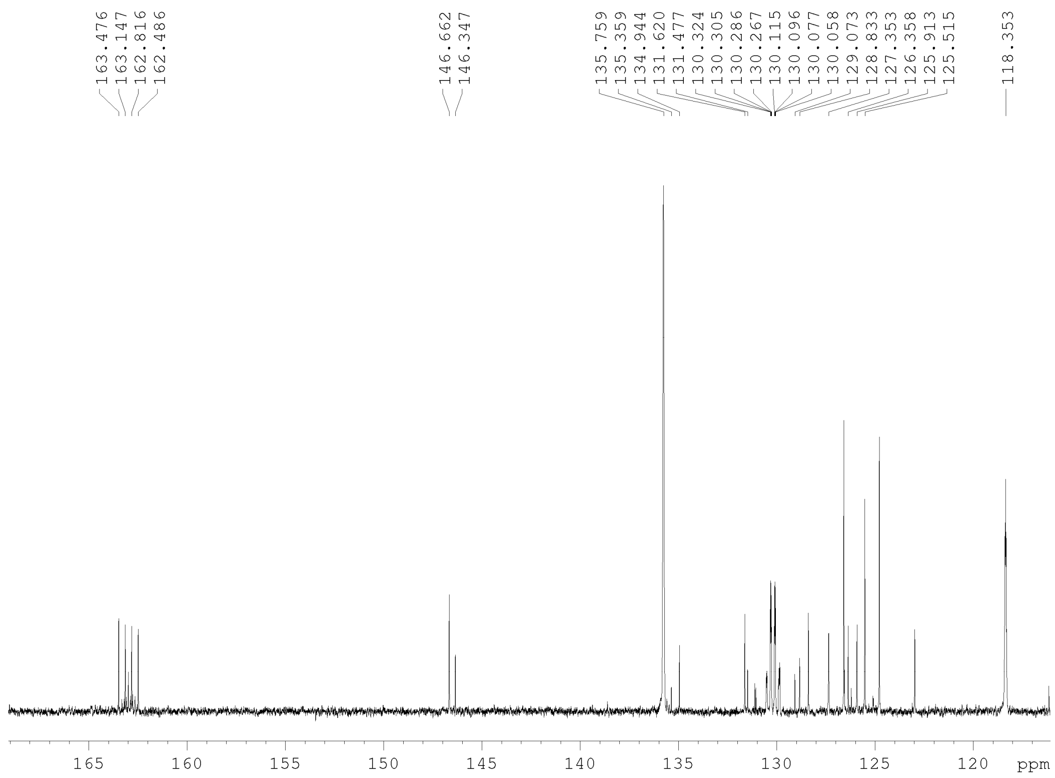

Figure S37. ^{13}C-NMR spectrum of the *E*/*Z* mixture of [(η^5-C_5Me_5){(IDipp)P}Ir(^{Me}IMe)][BAr^F] (**7**) in THF-*d*$_8$ at room temperature.

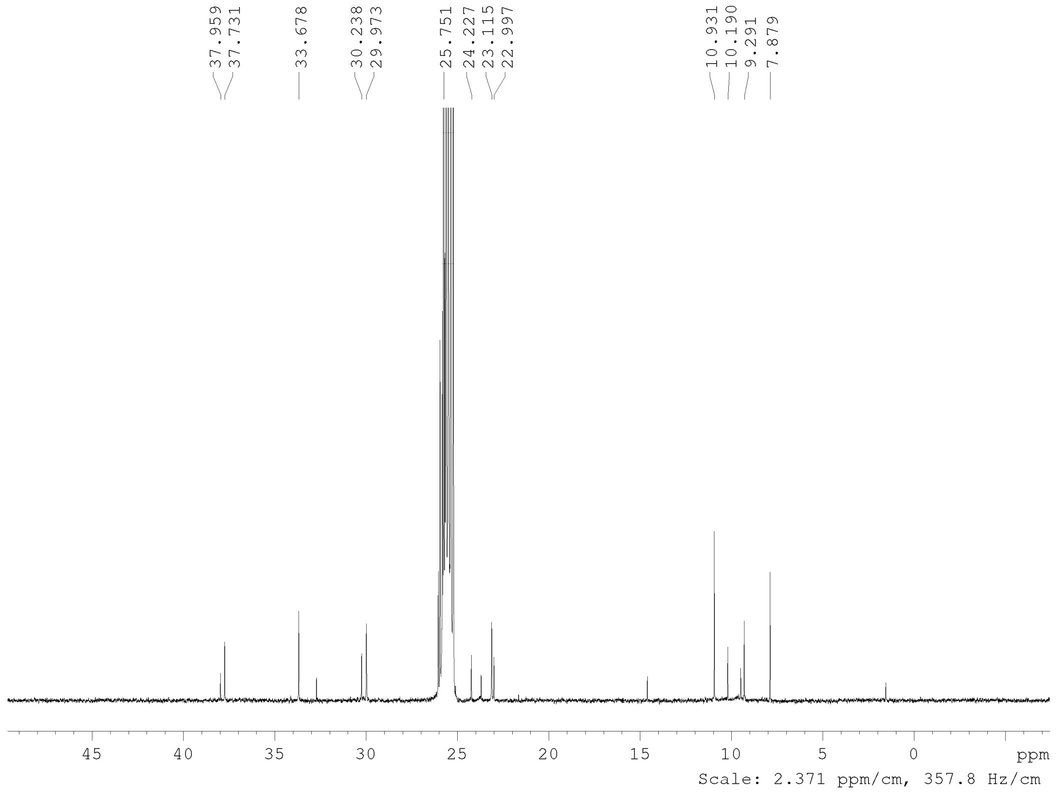

Figure S38. ^{13}C-NMR spectrum of the *E*/*Z* mixture of [(η^5-C_5Me_5){(IDipp)P}Ir(^{Me}IMe)][BAr^F] (**7**) in THF-d_8 at room temperature (expanded region).

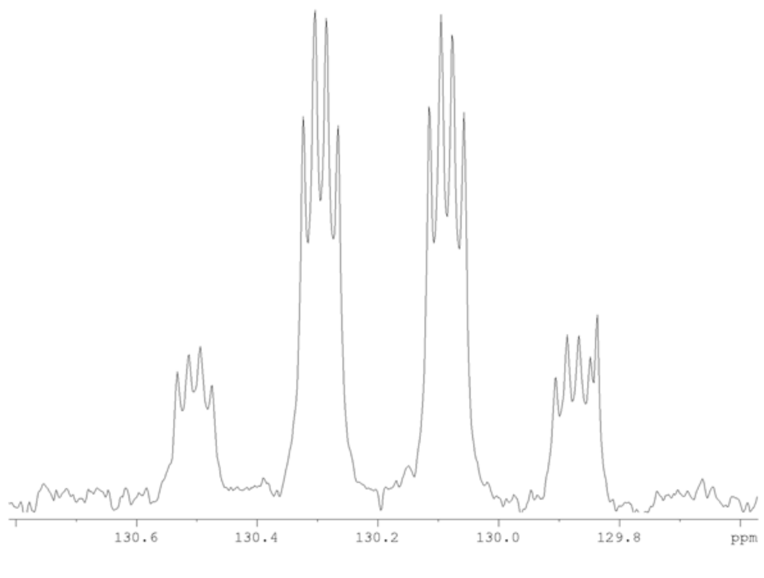

Figure S39. ^{13}C-NMR spectrum of the *E*/*Z* mixture of [(η^5-C_5Me_5){(IDipp)P}Ir(^{Me}IMe)][BAr^F] (**7**) in THF-d_8 at room temperature (expanded region).

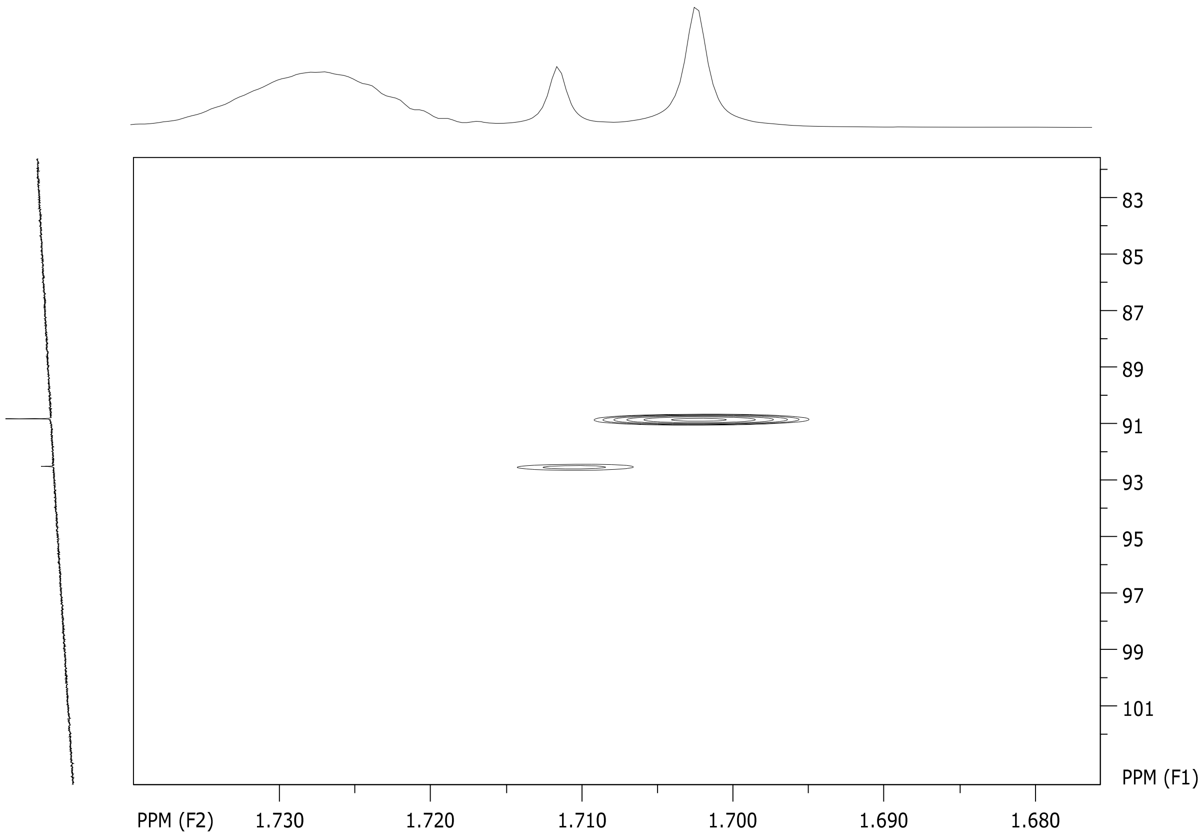

Figure S40. HSQC NMR spectrum of [(η^5-C_5Me_5){(IDipp)P}Ir(^{Me}IMe)][BAr^F] (**7**) in THF-d_8 at room temperature (expanded).

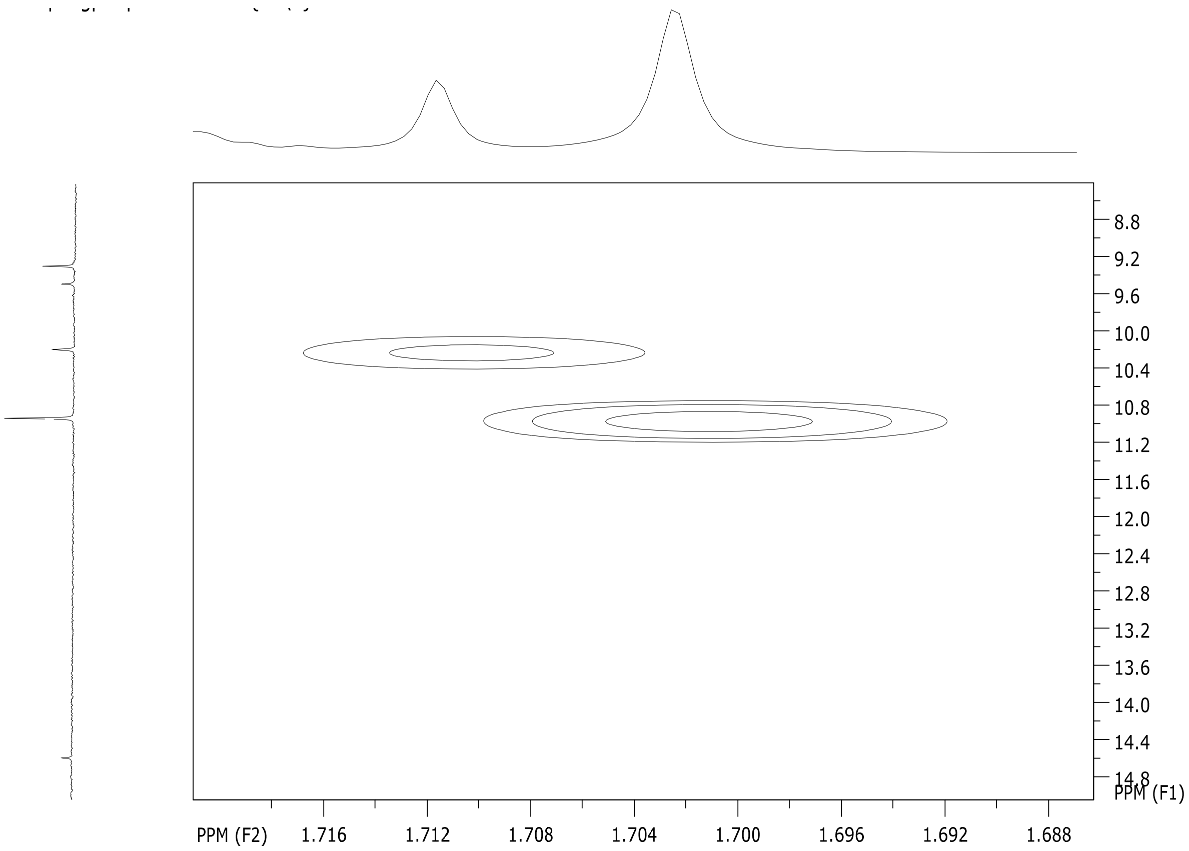

Figure S41. HSQC NMR spectrum of [(η^5-C_5Me_5){(IDipp)P}Ir(^{Me}IMe)][BAr^F] (**7**) (expanded).

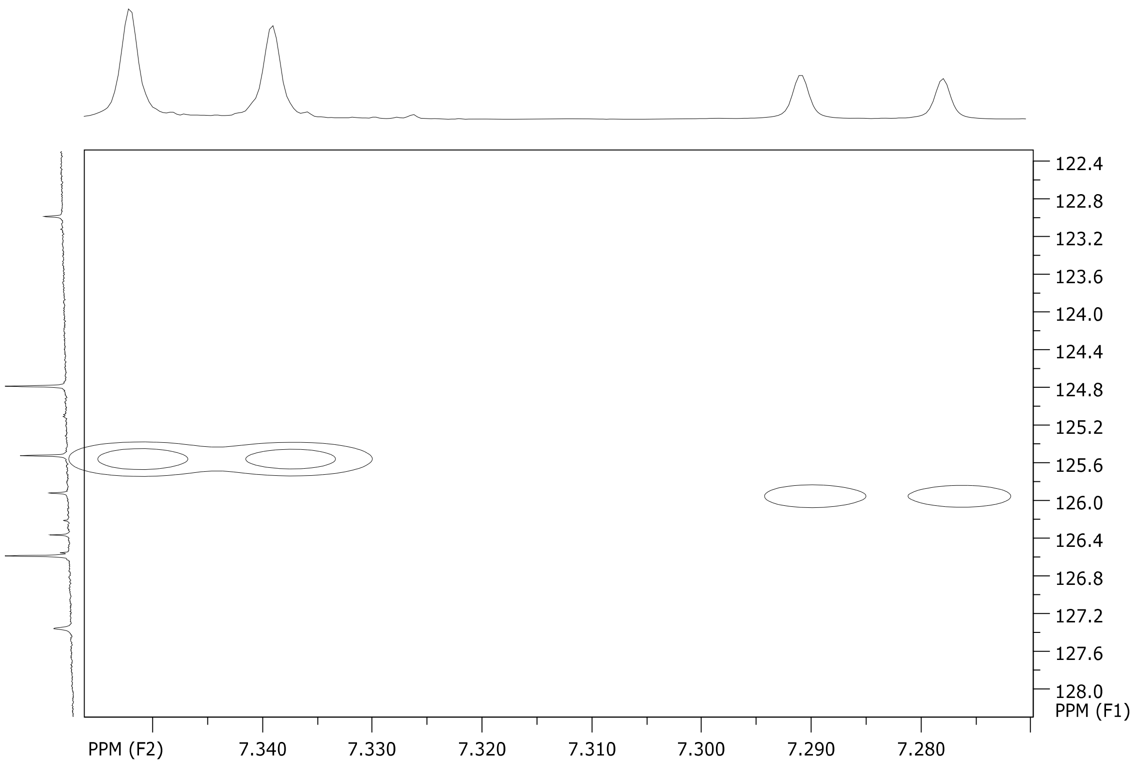

Figure S42. HSQC NMR spectrum of [(η^5-C_5Me_5){(IDipp)P}Ir(^{Me}IMe)][BAr^F] (**7**) containing both isomers (expanded).

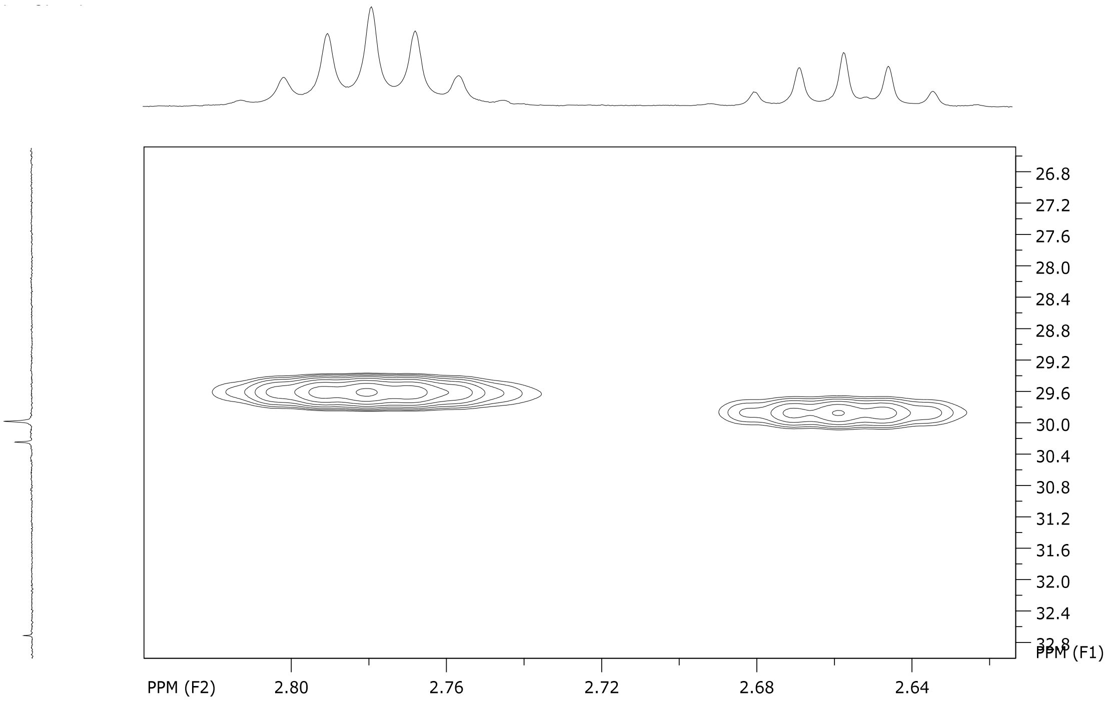

Figure S43. HSQC NMR spectrum of [(η^5-C_5Me_5){(IDipp)P}Ir(^{Me}IMe)][BAr^F] (**7**) (expanded).

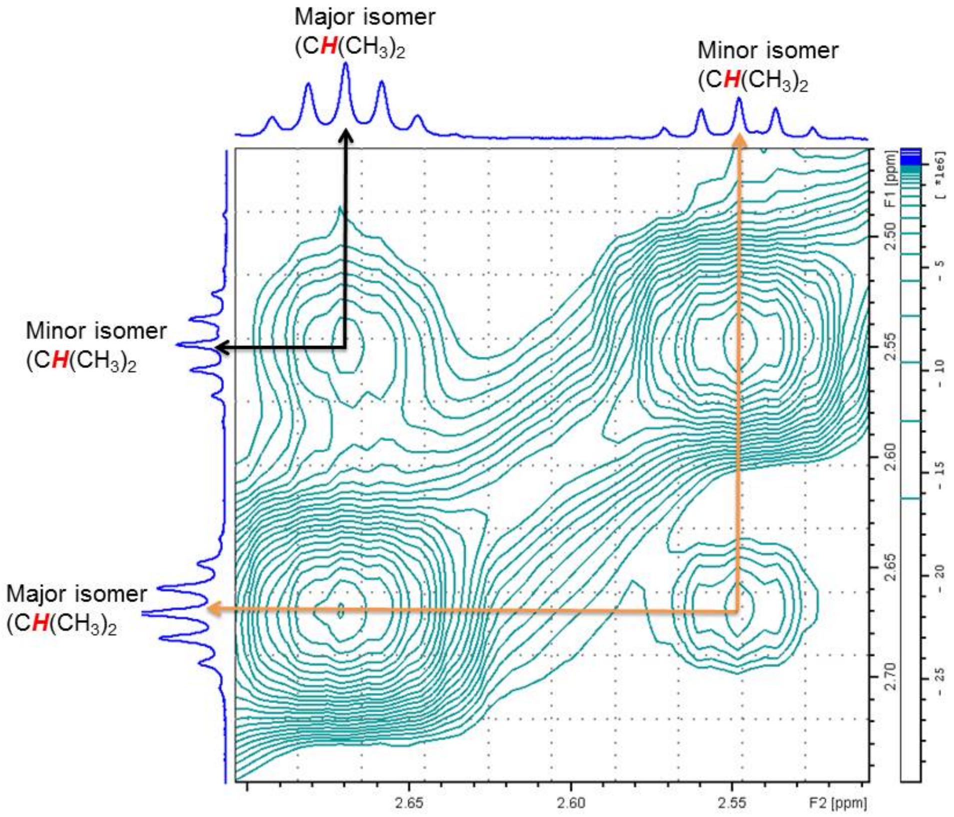

Figure S44. NOESY cross peaks of the isopropyl protons in the major (*E*) and minor (*Z*) isomers of the complex [(η^5-C_5Me_5){(IDipp)P}Ir(^{Me}IMe)][BAr^F] (**7**) (mixing time; d8: 0.5 seconds).

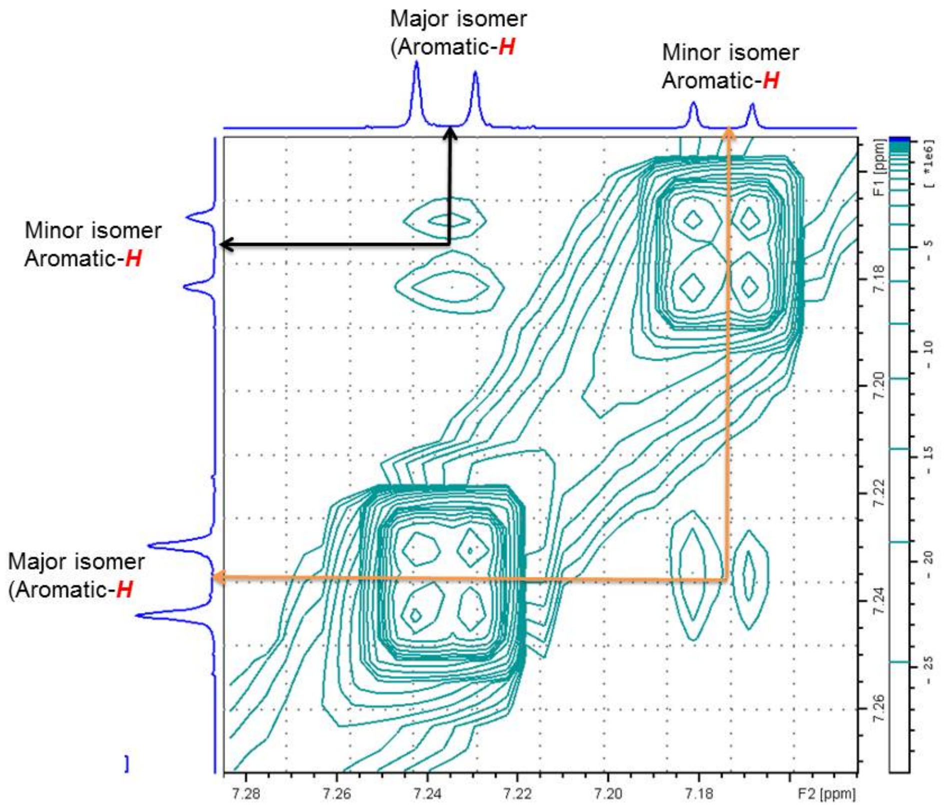

Figure S45. NOESY cross speaks of the aromatic protons in the major (*E*) and minor (*Z*) isomers of the complex [(η^5-C_5Me_5){(IDipp)P}Ir(^{Me}IMe)][BAr^F] (**7**) (mixing time; d8:0.5 seconds).

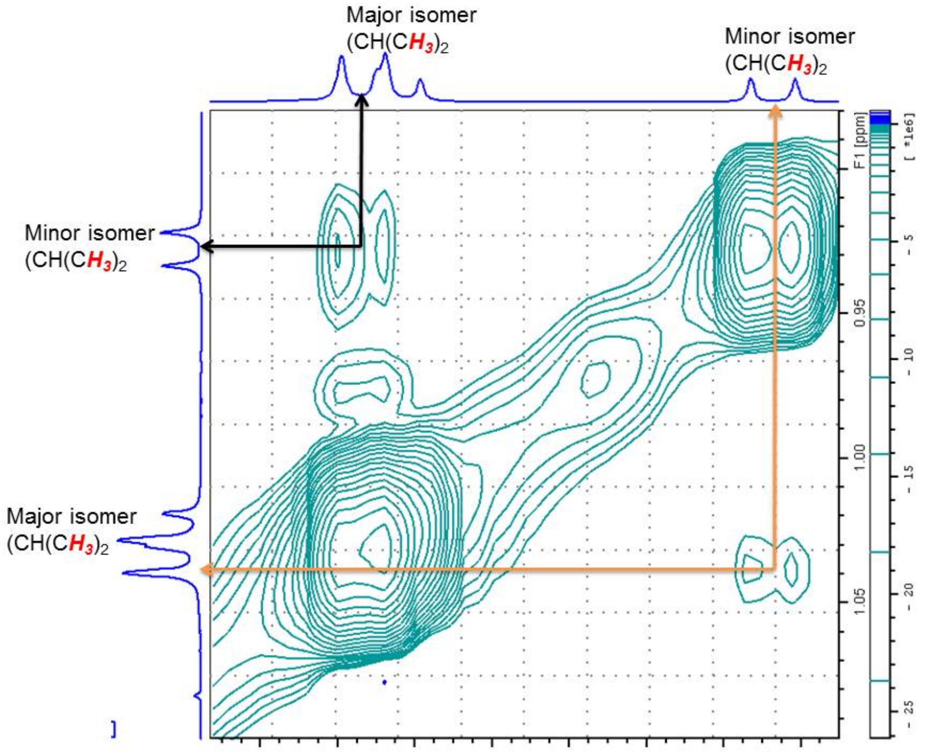

Figure S46. NOESY cross speaks of the methyl protons in the major (*E*) and minor (*Z*) isomers of the complex [(η^5-C_5Me_5){(IDipp)P}Ir(^{Me}IMe)][BAr^F] (**7**) (mixing time; d8:0.5 seconds).

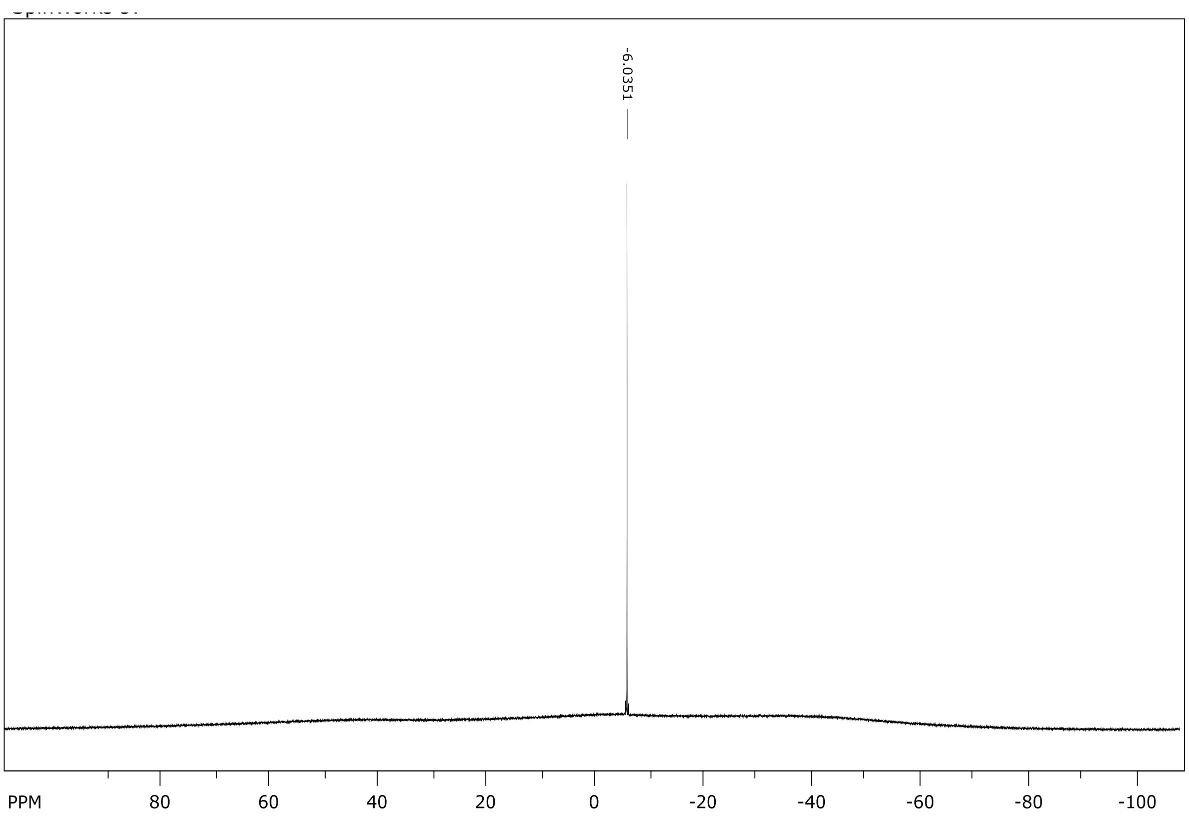

Figure S47. ^{11}B NMR spectrum of [(η^5-C_5Me_5){(IDipp)P}Ir(^{Me}IMe)][BAr^F] (**7**) in THF-d_8 at room temperature.

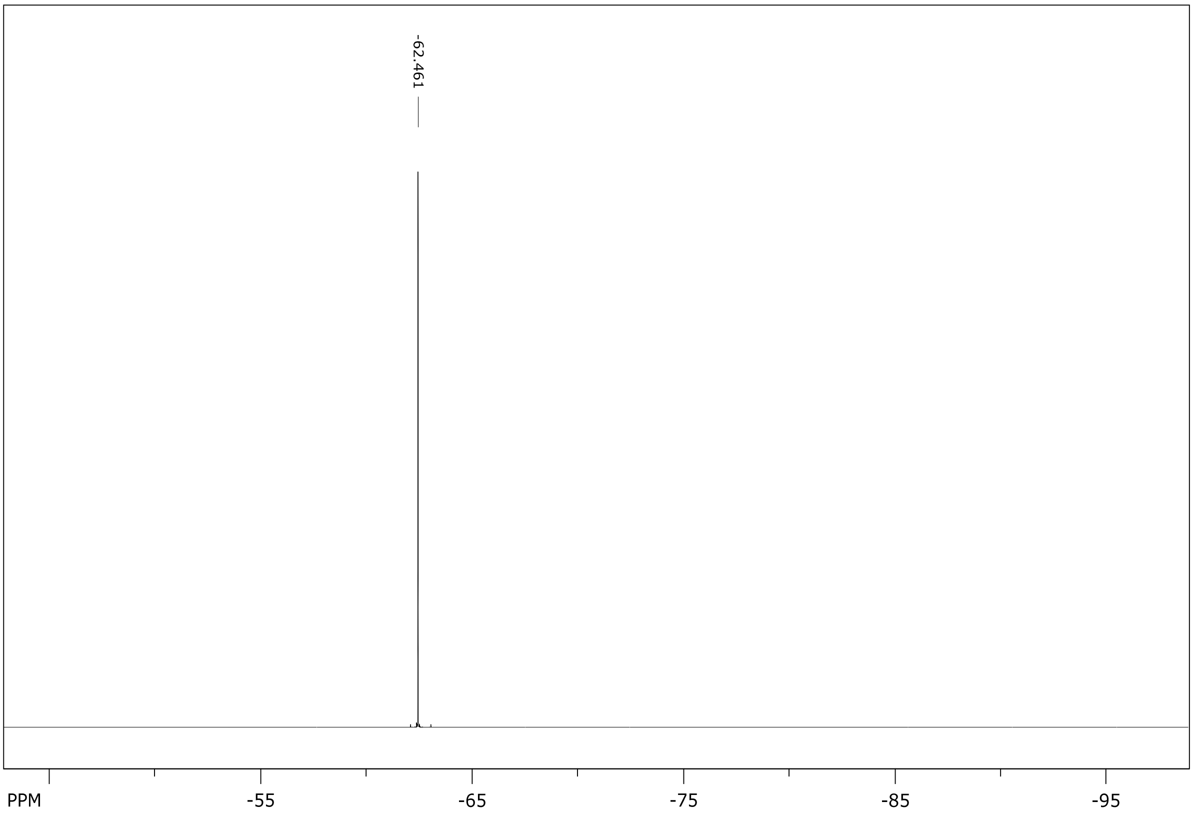

Figure S48. ^{19}F NMR spectrum of $[(\eta^5\text{-}C_5Me_5)\{(IDipp)P\}Ir(^{Me}IMe)][BAr^F]$ (**7**) in THF-d_8 at room temperature.

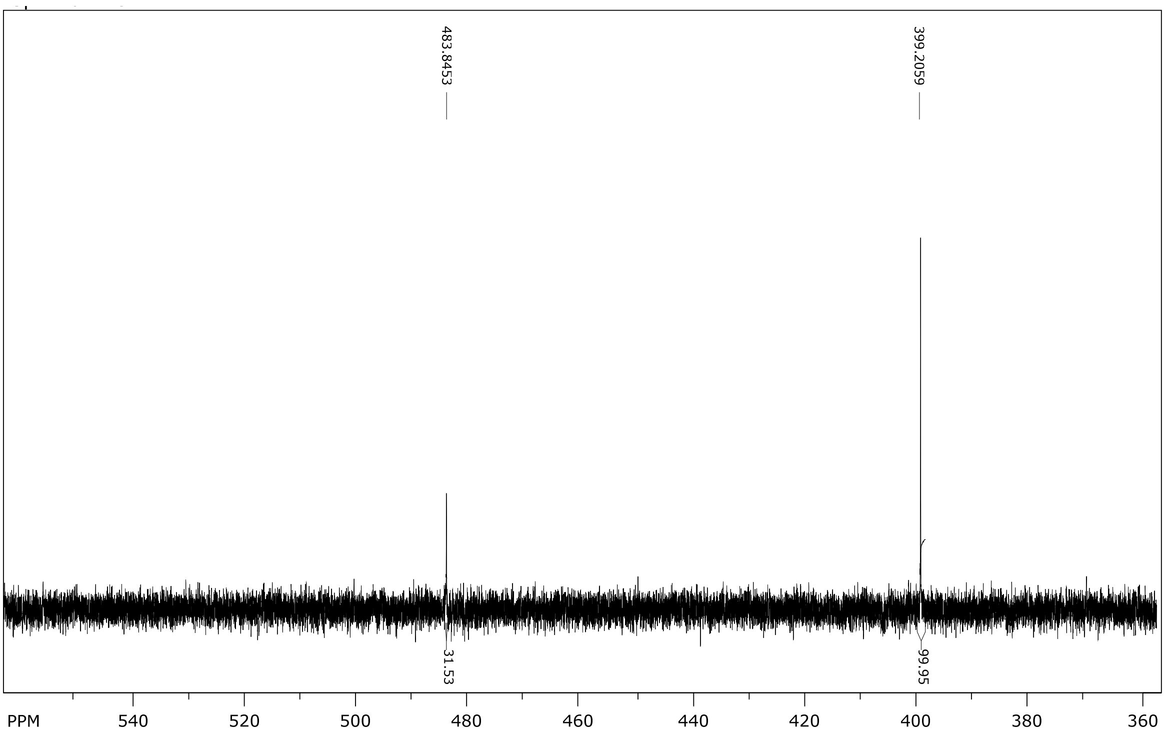

Figure S49. $^{31}P\ \{^1H\}$ NMR spectrum of $[(\eta^5\text{-}C_5Me_5)\{(IDipp)P\}Ir(^{Me}IMe)][BAr^F]$ (**7**) in THF-d_8 at room temperature.

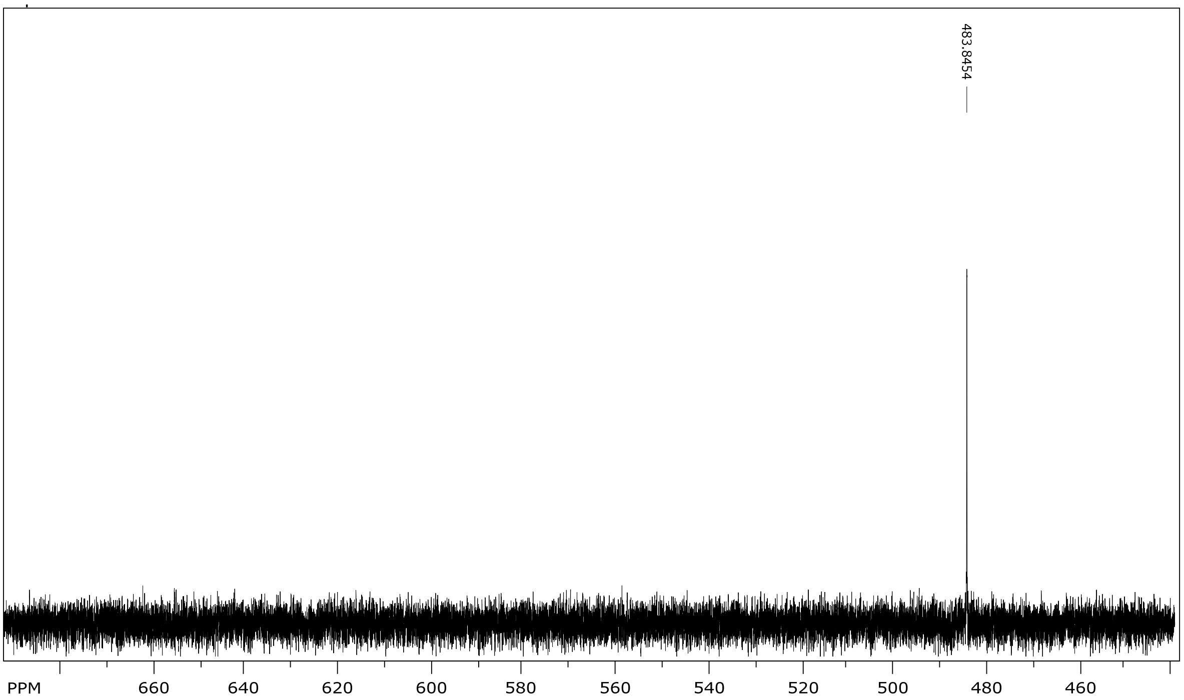

Figure S50. ^{31}P {^{1}H} NMR spectrum of [(η^{5}-C_5Me_5){(IDipp)P}Ir(^{Me}IMe)][BAr^{F}] (**7**) in THF-d_8 at room temperature.

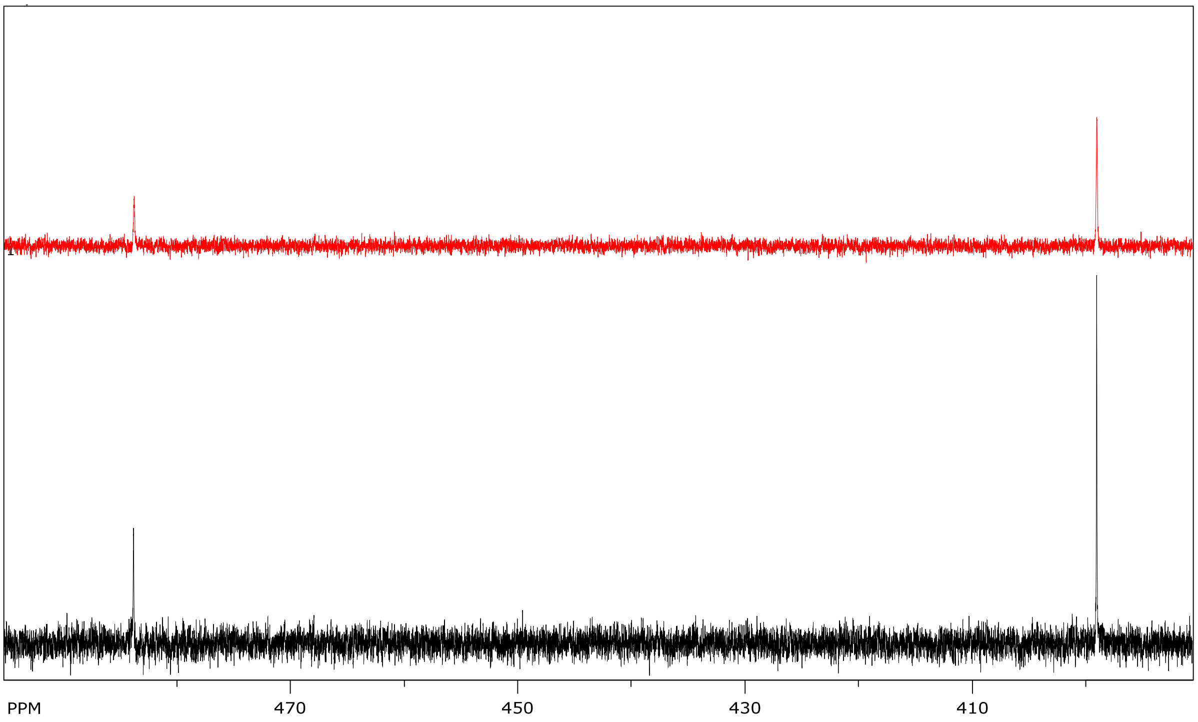

Figure S51. ^{31}P NMR spectrum of [(η^{5}-C_5Me_5){(IDipp)P}Ir(^{Me}IMe)][BAr^{F}] (**7**) (Red: Proton coupled ^{31}P NMR spectrum, Black: Proton decoupled ^{31}P NMR spectrum).

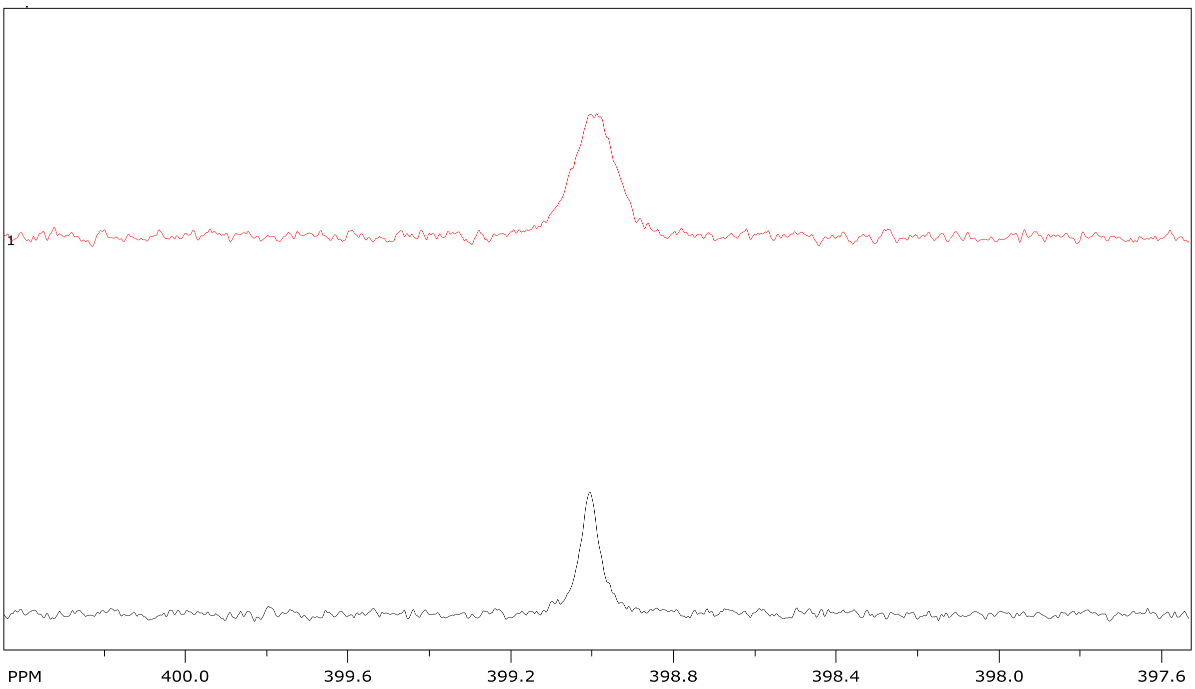

Figure S52. Expanded ^{31}P NMR spectrum of [(η^5-C_5Me_5){(IDipp)P}Ir(^{Me}IMe)][BAr^F] (**7**) (Red: Proton coupled ^{31}P NMR, Black: Proton decoupled ^{31}P NMR spectrum).

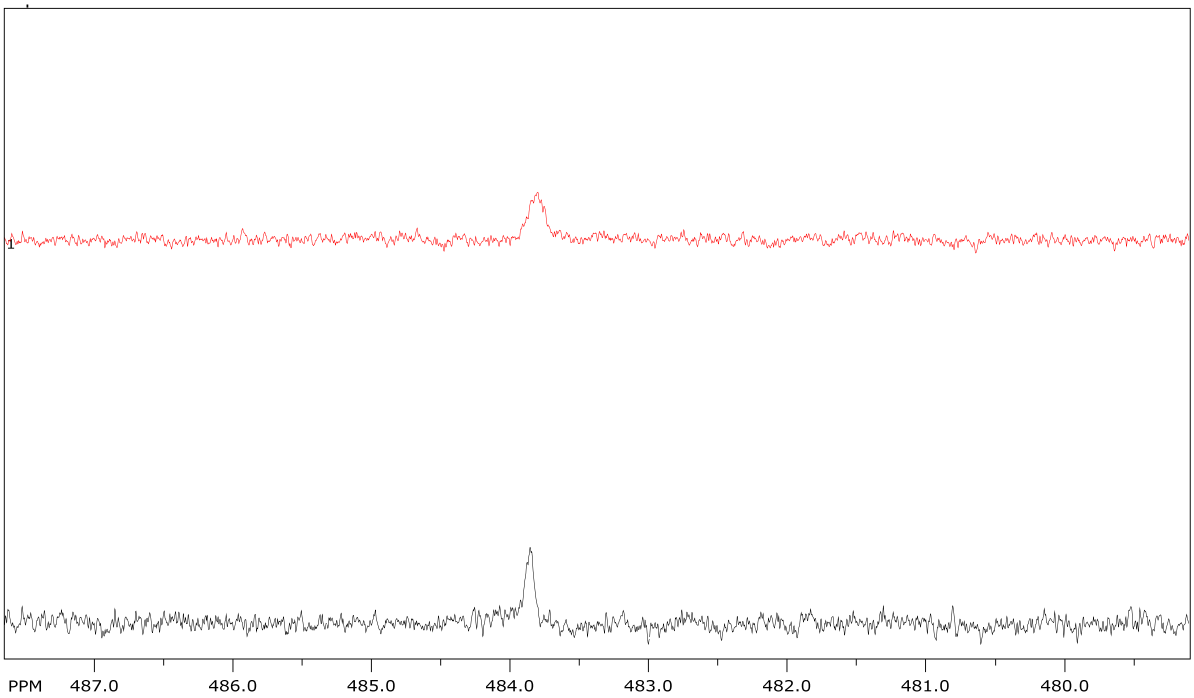

Figure S53. Expanded ^{31}P NMR spectrum of [(η^5-C_5Me_5){(IDipp)P}Ir(^{Me}IMe)][BAr^F] (**7**) (Red: Proton coupled ^{31}P NMR, Black: Proton decoupled ^{31}P NMR).

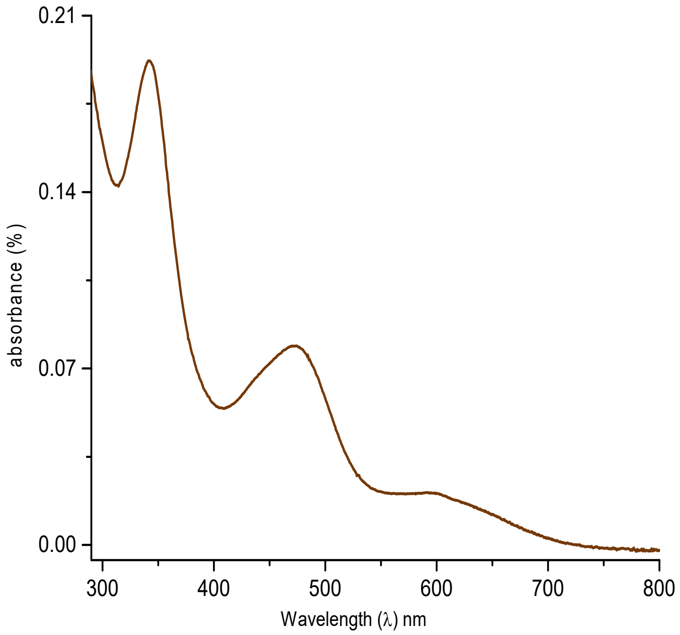

Figure S54. UV-Vis spectrum (in THF) of [(η^5-C_5Me_5){(IDipp)P}Ir(^{Me}IMe)][BAr^F] (**7**).

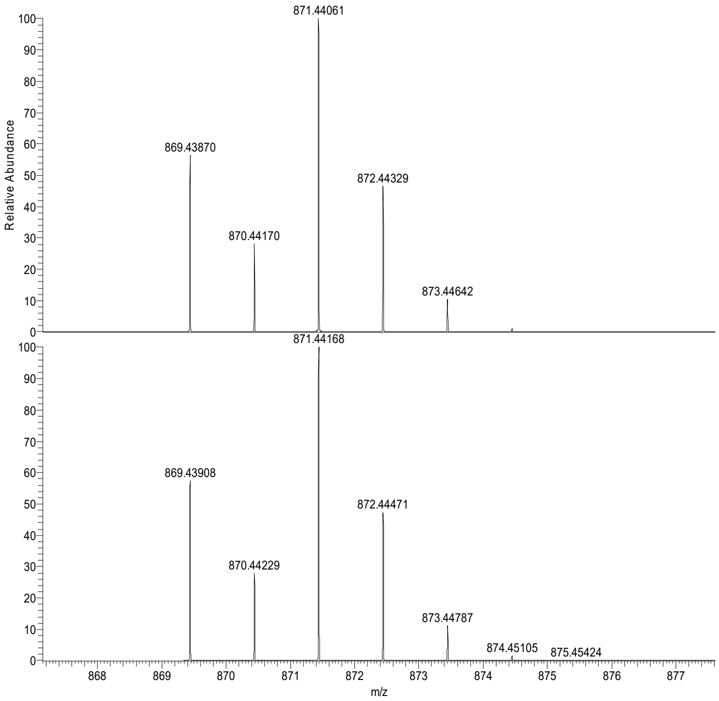

Figure S55. HRMS (ESI-positive mode) of the complex [(η^5-C_5Me_5){(IDipp)P}Ir(^{Me}IMe)][BAr^F] (**7**) shows the cationic part of the complex (top: experimental isotopic pattern, bottom: computed isotopic pattern).

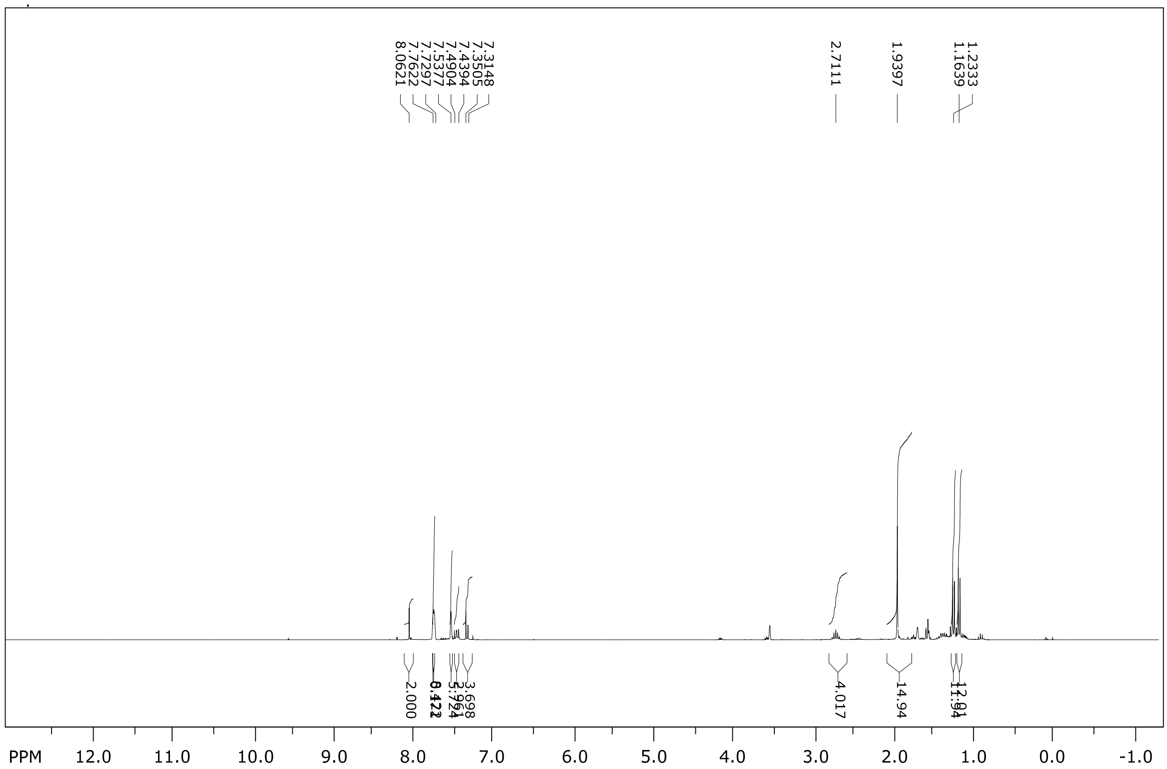

Figure S56. ^{1}H NMR spectrum of $[(\eta^5\text{-}C_5Me_5)\{(IDipp)P\}Ir(CO)][BAr^F]$ (**8**) in THF-d_8 at room temperature.

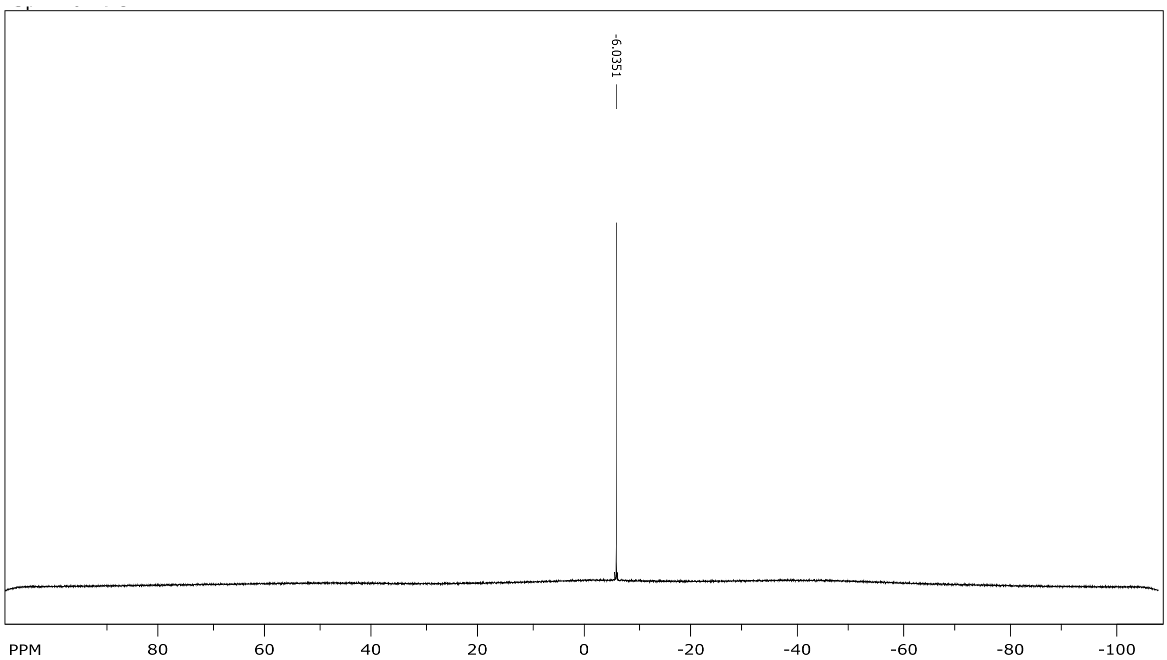

Figure S57. ^{11}B NMR spectrum of $[(\eta^5\text{-}C_5Me_5)\{(IDipp)P\}Ir(CO)][BAr^F]$ (**8**) in THF-d_8 at room temperature.

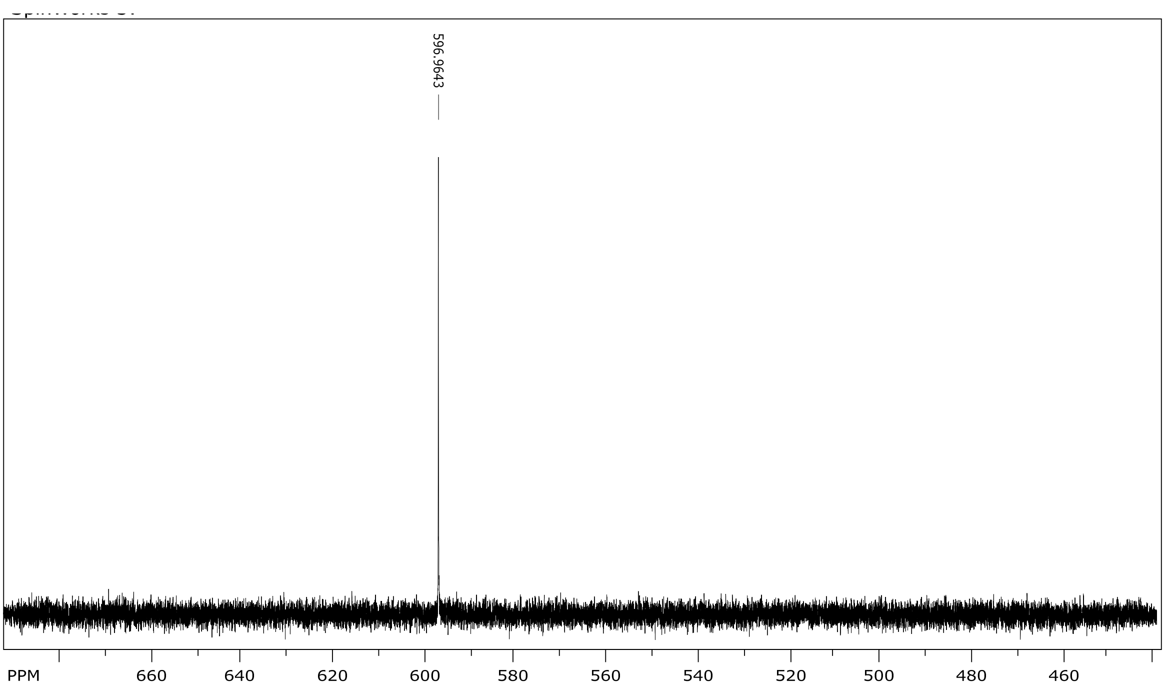

Figure S58. ^{31}P NMR spectrum of $[(\eta^5\text{-}C_5Me_5)\{(IDipp)P\}Ir(CO)][BAr^F]$ (**8**) in THF-d_8 at room temperature.

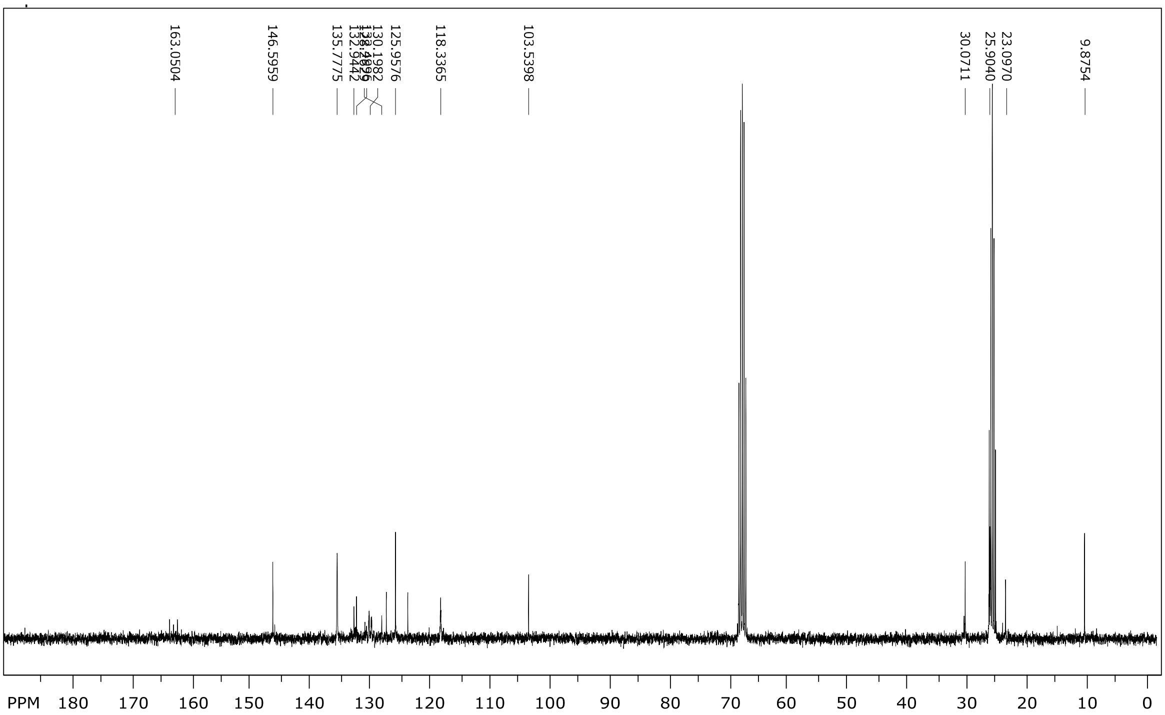

Figure S59. ^{13}C-NMR spectrum of $[(\eta^5\text{-}C_5Me_5)\{(IDipp)P\}Ir(CO)][BAr^F]$ (**8**) in THF-d_8 at room temperature.

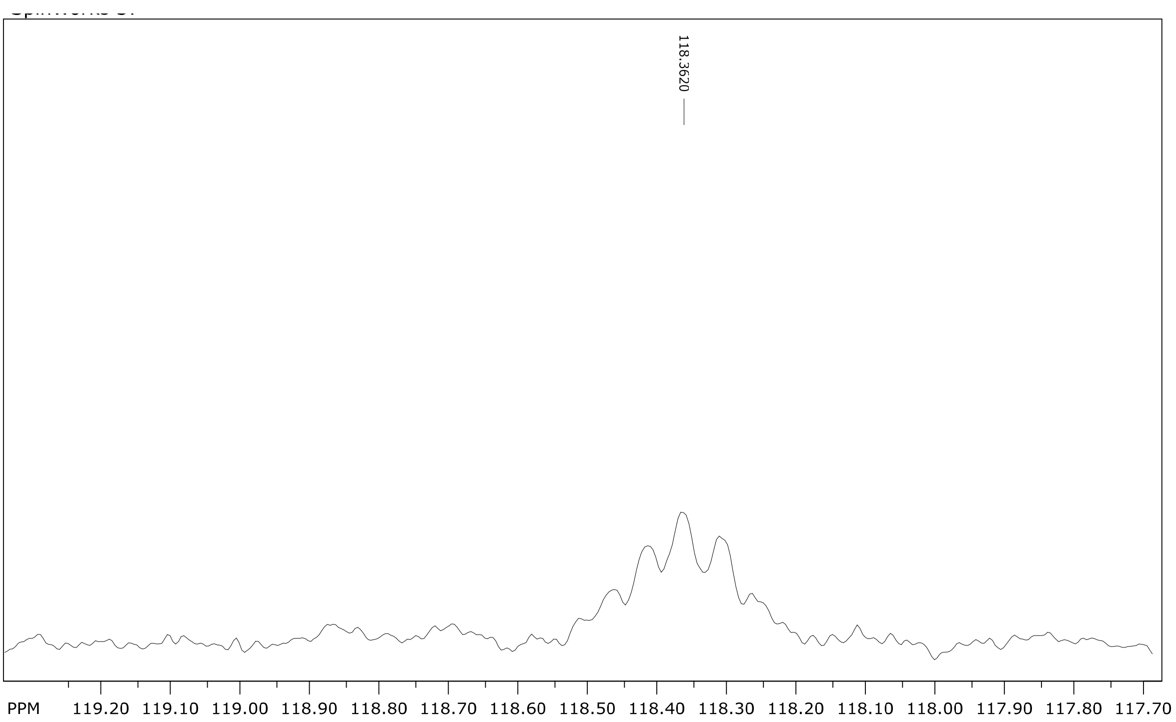

Figure S60. ^{13}C-NMR spectrum (expanded region) of [(η^5-C_5Me_5){(IDipp)P}Ir(CO)][BAr^F] (**8**) in THF-d_8 at room temperature.

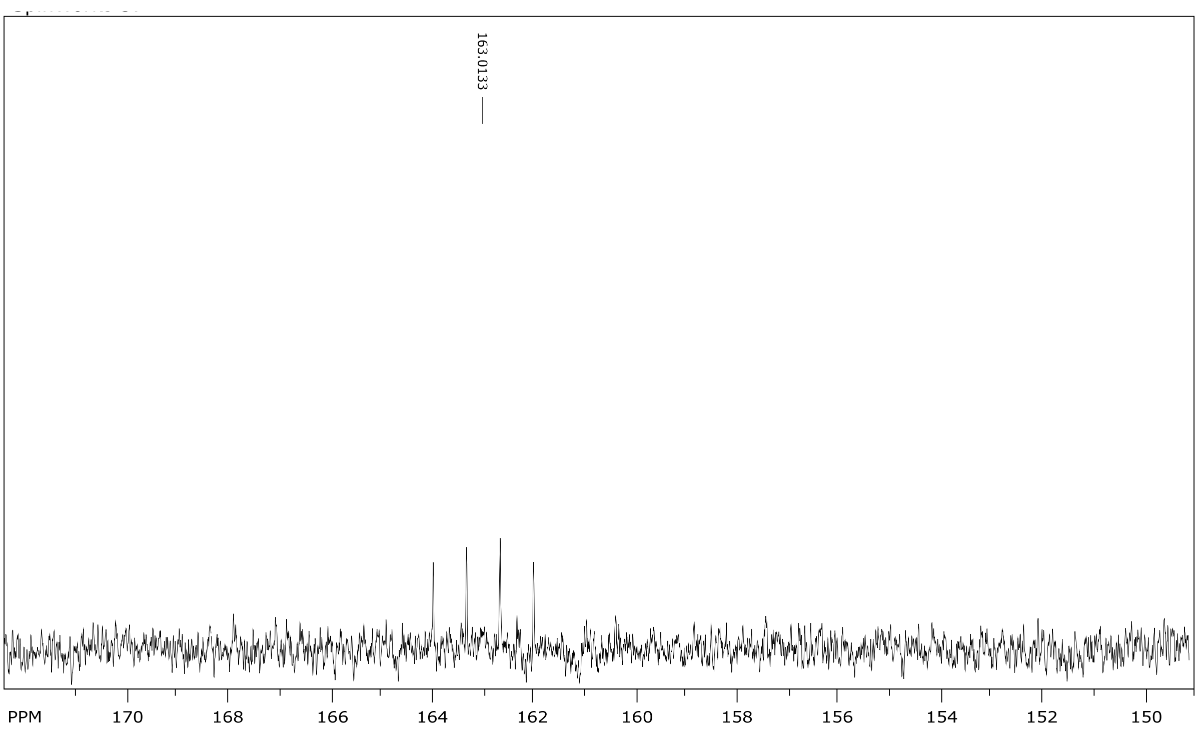

Figure S61. ^{13}C-NMR spectrum (expanded region) of [Cp*(CO)Ir{P(IDipp)}][BAr^F] (**8**) in THF-d_8 at room temperature.

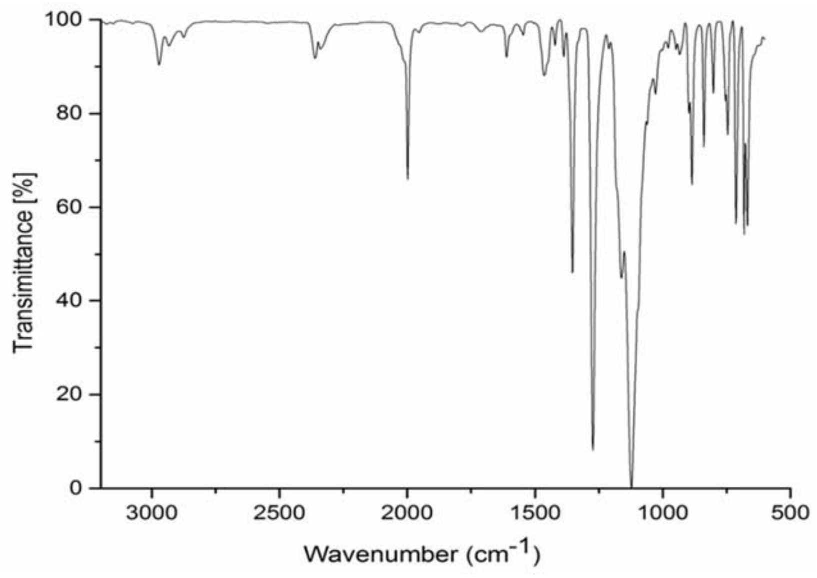

Figure S62. IR spectrum of [(η^5-C_5Me_5){(IDipp)P}Ir(CO)][BAr^F] (**8**) as a neat solid (ATR mode).

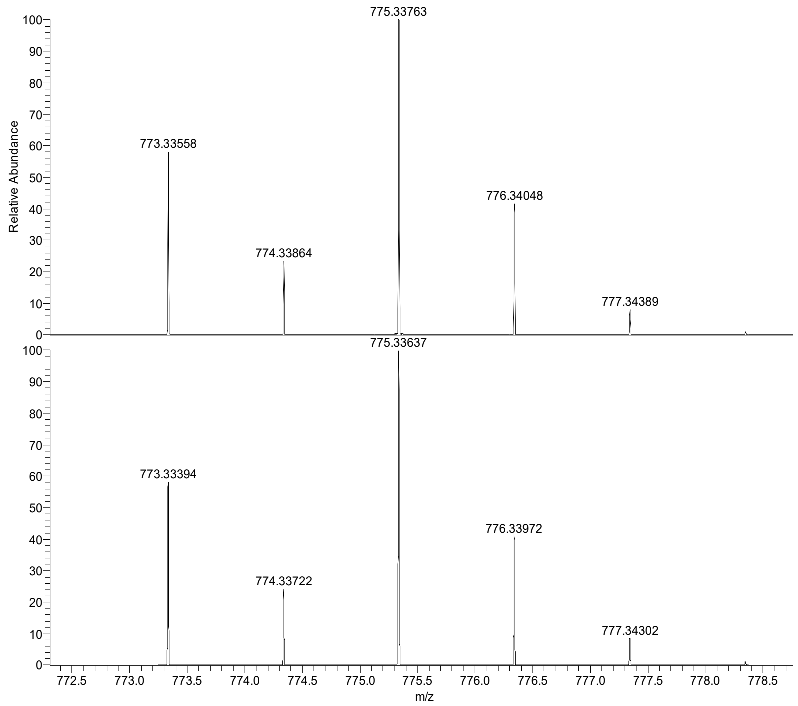

Figure S63. HRMS (ESI) of the complex [(η^5-C_5Me_5){(IDipp)P}Ir(CO)][BAr^F] (**8**) in positive mode, shows the cationic part of the complex (top: experimental isotopic pattern, bottom: computed isotopic pattern).

B. Crystallographic Data

Crystals were mounted on a glass fibre (**4**-**9**) or on top of a human hair (**4a**) with per-fluorinated inert oil. Data were recorded on Oxford Diffraction Xcalibur diffractometers equipped with a Cu-microfocus source Nova and an Atlas CCD detector (**4a**, **7**) or an enhance Mo finefocus sealed tube and an Eos CCD detector (**4b**, **5**, **8** and **9**). Data reduction was performed with CrysalisPro [1]. Absorption correction was based on multi-scans and in case of compound **4a** and **9** additionally face indexation and integration on a Gaussian grid was applied. The structures were solved by either direct methods or intrinsic phasing with SHELXS [2] (**4a**, **5**, **8**) or SHELXT [3] (**4b**, **7**, **9**) and refined on F^2 using the program SHELXL [4] in WinGX v2014.1[5] and additionally OLEX^2 [6] was employed. H atoms were placed in idealized positions and refined using a riding model.

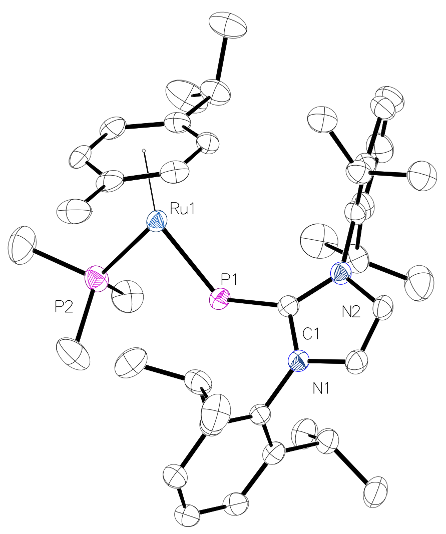

Figure S64: Molecular structure of the cationic complex in **4a** with thermal displacement parameters drawn at the 50% probability level. Hydrogen atoms, the BAr^F counterion and the solvent molecule were omitted for clarity. Selected bond lengths and angles Ru1–P1 2.1929(7) Å, Ru1–P2 2.3387(8) Å, C1–P1 1.848(3) Å, N1-C1-N2 105.3(2)°, and P1-Ru1-P2 81.88(3)°.

Table S1. Crystal data and structure refinement for compound **4a**.

CCDC	1972563	
Empirical formula	$C_{78}H_{76}BF_{25}N_2P_2Ru$	
Formula weight	1690.22	
Temperature	100(2) K	
Wavelength	1.54184 Å	
Instrument (scan mode)	Oxford Diffraction Xcalibur, Atlas, Nova (ω scan)	
Crystal system	Triclinic	
Space group	$P\bar{1}$	
Unit cell dimensions	a = 12.8649(8) Å	α = 77.109(6)°
	b = 14.9396(10) Å	β = 85.015(4)°
	c = 20.9256(12) Å	γ = 87.530(6)°
Volume	3904.5(4) Å^3	
Z	2	
Density (calculated)	1.438 Mg/m^3	
Absorption coefficient	2.928 mm^{-1}	
F(000)	1724	
Crystal habitus	irregular (dichroic red and green)	
Crystal size	0.11 x 0.07 x 0.04 mm^3	
θ range for data collection	3.321 to 76.702°	
Index ranges	-16 ≤ h ≤ 16, -18 ≤ k ≤ 18, -26 ≤ l ≤ 26	
Reflections collected	148534	
Independent reflections	16277 [R_{int} = 0.1176]	
Completeness to θ = 67.684°	100.0 %	
Absorption correction	Gaussian	
Refinement method	Full-matrix least-squares on F^2	
Data / restraints / parameters	16277 / 56 / 1073	
Goodness-of-fit on F^2	1.026	
Final R indices [I > σ(I)]	R_1 = 0.0468, wR_2 = 0.1100	
R indices (all data)	R_1 = 0.0659, wR_2 = 0.1212	
Largest diff. peak and hole	0.889 and -1.107 e·Å^{-3}	

Refinement Details: Four CF_3-groups and a fluorobenzene molecule could be refined with a split atom disorder model. One of these CF_3-groups was refined disordered over three positions. C–F and F–F distances in all disordered CF_3-groups were restrained. The fluorobenzene molecule was found disordered over three positions, but only the resulting three fluorine position were refined as such. The alternative carbon atom positions are very close to each other and therefore, we decided to exclude them from the disorder model.

Table S2. Crystal data and structure refinement for compound **4b**.

CCDC	1972564	
Empirical formula	$C_{76}H_{79}BF_{24}N_2OOsP_2$	
Formula weight	1755.36	
Temperature	100(2) K	
Wavelength	0.71073 Å	
Instrument (scan mode)	Oxford Diffraction, Xcalibur, EOS (ω scan)	
Crystal system	Triclinic	
Space group	$P\bar{1}$	
Unit cell dimensions	a = 12.8702(4) Å	α = 107.209(2)°
	b = 14.8183(4) Å	β = 99.113(3)°
	c = 21.4547(7) Å	γ = 91.429(2)°
Volume	3848.0(2) Å^3	
Z	2	
Density (calculated)	1.515 Mg/m^3	
Absorption coefficient	1.801 mm^{-1}	
F(000)	1768	
Crystal habitus	plate (brown)	
Crystal size	0.24 x 0.17 x 0.06 mm^3	
θ range for data collection	2.238 to 29.566°	
Index ranges	-17 ≤ h ≤ 17, -19 ≤ k ≤ 20, -28 ≤ l ≤ 29	
Reflections collected	166003	
Independent reflections	20048 [R_{int} = 0.0726]	
Completeness to θ = 25.242°	99.9 %	
Absorption correction	Semi-empirical from equivalents	
Refinement method	Full-matrix least-squares on F^2	
Data / restraints / parameters	20048 / 0 / 978	
Goodness-of-fit on F^2	1.040	
Final R indices [I > σ(I)]	R_1 = 0.0434, wR_2 = 0.0879	
R indices (all data)	R_1 = 0.0641, wR_2 = 0.0957	
Largest diff. peak and hole	1.713 and -1.090 e·Å^{-3}	

Table S3. Crystal data and structure refinement for compound **5**.

CCDC	1972565	
Empirical formula	$C_{76}H_{74}BF_{24}N_4OsP$	
Formula weight	1731.37	
Temperature	100(2) K	
Wavelength	0.71073 Å	
Instrument (scan mode)	Oxford Diffraction Xcalibur, Eos (ω scan)	
Crystal system	Triclinic	
Space group	$P\bar{1}$	
Unit cell dimensions	a = 13.7801(4) Å	α = 104.530(2)°
	b = 14.3018(4) Å	β = 96.958(2)°
	c = 20.1335(4) Å	γ = 92.273(2)°
Volume	3802.57(17) $Å^3$	
Z	2	
Density (calculated)	1.512 Mg/m^3	
Absorption coefficient	1.801 mm^{-1}	
F(000)	1740	
Crystal habitus	block (red)	
Crystal size	0.21 x 0.19 x 0.15 mm^3	
θ range for data collection	2.174 to 29.130°	
Index ranges	-18 ≤ h ≤ 18, -19 ≤ k ≤ 19, -27 ≤ l ≤ 27	
Reflections collected	215202	
Independent reflections	20441 [R_{int} = 0.0467]	
Completeness to θ = 25.242°	99.9 %	
Absorption correction	Semi-empirical from equivalents	
Refinement method	Full-matrix least-squares on F^2	
Data / restraints / parameters	20441 / 36 / 1018	
Goodness-of-fit on F^2	1.048	
Final R indices [I > σ(I)]	R_1 = 0.0265, wR_2 = 0.0595	
R indices (all data)	R_1 = 0.0317, wR_2 = 0.0618	
Largest diff. peak and hole	1.570 and -1.007 e·$Å^{-3}$	
Refinement Details: Three CF_3-groups were found disordered and refined with a split atom model. Restraints were applied on the C–F and F–F distances.		

Table S4. Crystal data and structure refinement for compound **7**.

CCDC	1972566	
Empirical formula	$C_{76}H_{75}B_1F_{24}IrN_4P$	
Formula weight	1734.38	
Temperature	101(2) K	
Wavelength	1.54184 Å	
Instrument (scan mode)	Oxford Diffraction, Xcalibur, Atlas, Nova (ω scan)	
Crystal system	Monoclinic	
Space group	$P2_1$	
Unit cell dimensions	a = 12.3621(3) Å	α = 90°
	b = 39.0940(14) Å	β = 94.494(2)°
	c = 15.9803(4) Å	γ = 90°
Volume	7699.3(4) $Å^3$	
Z	4	
Density (calculated)	1.496 Mg/m^3	
Absorption coefficient	4.460 mm^{-1}	
F(000)	3488	
Crystal habitus	plate (brown)	
Crystal size	0.12 x 0.05 x 0.04 mm^3	
θ range for data collection	3.579 to 76.287°	
Index ranges	-15 ≤ h ≤ 15, -47 ≤ k ≤ 49, -20 ≤ l ≤ 12	
Reflections collected	83424	
Independent reflections	30838 [R_{int} = 0.0764]	
Completeness to θ = 67.684°	99.6 %	
Absorption correction	Semi-empirical from equivalents	
Refinement method	Full-matrix least-squares on F^2	
Data / restraints / parameters	30838 / 169 / 2046	
Goodness-of-fit on F^2	1.033	
Final R indices [I > σ(I)]	R_1 = 0.0566, wR_2 = 0.1140	
R indices (all data)	R_1 = 0.0664, wR_2 = 0.1187	
Absolute structure parameter	0.370(7)	
Largest diff. peak and hole	4.051 and -2.015 e·$Å^{-3}$	

Refinement Details: The crystal is twinned by inversion and refined accordingly. The twin factor refined to 0.370(7). Three disordered CF_3 groups were refined using split atom models. The disordered fluorine atoms were restraint to approx. isotropic behaviour (ISOR). The ring carbon atoms of the Cp* moieties were restraint to approx. isotropic behaviour (ISOR).

Table S5. Crystal data and structure refinement for compound **8**.

CCDC	1972567	
Empirical formula	$C_{70}H_{63}BF_{24}IrN_2OP$	
Formula weight	1638.20	
Temperature	100(2) K	
Wavelength	0.71073 Å	
Instrument (scan mode)	Oxford Diffraction Xcalibur, Eos (ω scan)	
Crystal system	Monoclinic	
Space group	$P2_1/n$	
Unit cell dimensions	a = 12.7042(2) Å	α = 90°
	b = 15.8399(2) Å	β = 98.283(2)°
	c = 35.8062(6) Å	γ = 90°
Volume	7130.23(19) $Å^3$	
Z	4	
Density (calculated)	1.526 Mg/m^3	
Absorption coefficient	2.001 mm^{-1}	
F(000)	3272	
Crystal habitus	block (red)	
Crystal size	0.31 x 0.21 x 0.08 mm^3	
θ range for data collection	2.189 to 28.698°	
Index ranges	$-17 \leq h \leq 17$, $-21 \leq k \leq 21$, $-48 \leq l \leq 48$	
Reflections collected	185304	
Independent reflections	18417 [R_{int} = 0.0565]	
Completeness to θ = 25.242°	99.9 %	
Absorption correction	Semi-empirical from equivalents	
Refinement method	Full-matrix least-squares on F^2	
Data / restraints / parameters	18417 / 30 / 927	
Goodness-of-fit on F^{2sigma}	1.132	
Final R indices [I > σ(I)]	R_1 = 0.0481, wR_2 = 0.0997	
R indices (all data)	R_1 = 0.0559, wR_2 = 0.1029	
Largest diff. peak and hole	5.222 and -2.093 e·$Å^{-3}$	
Refinement Details: One disordered CF_3 group was refined using a split atom model with restrained C–F and F–F distances.		

Table S6. Crystal data and structure refinement for compound **9**.

CCDC	2012410	
Empirical formula	$C_{72}H_{66}BF_{24}IrN_2P$	
Formula weight	1649.24	
Temperature	100(2) K	
Wavelength	0.71073 Å	
Instrument (scan mode)	Oxford Diffraction Xcalibur, Eos (ω scan)	
Crystal system	Monoclinic	
Space group	$P2_1/c$	
Unit cell dimensions	a = 25.5721(7) Å	α = 90°
	b = 32.3908(8) Å	β = 108.723(3)°
	c = 17.5240(5) Å	γ = 90°
Volume	13747.0(7) $Å^3$	
Z	8	
Density (calculated)	1.594 Mg/m^3	
Absorption coefficient	2.075 mm^{-1}	
F(000)	6600	
Crystal habitus	block (yellow)	
Crystal size	0.41 x 0.16 x 0.09 mm^3	
θ range for data collection	2.143 to 29.599°	
Index ranges	$-32 \le h \le 32$, $-44 \le k \le 41$, $-22 \le l \le 24$	
Reflections collected	269371	
Independent reflections	34427 [R_{int} = 0.1079]	
Completeness to θ = 25.242°	99.9 %	
Absorption correction	Gaussian	
Max. and min. transmission	0.943 and 0.524	
Refinement method	Full-matrix least-squares on F^2	
Data / restraints / parameters	34427 / 459 / 1933	
Goodness-of-fit on F^{2sigma}	1.024	
Final R indices [I > σ(I)]	R_1 = 0.0507, wR_2 = 0.0894	
R indices (all data)	R_1 = 0.0977, wR_2 = 0.1056	
Largest diff. peak and hole	2.943 and -1.397 $e \cdot Å^{-3}$	

Refinement Details: H1 and H2A were refined with a distance restraint (DFIX 1.43) according to calculated positions and refined with a riding model. Several disordered CF3-groups were refined with a discrete disorder model and restrained C–F and F–F distances. Several fluorine atoms as well as two carbon atoms within the BAr^F moiety were restraint to approx. isotropic behaviour (ISOR).

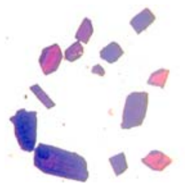

Figure S65. Photograph of the crystals of [(η^6-*p*-cymene){(IDipp)P}Os(^{Me}IMe)][BAr^F] (**5**).

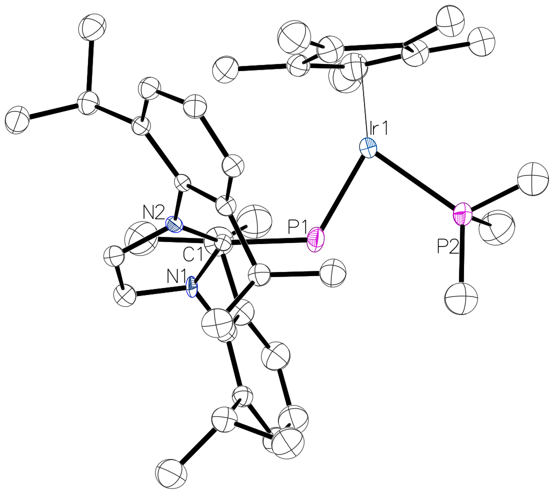

Figure S66. ORTEP diagram of the complex [(η^5-C_5Me_5){(IDipp)P}Ir(PMe_3)][BAr^F] (**6**) with thermal displacement parameters drawn at 50% probability level. The anionic moiety and hydrogen atoms are omitted for clarity.

C. Theoretical Calculations

All computations were performed using the density functional method B97-D (S. Grimme) as implemented in the Gaussian09 program.[9] For all main group elements (C, H, N, O and P) the all-electron triple-ζ basis set 6-311G(d,p)[10] was used and a Stuttgart RSC effective core potential basis set for the 6d transition metals iridium and osmium.[11,12] Natural Bond Orbital (NBO) analysis (NBO charges, WBI) was carried out using NBO version 3,[13] which is part of the Gaussian09 program package. Harmonic vibrational frequencies are calculated to characterize respective minima structures (with no imaginary frequency).

Table S7. Energies for all optimized structures.

		**Compound**		E_{0K}^{a} / [Ha]	E_{298K}^{b} / [Ha]	H_{298K}^{b} / [Ha]	G_{298K}^{b} / [Ha]
osmium complexes	1	[(η^6-*p*-cymene)(Mes*P)Os(PMe_3)] (**I**)	*E*	–1985.291148	–1985.248170	–1985.247226	–1985.363392
			Z	–1985.282621	–1985.239803	–1985.238858	–1985.353907
			**Δ*E*$_{(Z-E)}$** / [kcal mol^{-1}]	**5.3**	**5.2**	**5.2**	**5.9**
	2	[(η^6-*p*-cymene){(IDipp)P}Os(PMe_3)]$^+$ (in **4b**)	*E*	–2441.455552	–2441.402654	–2441.401710	–2441.540857
			Z	–2441.441311	–2441.388603	–2441.387659	–2441.526999
			**Δ*E*$_{(Z-E)}$** / [kcal mol^{-1}]	**8.9**	**8.8**	**8.8**	**8.7**
	3	[(η^6-*p*-cymene){(IDipp)P}Os(^{Me}IMe)]$^+$ (in **5**)	*E*	–2363.616379	–2363.560239	–2363.559295	–2363.705223
			Z	–2363.605072	–2363.549010	–2363.548066	–2363.693803
			**Δ*E*$_{(Z-E)}$** / [kcal mol^{-1}]	**7.1**	**7.0**	**7.0**	**7.2**
	4	[(η^6-*p*-cymene){(IDipp)P}Os(CO)]$^+$	*E*	–2093.763539	–2093.716547	–2093.715603	–2093.842158
			Z	–2093.774395	–2093.726891	–2093.725947	–2093.855229
			**Δ*E*$_{(Z-E)}$** / [kcal mol^{-1}]	**–6.8**	**–6.5**	**–6.5**	**–8.2**
iridium complexes	5	[(η^5-C_5Me_5)(Mes*P)Ir(PMe_3)] (**II**)	*E*	–1999.4012	–1999.3572	–1999.3562	–1999.4724
			Z	–1999.4011	–1999.3569	–1999.3560	–1999.4730
			**Δ*E*$_{(Z-E)}$** / [kcal mol^{-1}]	**0.1**	**0.2**	**0.2**	**–0.3**
	6	[(η^5-C_5Me_5){(IDipp)P}Ir(PMe_3)]$^+$ (in **6**)	*E*	–2455.558031	–2455.504504	–2455.503559	–2455.642517
			Z	–2455.561421	–2455.507591	–2455.506647	–2455.647196
			**Δ*E*$_{(Z-E)}$** / [kcal mol^{-1}]	**–2.1**	**–1.9**	**–1.9**	**–2.9**
	7	[(η^5-C_5Me_5){(IDipp}P}Ir(^{Me}IMe)]$^+$ (in **7**)	*E*	–2377.723773	–2377.666221	–2377.665276	–2377.813600
			Z	–2377.726779	–2377.668836	–2377.667892	–2377.817265
			**Δ*E*$_{(Z-E)}$** / [kcal mol^{-1}]	**–1.9**	**–1.6**	**–1.6**	**–2.3**
	8	[(η^5-C_5Me_5){(IDipp)P}Ir(CO)]$^+$ (in **8**)	*E*	–2107.864916	–2107.816230	–2107.815286	–2107.945318
			Z	–2107.886427	–2107.837463	–2107.836518	–2107.967393
			**Δ*E*$_{(Z-E)}$** / [kcal mol^{-1}]	**–13.5**	**–13.3**	**–13.3**	**–13.8**
	9	[(η^5-C_5Me_5){(η^3-benzyl-IDipp)PH}Ir]$^+$ (in **9**)	PH adjacent	–1994.566245	–1994.520840	–1994.519896	–1994.639587
			PH opposite	–1994.565797	–1994.520321	–1994.519377	–1994.639673
			**Δ*E*$_{(opposite-adjacent)}$** / [kcal mol^{-1}]	**0.3**	**0.3**	**0.3**	**–0.1**
phosphinidene ligands	10	C_6H_2(*t*Bu)$_3$P	triplet	–1043.963393	–1043.940900	–1043.939955	–1044.013912
			singlet	–1043.950872	–1043.928317	–1043.927372	–1044.000715
			**Δ*E*$_{(singlet-triplet)}$** / [kcal mol^{-1}]	**7.9**	**7.9**	**7.9**	**8.3**
	11	[(IDipp)P]$^+$	triplet	–1500.091502	–1500.058769	–1500.057824	–1500.158557
			singlet	–1500.078166	–1500.045365	–1500.044421	–1500.144227
			**Δ*E*$_{(singlet-triplet)}$** / [kcal mol^{-1}]	**8.4**	**8.4**	**8.4**	**9.0**

^{a} DFT energy incl. ZPE.
^{b} standard conditions T = 298.15 K and p = 1 atm.

Table S8a. NBO analysis of osmium complex of type **I** and the cation in osmium complex **4b**.

	NBO no.	type	element	NBO charge	WBI	coefficent	localization [%]	AO contribution [%]		
								s	p	d
(*E*)-[(η ^{6}-p-cymene)(Mes*P)Os(PMe$_3$)] (**I**)	136	π(Os-PC$_6$H$_2$(*t*Bu)$_3$)	Os			0,77	59	2,4	5,7	91,9
			P			0,64	41	1,3	98,5	0,3
	132	σ(Os-PC$_6$H$_2$(*t*Bu)$_3$)	Os	–0.85	1.55	0.69	48	36.3	12.0	51.8
			P	0.39		0.72	52	22.5	77.2	0.3
	131	σ(Os-PMe$_3$)	Os		0.81	0.61	38	35.9	19.0	45.1
			P	1.26		0.79	62	36.7	63.2	0.2
	130	LP(P{IDipp})	P			——	100	63.0	37.0	0
(*E*)-[(η ^{6}-p-cymene){(IDipp)P}Os(PMe$_3$)]$^+$ (in **4b**)	174	π(Os-P{IDipp})	Os			0.69	47	11.6	17.4	71.0
			P			0.72	53	4.4	95.4	0.2
	163	σ(Os-P{IDipp})	Os	–0.79	1.53	0.66	43	38.3	15.5	46.3
			P	0.32		0.75	57	21.5	78.2	0.3
	162	σ(Os-PMe$_3$)	Os		0.78	0.58	33	26.3	48.1	25.6
			P	1.25		0.82	67	34.2	65.7	0.2
	161	LP(P{IDipp})	P			——	100	62.9	37.1	0

(*E*)-[(η^6-p-cymene)(Mes*P)Os(PMe_3)] (**I**)

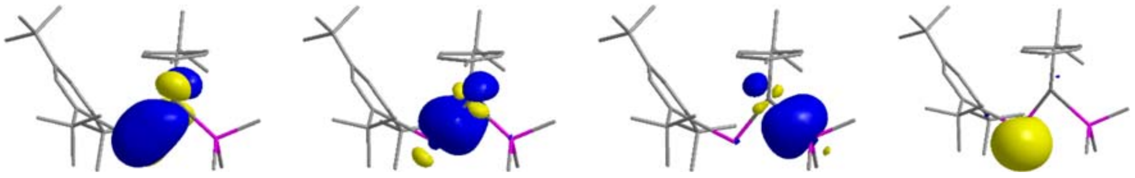

NBO #136 **NBO #132** **NBO #131** **NBO #130**

(*E*)-[(η^6-p-cymene){IDipp)P}Os(PMe_3)]$^+$ (in **4b**)

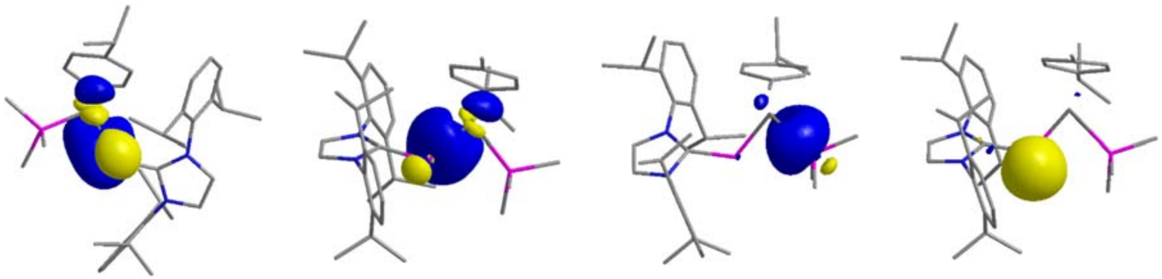

NBO #174 **NBO #163** **NBO #162** **NBO #161**

Table S8b. NBO analysis of the cation in osmium complex **5** and in [(η^6-p-cymene){IDipp)P}Os(CO)]$^+$.

	NBO no.	type	element	NBO charge	WBI	coefficent	localization [%]	AO contribution [%] s	p	d
(*E*)-[(η^6-p-cymene){IDipp)P}Os(^{Me}IMe)]$^+$ (in **5**)	180	π(Os-P{IDipp})	Os			0.70	48	14.9	8.3	76.9
			P			0.72	52	7.0	92.8	0.2
	173	σ(Os-P{IDipp})	Os	–0.58	1.50	0.69	47	28.6	11.3	60.1
			P	0.33		0.73	53	17.7	82.1	0.3
	172	σ(Os-{^{Me}IMe})	Os		0.76	0.59	34	32.9	14.0	53.1
			C	0.35		0.81	66	43.8	56.2	0
	171	LP(P{IDipp})	P			——	100	64.2	35.8	0
(*Z*)-[(η^6-p-cymene){IDipp)P}Os(CO)]$^+$	154	π(Os-PIPr)	Os			0.76	58	1.7	5.4	92.9
			P			0.65	42	0.7	99.0	0.3
	149	σ(Os-PIPr)	Os	–0.70	1.54	0.69	47	37.3	13.1	49.6
			P	0.47		0.72	53	19.4	80.3	0.3
	148	π(C-O)	C		2.04	0.50	25	0.2	99.1	0.66
			O	–0.45		0.87	75	0.2	99.7	0.1
	103	LP(PIPr)	P			——	100	67.4	32.6	0
	85	σ(Os-CO)	Os		1.30	0.61	37	34.6	17.0	48.5
			C	0.66		0.79	63	65.2	34.8	0
	78	π(C-O)	C			0.52	28	11.7	87.8	0.5
			O			0.85	72	14.2	85.7	0.1

(*E*)-[(η^6-p-cymene){IDipp)P}Os(^{Me}IMe)]$^+$ (in **5**)

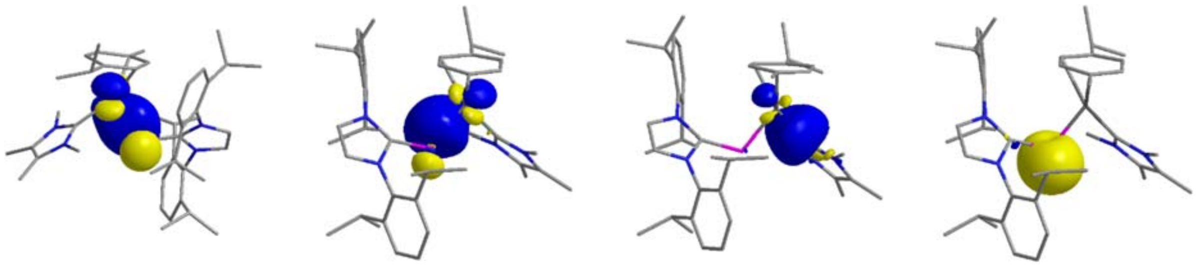

NBO #180 **NBO #173** **NBO #172** **NBO #171**

(*Z*)-[(η^6-p-cymene){IDipp)P}Os(CO)]$^+$

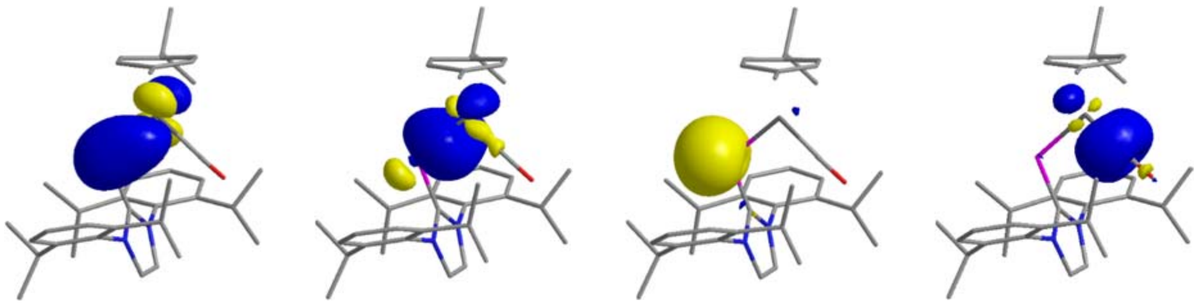

NBO #154 **NBO #149** **NBO #103** **NBO #185**

Table S9a. NBO analysis of iridium complex of type **II** and the cation in iridium complex **6**.

	NBO no.	type	element	NBO charge	WBI	coefficent	localization [%]	AO contribution [%]		
								s	p	d
(*E*)-[(η^5-C_5Me_5)(Mes*P)Ir(PMe_3)] (**II**)	136	π(Ir-PC_6H_2(*t*Bu)$_3$)	Ir			0.81	66	0	4.0	96.0
			P			0.59	34	0	99.6	0.3
	133	σ(Ir-PC_6H_2(*t*Bu)$_3$)	Ir	–0.61	1.40	0.69	48	40.9	10.0	49.1
			P	0.36		0.72	52	20.7	79.0	0.4
	132	σ(Ir-PMe_3)	Ir		0.80	0.62	38	37.7	15.7	46.6
			P	1.23		0.78	62	35.7	64.2	0.2
	131	LP(P{IDipp})	P			——	100	65.3	34.6	0
(*Z*)-[(η^5-C_5Me_5){(IDipp)P}Ir(PMe_3)]$^+$ (in **6**)	168	π(Ir-P{IDipp})	Ir			0.74	55	10.4	6.2	83.4
			P			0.67	45	5.1	94.6	0.3
	164	σ(Ir-P{IDipp})	Ir	–0.60	1.40	0.70	49	26.5	9.6	63.9
			P	0.32		0.71	51	14.1	85.7	0.3
	163	σ(Ir-PMe_3)	Ir		0.82	0.63	39	39.3	16.7	44.1
			P	1.23		0.78	61	34.1	65.7	0.2
	162	LP(P{IDipp})	P			——	100	68.5	31.5	0

(*E*)-[(η^5-C_5Me_5)(Mes*P)Ir(PMe_3)] (**II**)

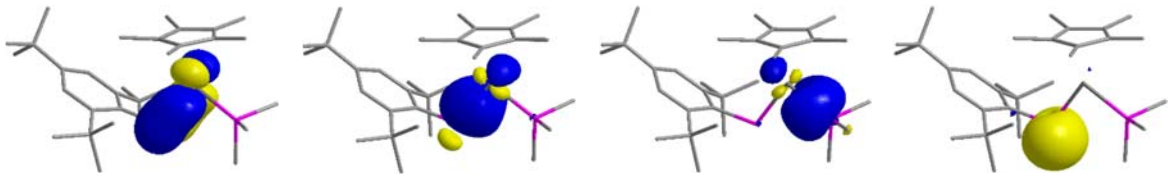

NBO #136 **NBO #133** **NBO #132** **NBO #131**

(*Z*)-[(η^5-C_5Me_5){(IDipp)P}Ir(PMe_3)]$^+$ (in **6**)

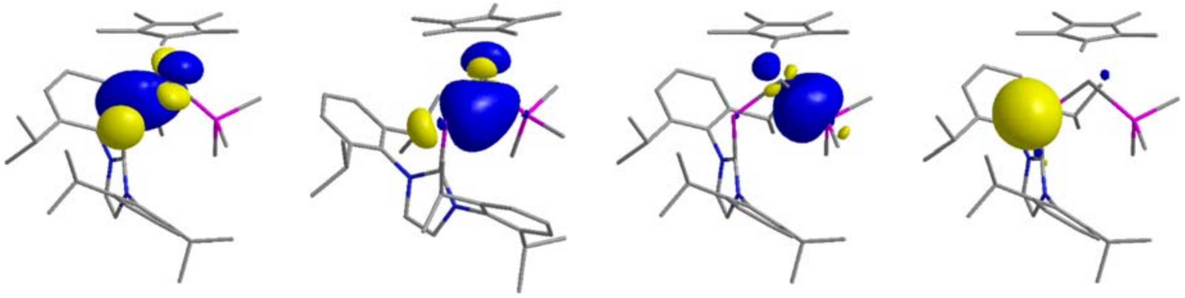

NBO #168 **NBO #164** **NBO #163** **NBO #162**

Table S9b. NBO analysis of the cations in iridium complexes **7** and **8**.

	NBO no.	type	element	NBO charge	WBI	coefficent	localization [%]	AO contribution [%] s	p	d
(*Z*)-[(η^5-C_5Me_5){(IDipp)P}Ir(^{Me}IMe)]$^+$ (in **7**)	187	π(Ir-P{IDipp})	Ir			0.76	57	2.5	5.0	92.5
			P			0.65	43	1.3	98.5	0.2
	174	σ(Ir-P{IDipp})	Ir	–0.39	1.40	0.67	45	40.6	11.0	48.4
			P	0.27		0.74	55	19.3	80.4	0.3
	173	σ(Ir-{^{Me}IMe})	Ir		0.78	0.59	35	35.7	11.7	52.7
			C	0.32		0.81	65	43.0	57.0	0
	172	LP(P{IDipp})	P			——	100	66.7	33.3	0
(*Z*)-[(η^5-C_5Me_5){(IDipp)P}Ir(CO)]$^+$ (in **8**)	155	π(Ir-P{IDipp})	Ir			0.8	64	1.3	4.5	94.2
			P			0.6	36	0.5	99.2	0.3
	150	σ(Ir-P{IDipp})	Ir	–0.46	1.35	0.71	50	38.0	10.5	51.5
			P	0.41		0.71	50	16.8	82.9	0.4
	149	π(CO)	C		2.05	0.55	30	28.5	71.3	0.3
			O	–0.44		0.84	70	36.4	63.5	0.1
	99	LP(P{IDipp})	P			——	100	70.0	30.0	0
	88	π(CO)	C			0.50	25	0.4	98.9	0.7
			O			0.87	75	0.5	99.4	0.1
	82	σ(Ir-CO)	Ir		1.23	0.60	36	36.9	16.1	47.0
			C	0.62		0.80	64	64.7	35.3	0

(*Z*)-[(η^5-C_5Me_5){(IDipp}P}Ir(^{Me}IMe)]$^+$ (in **7**)

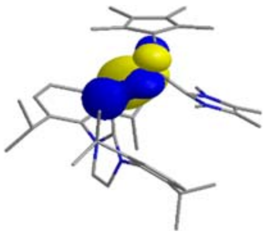

NBO #187

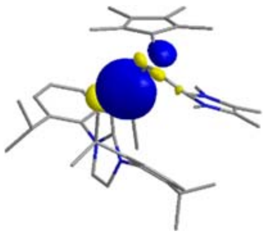

NBO #174

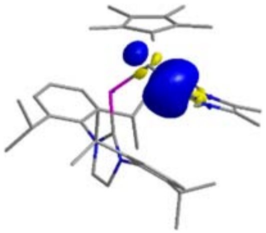

NBO #173

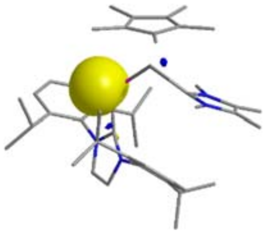

NBO #172

(*Z*)-[(η^5-C_5Me_5){(IDipp)P}Ir(CO)]$^+$ (in **8**)

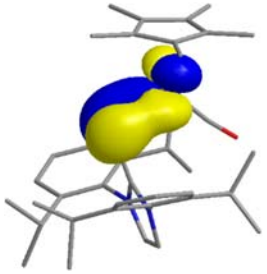

NBO #155

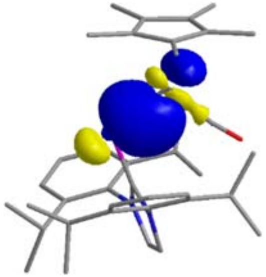

NBO #150

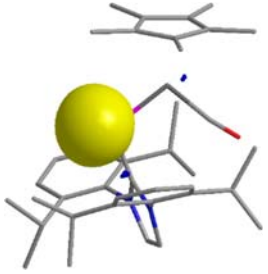

NBO #99

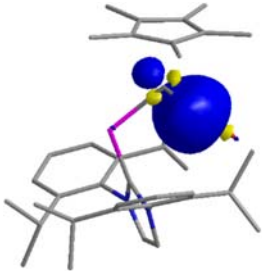

NBO #82

D. References

[1] Rigaku Oxford Diffraction, CrysAlisPRO Software System, versions 1.171.38.43, 1.171.37.35 and 1.171.40.81a Rigaku Corporation, Oxford, UK.

[2] G. M. Sheldrick, *Acta Cryst.*, **2018**, *A64*, 112-122.

[3] G. M. Sheldrick, *Acta Cryst.*, **2015**, *A71*, 3-8.

[4] G. M. Sheldrick, *Acta Cryst.*, **2015**, *C71*, 3-8.

[5] L. J. Farrugia, *J. Appl. Crystallogr*. **2012**, *45*, 849- 854.

[6] O. V. Dolomanov, L. J. Bourhis, R. J. Gildea, J. A. K. Howard, H. Puschmann, *J. Appl. Cryst.*, **2009**, *42*, 339-341.

[7] A. Doddi, D. Bockfeld, T. Bannenberg, Peter. G. Jones and M. Tamm, *Angew. Chem. Int. Ed.* **2014***, 53,* 13568-1357; *Angew. Chem.* **2014**, *126*, 13786-13790.

[8] M. Peter, A. Doddi, T. Bannenberg, M. Freytag, P. G. Jones and M. Tamm, *Inorg. Chem.*, **2017**, *56*, 10785-10793.

[9] Gaussian 09, Revision D.01, M. J. Frisch, G. W. Trucks, H. B. Schlegel, G. E. Scuseria, M. A. Robb, J. R. Cheeseman, G. Scalmani, V. Barone, G. A. Petersson, H. Nakatsuji, X. Li, M. Caricato, A. Marenich, J. Bloino, B. G. Janesko, R. Gomperts, B. Mennucci, H. P. Hratchian, J. V. Ortiz, A. F. Izmaylov, J. L. Sonnenberg, D. Williams-Young, F. Ding, F. Lipparini, F. Egidi, J. Goings, B. Peng, A. Petrone, T. Henderson, D. Ranasinghe, V. G. Zakrzewski, J. Gao, N. Rega, G. Zheng, W. Liang, M. Hada, M. Ehara, K. Toyota, R. Fukuda, J. Hasegawa, M. Ishida, T. Nakajima, Y. Honda, O. Kitao, H. Nakai, T. Vreven, K. Throssell, J. A. Montgomery, Jr., J. E. Peralta, F. Ogliaro, M. Bearpark, J. J. Heyd, E. Brothers, K. N. Kudin, V. N. Staroverov, T. Keith, R. Kobayashi, J. Normand, K. Raghavachari, A. Rendell, J. C. Burant, S. S. Iyengar, J. Tomasi, M. Cossi, J. M. Millam, M. Klene, C. Adamo, R. Cammi, J. W. Ochterski, R. L. Martin, K. Morokuma, O. Farkas, J. B. Foresman, and D. J. Fox, Gaussian, Inc., Wallingford CT, **2016**.

[10] X. Cao, M. Dolg, *J. Chem. Phys.,* **2001**, *115*, 7348.

[11] D. Andrae, U. Haussermann, M. Dolg, H. Stoll, H. Preuss, *Theor. Chim. Acta,* **1990**, *77*, 123.

[12] Stuttgart RSC 1997 ECP Basis set (for Ir and Os) were obtained from the Extensible Computational Chemistry Environment Basis Set Database, Version 1.2.2 [https://bse.pnl.gov/bse/portal]. a) The Role of Databases in Support of Computational Chemistry Calculations, D. Feller, *J. Comp. Chem.,* **1996**, *17*, 1571; b) Basis Set Exchange: A Community Database for Computational Sciences, K. L. Schuchardt, B. T. Didier, T. Elsethagen, L. Sun, V. Gurumoorthi, J. Chase, J. Li and T. L. Windus, *J. Chem. Inf. Model.,* **2007**, *47*, 1045.

[13] a) J. P. Foster, F. Weinhold, *J. Am. Chem. Soc.,* **1980**, *102*, 7211; b) A. E. Reed, F. Weinhold, *J. Chem. Phys.,* **1983**, *78*, 4066; c) A. E. Reed, R. B. Weinstock, F. Weinhold, *J. Chem. Phys.,* **1985**, *83*, 735; d) A. E. Reed, F. Weinhold, *J. Chem. Phys.,* **1985**, *83*, 1736.
